# Supplementary material for: Complete end-to-end learning from protein feature representation to protein interactome inference
Source: Gigascience. 2025 Nov 6;14:giaf122. doi: 10.1093/gigascience/giaf122 (PMC12598752; doi:10.1093/gigascience/giaf122)
Supplement: giaf122_FREEPII_Supplementary_Material_0903 [file giaf122_freepii_supplementary_material_0903.pdf]

Table S1. Average GOGO scores across three ontologies (Biological process (BP), Molecular function (MF), and Cellular component (CC), see Methods for details) for clusters produced by EPIC, SPIFFED, and FREEPII in each experiment. The highest score is highlighted in black bold text.

Ontology	Model	H1	H2	H3	H4	Y1	Y2	Y3	Y4
BP	EPIC	0.252	0.251	0.242	0.236	0.289	0.294	0.301	0.278
	SPIFFED	0.264	0.263	0.251	0.245	0.291	0.295	0.302	0.293
	FREEPII	**0.322**	**0.313**	**0.283**	**0.253**	**0.359**	**0.334**	**0.324**	**0.358**
MF	EPIC	0.550	0.556	0.534	0.532	0.382	0.382	0.392	0.378
	SPIFFED	0.563	0.556	0.534	0.534	0.376	0.372	**0.399**	0.380
	FREEPII	**0.602**	**0.605**	**0.575**	**0.546**	**0.414**	**0.383**	0.375	**0.388**
CC	EPIC	0.642	0.642	0.627	0.622	0.677	**0.679**	0.696	0.673
	SPIFFED	0.650	0.648	0.635	0.632	0.671	0.668	**0.702**	0.670
	FREEPII	**0.688**	**0.684**	**0.651**	**0.635**	**0.683**	0.664	0.666	**0.676**

Table S2. Average co-localization score for clusters produced by EPIC, SPIFFED, and FREEPII in each experiment. The highest score is highlighted in black bold text.

Model	H1	H2	H3	H4	Y1	Y2	Y3	Y4
EPIC	0.641	0.636	0.572	0.543	0.628	0.609	0.647	0.613
SPIFFED	0.693	0.677	0.599	0.608	0.637	0.634	**0.673**	0.635
FREEPII	**0.756**	**0.721**	**0.648**	**0.622**	**0.688**	**0.656**	0.659	**0.696**

Table S3. Average GOGO scores across three ontologies (Biological process (BP), Molecular function (MF), and Cellular component (CC), see Methods for details) for clusters produced by each model in ablation study in each experiment. The highest score is highlighted in black bold text.

Ontology	Model	H1	H2	H3	H4	Y1	Y2	Y3	Y4
BP	RF-C	0.244	0.248	0.231	0.229	0.291	0.279	0.287	0.278
	CNN-C	0.249	0.253	0.241	0.240	0.290	0.277	0.304	0.280
	FREEPII(-)	0.261	0.250	0.248	0.242	0.292	0.284	0.311	0.294
	FREEPII	**0.322**	**0.313**	**0.283**	**0.253**	**0.360**	**0.334**	**0.324**	**0.358**
MF	RF-C	0.550	0.553	0.514	0.523	0.382	0.367	**0.383**	0.365
	CNN-C	0.552	0.553	0.531	0.524	0.371	0.376	0.382	0.378
	FREEPII(-)	0.561	0.554	**0.537**	0.538	0.379	0.380	0.376	0.378
	FREEPII	**0.602**	**0.605**	0.515	**0.546**	**0.414**	**0.383**	0.375	**0.389**
CC	RF-C	0.639	0.643	0.626	0.616	0.668	0.649	**0.686**	0.659
	CNN-C	0.643	0.638	0.625	0.620	0.669	0.666	0.678	0.663
	FREEPII(-)	0.649	0.642	0.631	0.630	0.669	**0.666**	0.677	0.659
	FREEPII	**0.688**	**0.684**	**0.651**	**0.635**	**0.683**	0.664	0.666	**0.676**

Table S4. Average co-localization score for clusters produced by each model in ablation study in each experiment. The highest score is highlighted in black bold text.

Model	H1	H2	H3	H4	Y1	Y2	Y3	Y4
RF-C	0.631	0.646	0.570	0.534	0.646	0.583	0.659	0.585
CNN-C	0.655	0.663	0.590	0.585	0.647	0.611	0.659	0.615
FREEPII(-)	0.688	0.673	0.586	0.598	0.623	0.625	**0.670**	0.608
FREEPII	**0.756**	**0.721**	**0.648**	**0.622**	**0.688**	**0.656**	0.659	**0.696**

Table S5. Classification performance of Tapioca on datasets H1 and Y3. Tapioca was evaluated on our datasets H1 and Y3, which differs in resolution and protein coverage. Despite these differences, Tapioca showed similar performance across both datasets, consistent with the results reported in its original publication (Supplementary Table 1).

Metrics	H1	Y3
Sensitivity	0.042	0.080
Specificity	0.998	0.996
AUC of ROC	0.652	0.611
AUC of PR	0.701	0.671

Table S6. Parameter sizes of each layer in the SPIFFED-like and the FREEPII-like models, using 32 filters in convolution layer as an example. Both models consist of one convolutional layer and three fully connected layers. The size of the convolutional layer's weights in FREEPII-like model is half that of SPIFFED-like model.

Layer	Parameter	SPIFFED-like	FREEPII-like
Convolution layer	Weight	[32, 1, 2, 3]	[32, 1, 3]
	Bias	[32]	[32]
1st fully-connected layer	Weight	[32, 1815]	[32, 1815]
	Bias	[32]	[32]
2nd fully-connected layer	Weight	[16, 32]	[16, 32]
	Bias	[16]	[16]
3rd fully-connected layer	Weight	[1, 16]	[1, 16]
	Bias	[1]	[1]

Table S7. Comparison of memory usage between SPIFFED-like and FREEPII-like models. The table reports the maximum memory usage of each model during training, measured in bytes.

Filters	SPIFFED-like	FREEPII-like	Ratio (SPIFFED-like / FREEPII-like)
32	1.09E+09	7.26E+08	1.507
64	2.26E+09	1.14E+09	1.971
128	3.97E+09	2.32E+09	1.716
256	8.08E+09	4.11E+09	1.966

Table S8. Comparison of time complexity between SPIFFED-like and FREEPII-like models. The table reports the runtime (in seconds) required for each model to start the convergence phase during training.

Filters	SPIFFED-like	FREEPII-like	Ratio (SPIFFED-like / FREEPII-like)
32	73.114	29.470	2.481
64	160.534	51.478	3.119
128	344.961	66.647	5.176
256	641.688	159.393	4.026

Table S9. Parameter sizes of each layer in FREEPII. The architecture includes one embedding layer, one convolutional layer, and three fully connected layers. *N* denotes the number of proteins.

Layer	Parameter	Size
Embedding layer	Weight	[N, 256]
Convolution layer	Weight<br>Bias	[16, 1, 3]<br>[16]
1st fully-connected layer	Weight<br>Bias	[256, 7752]<br>[256]
2nd fully-connected layer	Weight<br>Bias	[64, 256]<br>[64]
3rd fully-connected layer	Weight<br>Bias	[1, 64]<br>[1]

Table S10. Input dimensions of FREEPII for each dataset. The input profile has two dimensions: the number of proteins and the length of the input sequence (CF-MS combined with protein sequence information). The training PPIs input is represented by the number of protein pairs and the indices of the two proteins comprising each pair.

Data	Input profiles	Training PPIs
H1	[4002, 456]	[35158, 2]
H2	[4563, 456]	[39608, 2]
H3	[5268, 456]	[37842, 2]
H4	[6043, 456]	[41500, 2]
Y1	[2397, 456]	[8804, 2]
Y2	[2753, 456]	[10188, 2]
Y3	[2026, 456]	[7194, 2]
Y4	[2952, 456]	[11608, 2]

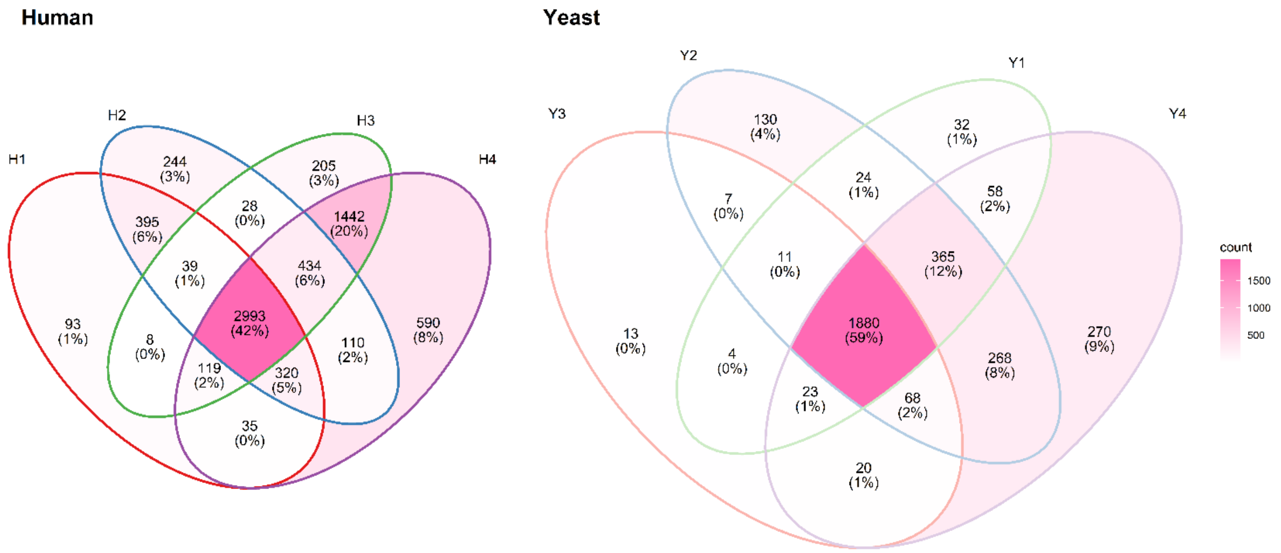

Figure S1. Number of intersected proteins in CF-MS data across various experiments conducted on the same species. Circles of different colors within the figure represent different experiments.

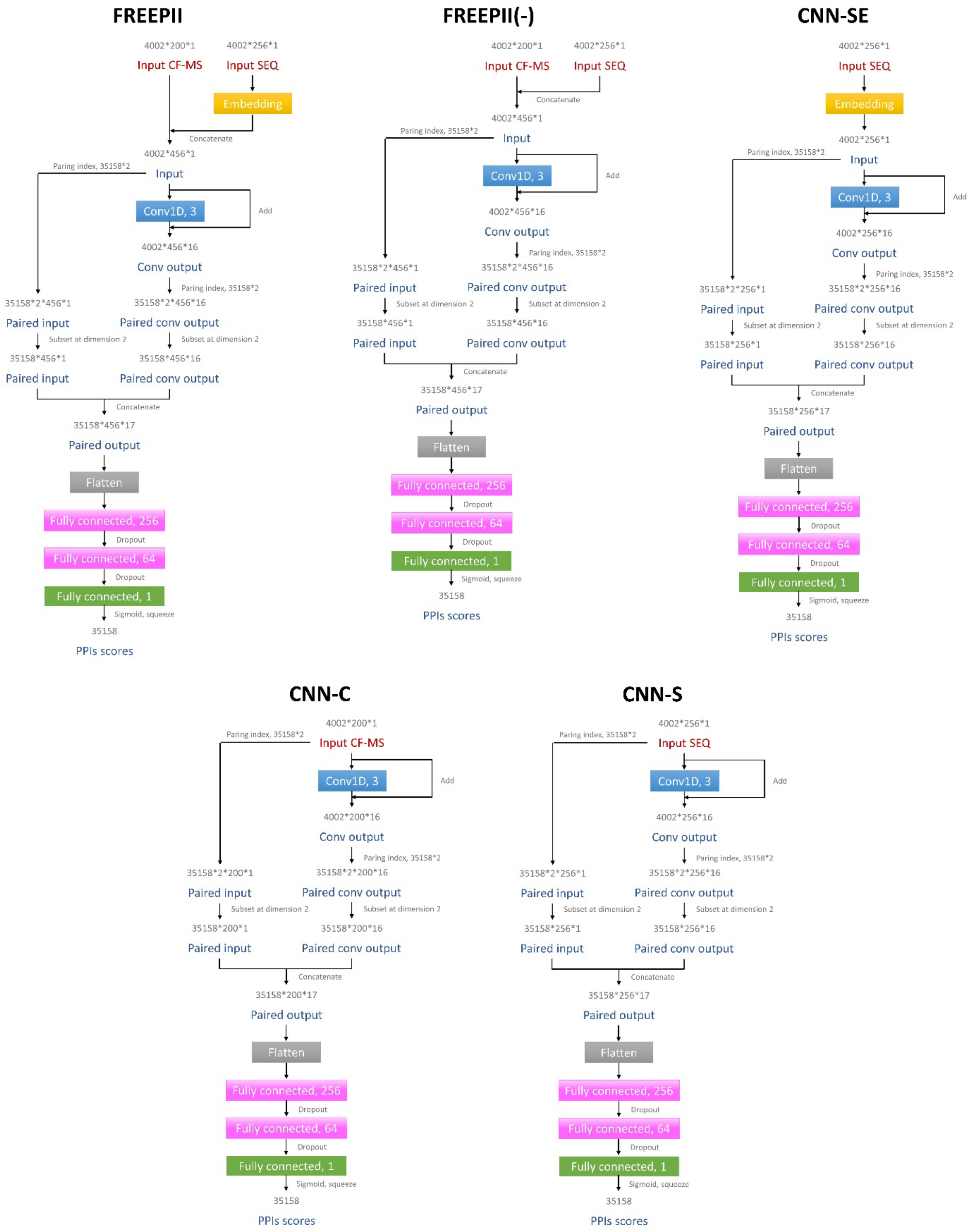

Figure S2. Architecture of models in ablation study. This diagram illustrates the inputs, outputs, and architectures of all models used in the ablation experiment, taking one-fold of training data from the human dataset H1 as an example.

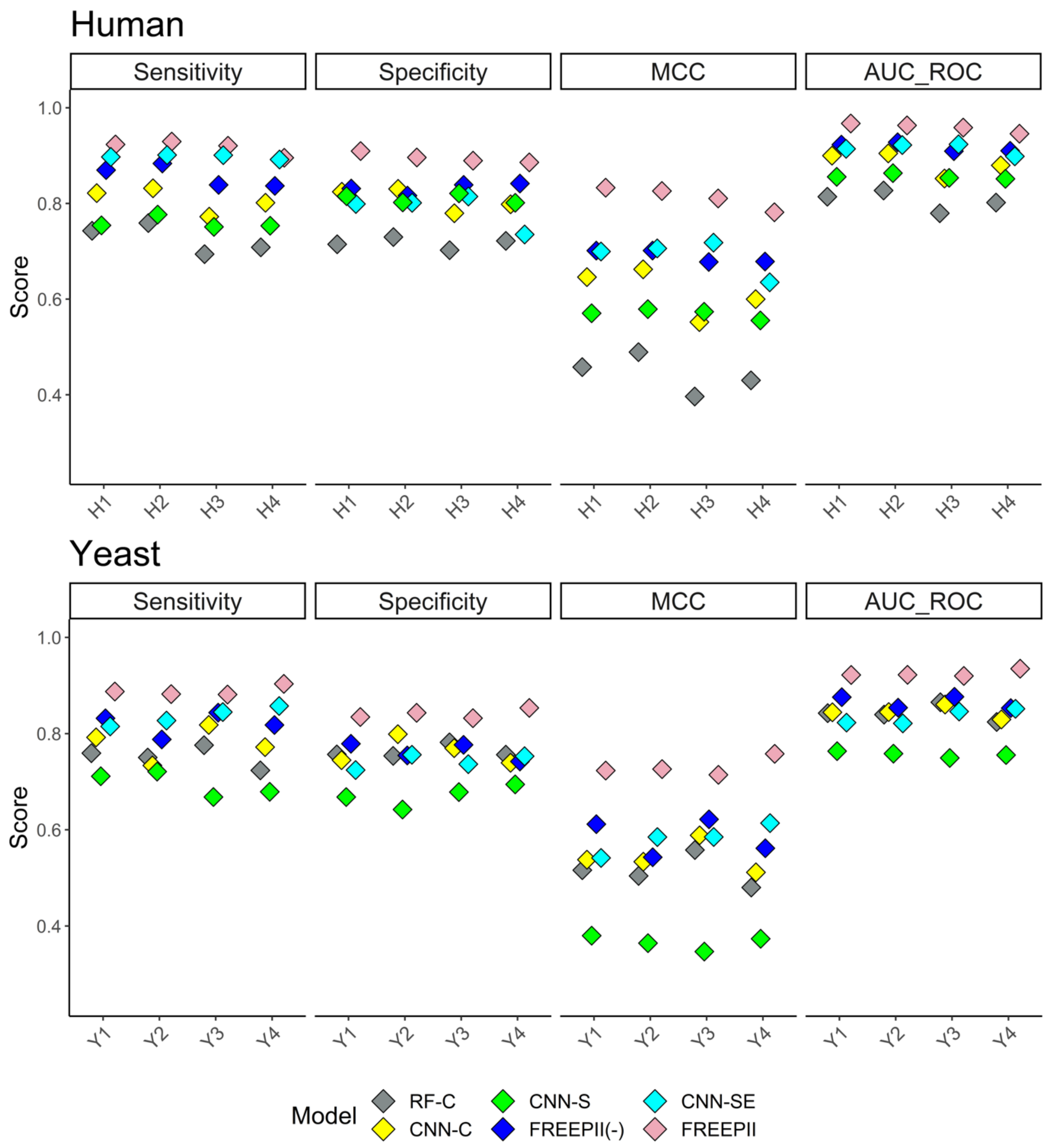

Figure S3. PPI classification performance in ablation study. This extended version of Figure 2B encompasses PPI classification performances for additional models. In particular, it incorporates CNN using only protein sequence as input without protein embeddings (CNN-S) and CNN using both protein sequences and protein embeddings (CNN-SE), along with the previously compared models. All performance metrics are plotted using the models' predictions on the testing set.

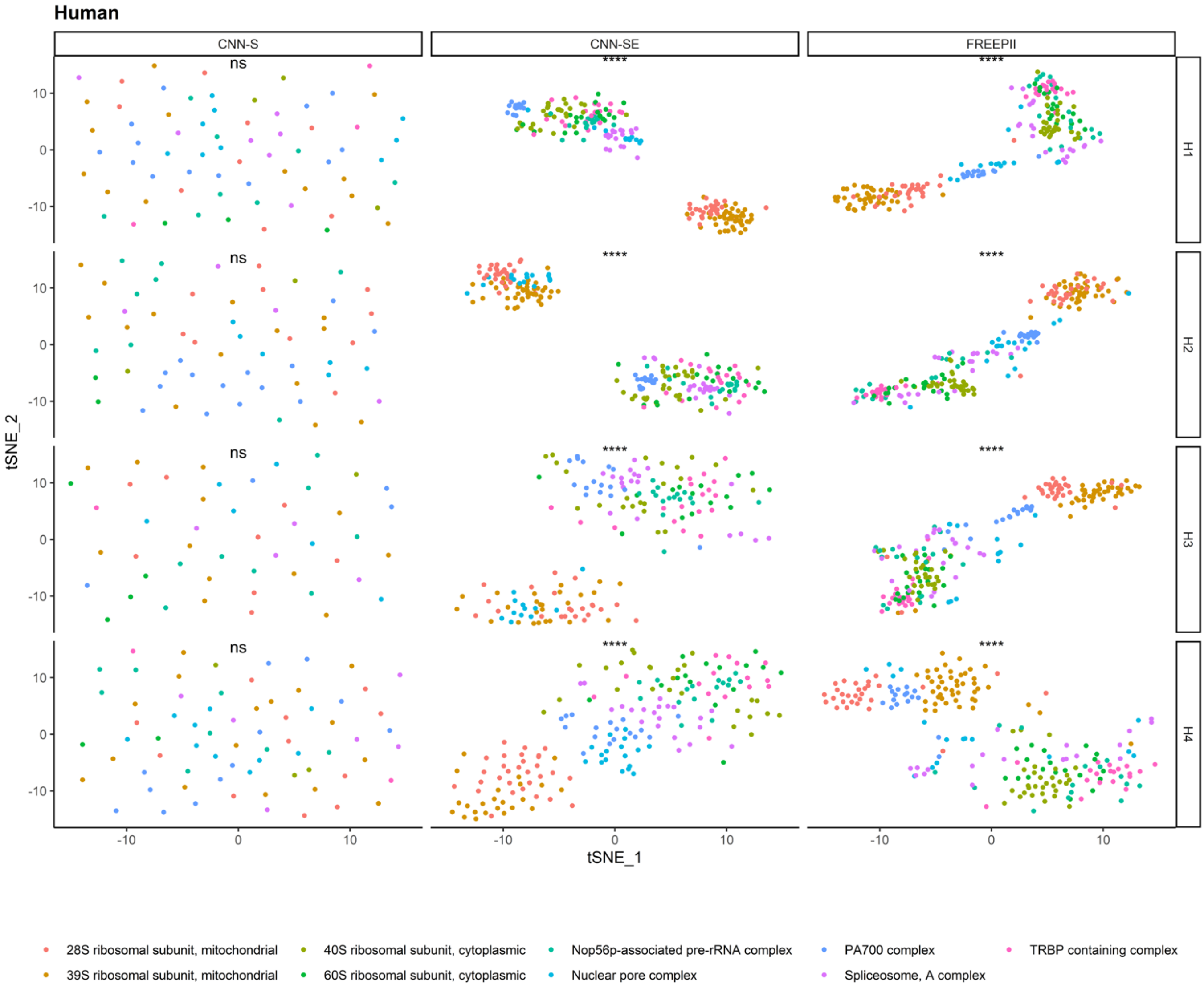

Figure S4. Visualizing feature representations of proteins learned by models via t-SNE (Human). Different colors represent labels for nine selected human protein complexes to maintain clarity in the visualization. Additionally, cosine distances between pairs of protein feature representations within and between protein complexes are calculated. The Kruskal-Wallis test is used to assess whether there is a significant difference in distance distributions between the two groups. The significance levels are denoted as follows: ns: $p > 0.05$, *: $p \leq 0.05$, **: $p \leq 0.01$, ***: $p \leq 0.001$, ****: $p \leq 0.0001$.

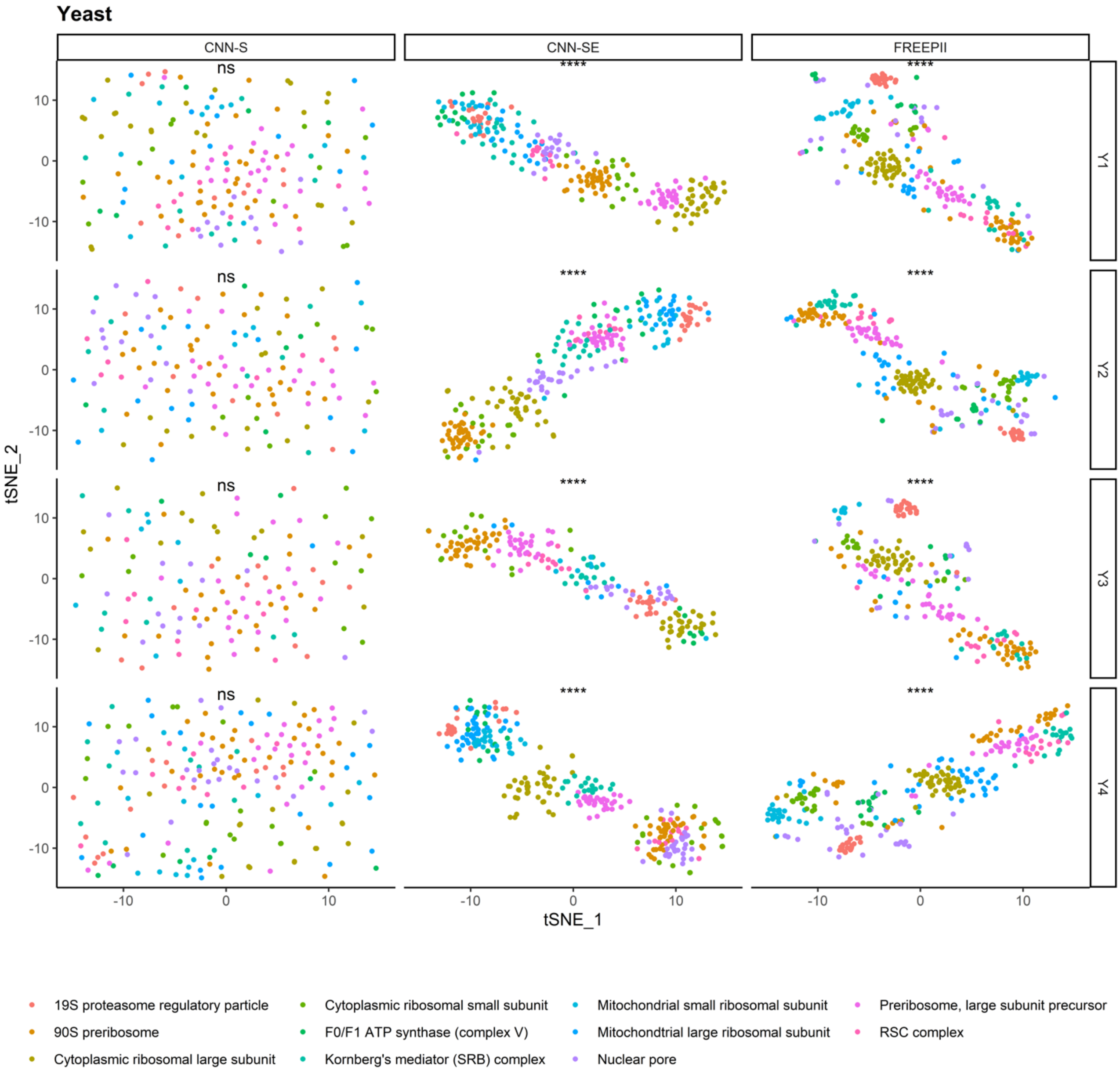

Figure S5. Visualizing feature representations of proteins learned by models via t-SNE (Yeast). Different colors correspond to labels for 11 selected yeast protein complexes, chosen to maintain clarity in the visualization. Additionally, cosine distances between pairs of protein feature representations within and between protein complexes are calculated. The Kruskal-Wallis test is used to assess whether the difference in distance distribution between the two groups is statistically significant. The significance levels are denoted as follows: ns: $p > 0.05$, *: $p \leq 0.05$, **: $p \leq 0.01$, ***: $p \leq 0.001$, ****: $p \leq 0.0001$.

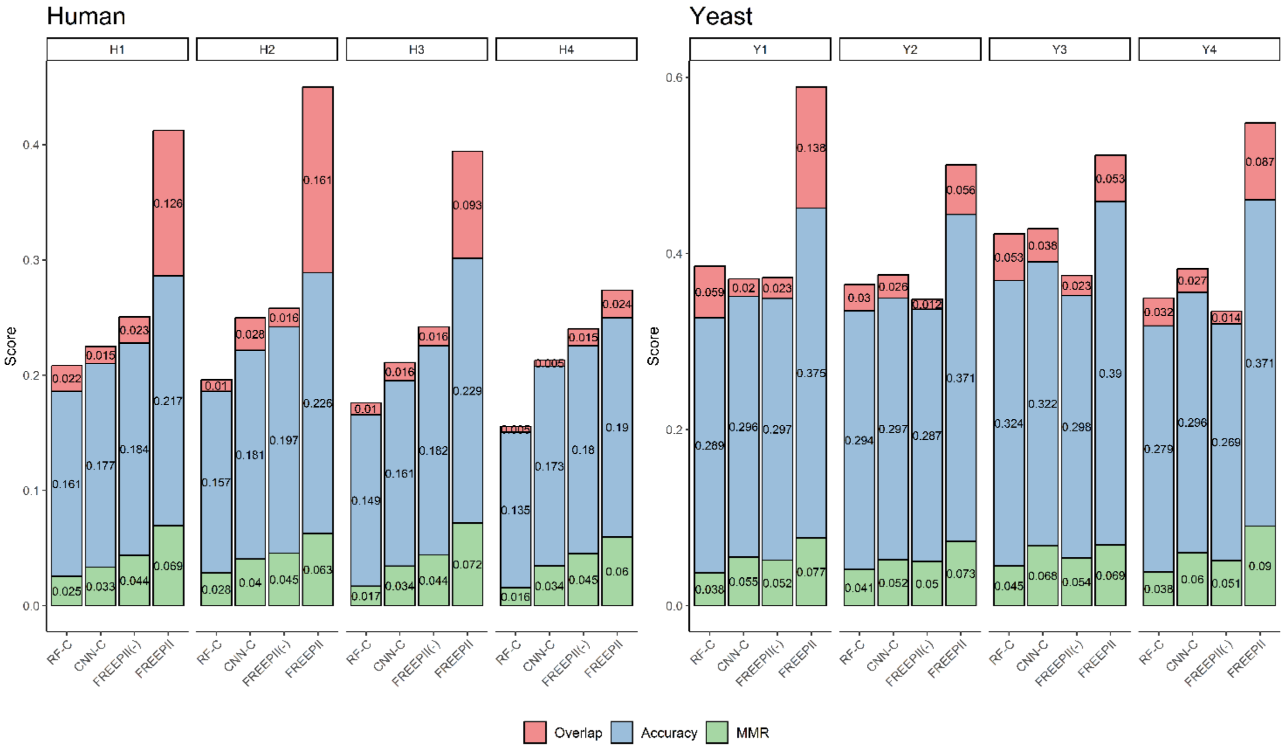

Figure S6. Composite score for clusters produced by each model in ablation study across experiments. Each model in the ablation experiment is evaluated on the composite score, which is the sum of overlap score (red), accuracy (blue), and MMR (green).

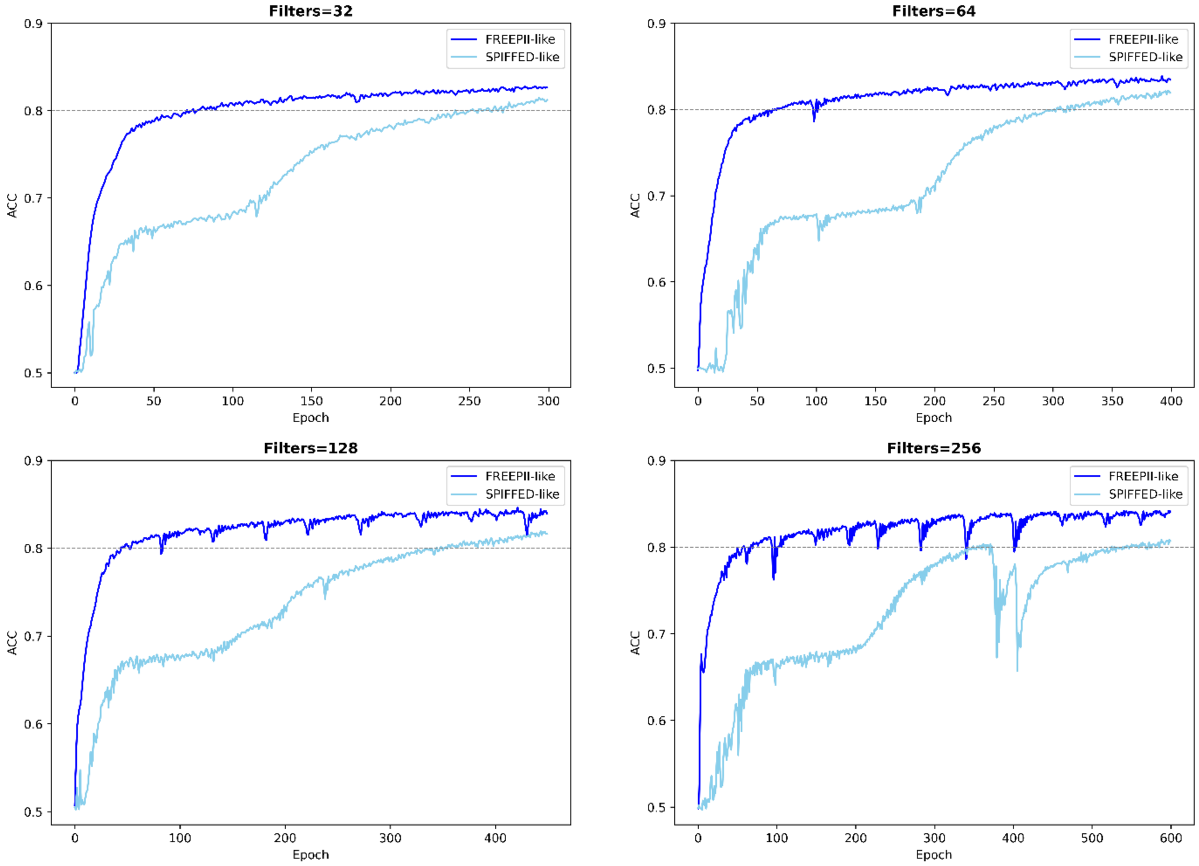

Figure S7. Training curves of the SPIFFED-like and FREEPII-like models with different numbers of filters in convolution layer. The curves illustrate the different convergence speeds of the two models. The dashed line indicates an accuracy threshold of 0.8. The time required for each model to reach this threshold is recorded and used to compare their time complexity.

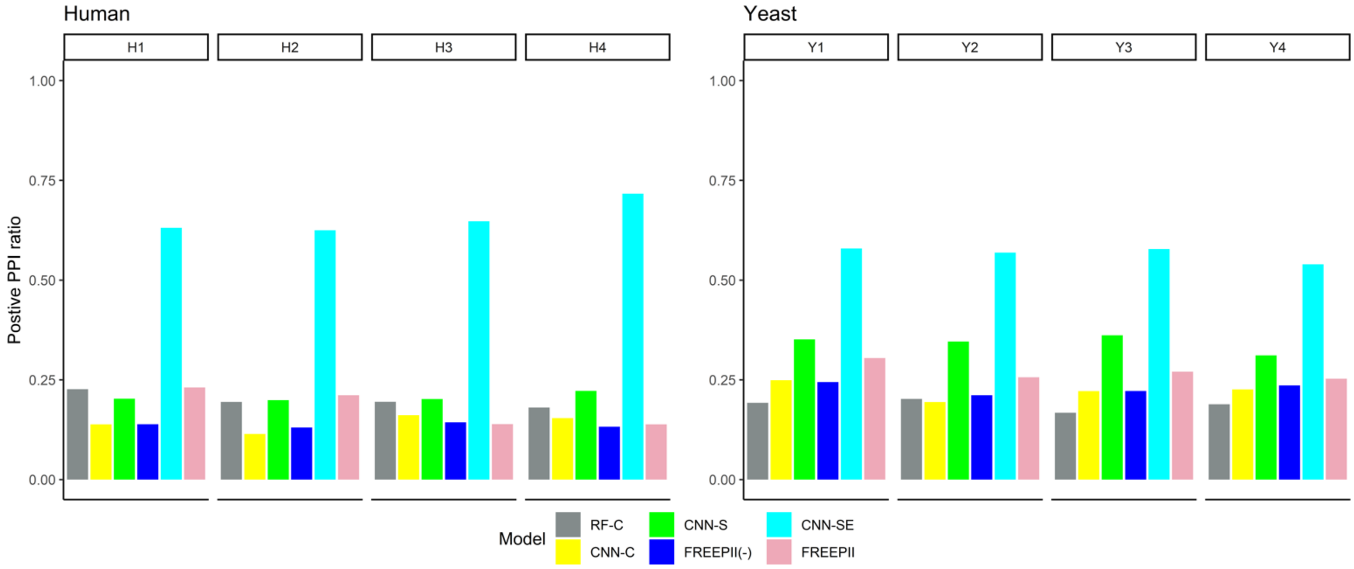

Figure S8. Proportion of positive PPIs among all predicted PPIs. The proportion of positive PPIs among all predicted PPIs is calculated, and only the classification results of CNN-SE had an unusually high proportion of positive PPIs.

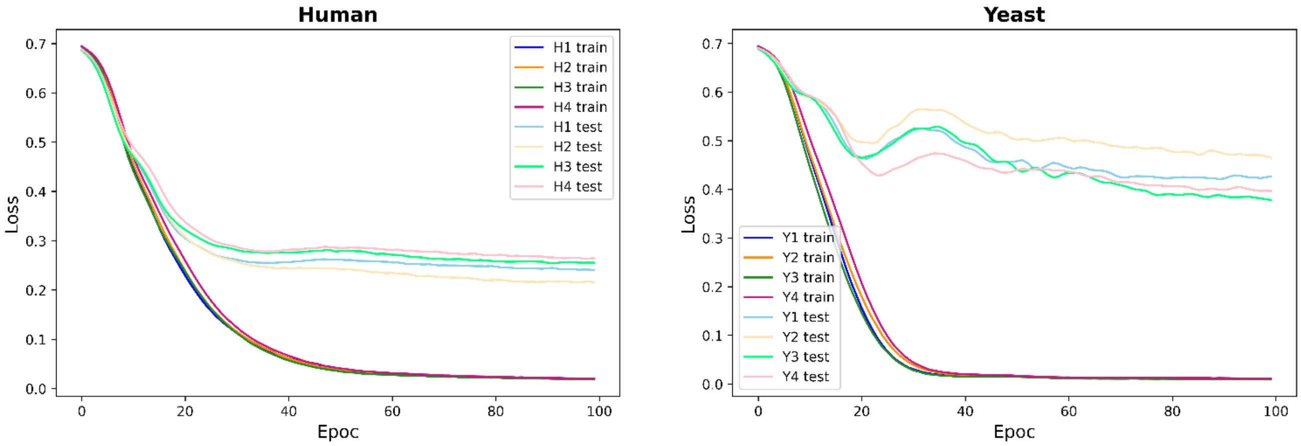

Figure S9. Learning curves of FREEPII on human and yeast datasets. Training and testing loss curves are shown to assess whether the model exhibits signs of overfitting during training.

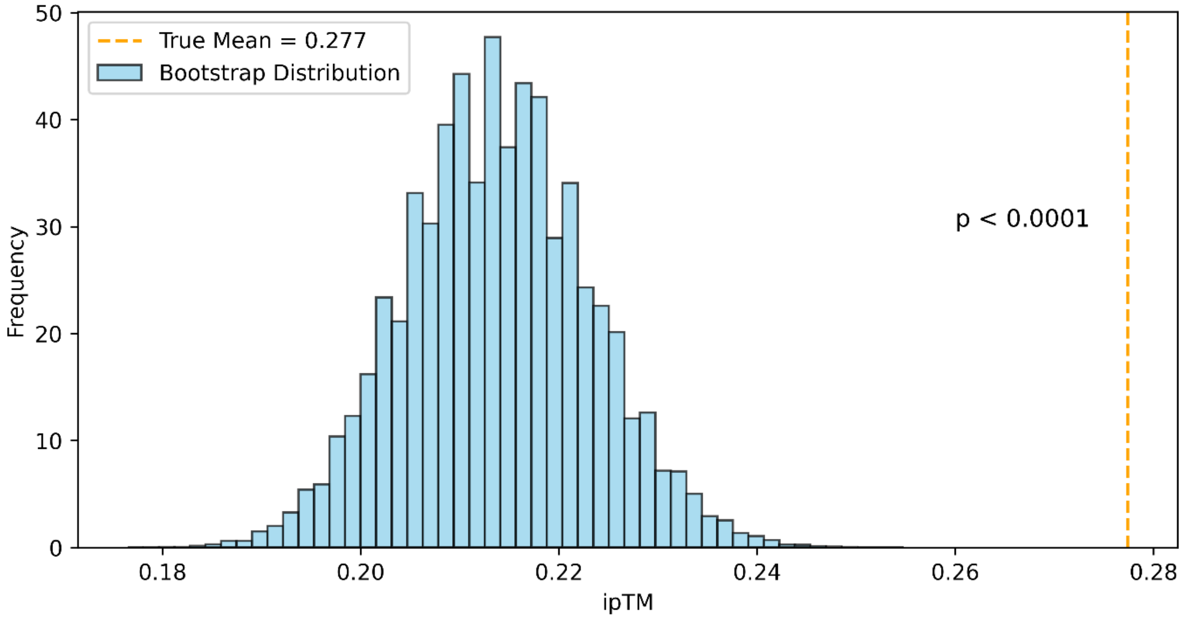

Figure S10. The average iPTM score of clusters predicted by FREEPII is significantly higher than the mean of the bootstrap-generated random distribution. The dashed line represents the mean ipTM score of the predicted clusters. The one-sample t-test was used to assess whether this mean is significantly different from that of the bootstrap distribution.
