# Supplementary material for: Complete end-to-end learning from protein feature representation to protein interactome inference
Source: Gigascience. 2025 Nov 6;14:giaf122. doi: 10.1093/gigascience/giaf122 (PMC12598752; doi:10.1093/gigascience/giaf122)

GigaScience

Complete end-to-end learning from protein feature representation to protein interactome inference

--Manuscript Draft--

**Manuscript Number:**	GIGA-D-25-00010
**Full Title:**	Complete end-to-end learning from protein feature representation to protein interactome inference
**Article Type:**	Research
**Funding Information:**	National Science and Technology Council (NSTC112-2221-E-001-021-MY3) — PhD Huai-Kuang Tsai<br>Academia Sinica (AS-GC-110-L15) — PhD Jun-Yi Leu
**Abstract:**	Co-fractionation coupled to mass spectrometry (CF-MS) is a key method to infer the protein interactome, but current analysis pipelines often suffer from biases in handcrafted features and noise interference. To address these difficulties, FREEPII, an end-to-end learning architecture based on a convolutional neural network (CNN), is introduced. FREEPII constructs the feature map coherent with model training, eliminating the need for predefined feature extractions and thus avoiding indirect bias. Unlike previous analysis methods, FREEPII prioritizes learning the feature representation of individual proteins rather than protein pairs, reducing computational complexity from O(N2) to O(N). To combat noise in CF-MS data, FREEPII uses protein sequences as additional input to extend the information available for assessing data similarity between proteins. Additionally, it uses protein embedding to transfer network-level information of protein complexes into the final feature representations, thereby rescaling the strength of interactions presented in CF-MS data. During supervise learning, the feature representations of proteins across multiple inputs can be automatically enhanced through protein embedding. FREEPII outperforms existing CF-MS analysis tools in protein-protein interactions (PPIs) classification and clustering. Visualization highlights FREEPII's advantages in representation learning, and cross-prediction experiments confirm that integrating CF-MS data with varying resolutions improves model generalization.
**Corresponding Author:**	Huai-Kuang Tsai<br>Institute of Information Science Academia Sinica<br>Taipei, 台北市 TAIWAN
**Corresponding Author Secondary Information:**	
**Corresponding Author's Institution:**	Institute of Information Science Academia Sinica
**Corresponding Author's Secondary Institution:**	
**First Author:**	Huai-Kuang Tsai
**First Author Secondary Information:**	
**Order of Authors:**	Huai-Kuang Tsai<br>Yu-Hsin Chen<br>Chien-Fu Liu<br>Jun-Yi Leu
**Order of Authors Secondary Information:**	
**Additional Information:**	
**Question**	**Response**
Are you submitting this manuscript to a special series or article collection?	No

Powered by Editorial Manager® and ProduXion Manager® from Aries Systems Corporation

**Experimental design and statistics**<br><br>Full details of the experimental design and statistical methods used should be given in the Methods section, as detailed in our Minimum Standards Reporting Checklist. Information essential to interpreting the data presented should be made available in the figure legends.<br><br>Have you included all the information requested in your manuscript?	Yes
**Resources**<br><br>A description of all resources used, including antibodies, cell lines, animals and software tools, with enough information to allow them to be uniquely identified, should be included in the Methods section. Authors are strongly encouraged to cite Research Resource Identifiers (RRIDs) for antibodies, model organisms and tools, where possible.<br><br>Have you included the information requested as detailed in our Minimum Standards Reporting Checklist?	Yes
**Availability of data and materials**<br><br>All datasets and code on which the conclusions of the paper rely must be either included in your submission or deposited in publicly available repositories (where available and ethically appropriate), referencing such data using a unique identifier in the references and in the "Availability of Data and Materials" section of your manuscript.<br><br>Have you have met the above requirement as detailed in our Minimum Standards Reporting Checklist?	Yes

GigaScience has policies and guidelines in place for the use of generative AI-writing tools such as ChatGPT. If you have used such writing tools to assist with writing the manuscript this must be declared and cited in the text. Authors should not list AI-writing tools and other AI-assisted technologies as an author or co-author and should acknowledge that they are fully responsible for text generated or refined by AI-writing tools.<p><br><br>A summary of use (particularly in the introduction or among methods) needs to be included at the end of the paper, and the outputs should also be included as a supplementary file hosted in GigaDB or other open repositories. Please read our guidelines for more information. <p><br><br>By submitting to GigaScience, you are aware of the journal's AI-writing tools policy, and if you have declared use of such tools below, you have acknowledged this where appropriate in your manuscript and have made a summary of use and outputs available. <p><br><b>AI-assisted writing tools have been used in the preparation of this manuscript?	No

Manuscript

Click here to access/download;Manuscript;FREEPII_20250109_Giga.pdf

Complete end-to-end learning from protein feature representation to protein interactome inference

Yu-Hsin Chen[1], Chien-Fu Liu[2], Jun-Yi Leu[2,*], and Huai-Kuang Tsai[1,*]

[1] Institute of Information Science, Academia Sinica, Taipei, 11529, Taiwan
[2] Institute of Molecular Biology, Academia Sinica, Taipei, 11529, Taiwan

* To whom correspondence should be addressed. Tel: +886 2 2788 3799 (Ext 1718); Fax: +886 2 2782 4814; Email: hktsai@iis.sinica.edu.tw

Correspondence may also be addressed to Jun-Yi Leu. Tel: +886 2 2789 9216; Fax: +886 2 2651 9574; Email: jleu@imb.sinica.edu.tw

Abstract

Co-fractionation coupled to mass spectrometry (CF-MS) is a key method to infer the protein interactome, but current analysis pipelines often suffer from biases in handcrafted features and noise interference. To address these difficulties, FREEPII, an end-to-end learning architecture based on a convolutional neural network (CNN), is introduced. FREEPII constructs the feature map coherent with model training, eliminating the need for predefined feature extractions and thus avoiding indirect bias. Unlike previous analysis methods, FREEPII prioritizes learning the feature representation of individual proteins rather than protein pairs, reducing computational complexity from $O(N^2)$ to $O(N)$. To combat noise in CF-MS data, FREEPII uses protein sequences as additional input to extend the information available for assessing data similarity between proteins. Additionally, it uses protein embedding to transfer network-level information of protein complexes into the final feature representations, thereby rescaling the strength of interactions presented in CF-MS data. During supervise learning, the feature representations of proteins across multiple inputs can be automatically enhanced through protein embedding. FREEPII outperforms existing CF-MS analysis tools in protein-protein interactions (PPIs) classification and clustering. Visualization highlights FREEPII's advantages in representation learning, and cross-prediction experiments confirm that integrating CF-MS data with varying resolutions improves model generalization.

Keywords: co-fractionation coupled with mass spectrometry analysis, protein interactome inference, convolutional neural network, end-to-end learning, representation learning

Background

Proteins play a central role in biological processes such as catalytic reactions, signal transduction, immune responses, and molecule transportation [1,2]. These biological activities are often executed or regulated through protein-protein interactions (PPIs), forming a complex network called the protein interactome. Deciphering the local structure of the protein interactome (protein complexes) as well as completing the global structure of the protein interactome is critical for understanding cellular functions and disease mechanisms [3–5]. Several techniques have been used to analyse transient and stable PPIs and protein complexes, including yeast-two-hybrid (Y2H) screens [6–8], affinity purification coupled to tandem mass spectrometry (AP-MS) [9,10], and protein co-fractionation coupled to mass spectrometry (CF-MS) [11–13].

To date, the results of Y2H and AP-MS analysis have been used to establish approximately 53,000 [7] and over 56,000 [5] protein-protein interactions in human cells, respectively. Nonetheless, both systems have their limitations. The Y2H screen assay can only detect binary interactions in one experiment and requires the target proteins to be expressed in yeast cells, which may affect PPIs due to inappropriate modifications or incorrect localizations of proteins. Additionally, Y2H screens cannot determine PPIs in a specific cellular state. Instead, AP-MS can obtain protein interactions under specific conditions and map multiple protein interactions in parallel. However, AP-MS often requires specific antibodies or genetic engineering, which may alter protein structure and interaction sites. Furthermore, AP-MS can only detect stable interactions and will miss unstable or transient interactions.

CF-MS is an alternative approach for the large-scale detection of protein interactions. It requires no exogenous introduction of genetic or affinity purification materials and allows the examination of thousands of protein interactions in a near-physiological context. CF-MS analysis involves correlation measurements of co-elution profiles and graph partitioning algorithms for *de novo* PPI and protein complex inference [11]. However, the sensitivity of this correlation-based protein interaction assignment is limited by the "feature extraction" method and the noise present in the CF-MS data itself. Current CF-MS analysis methods often require hand-crafted features for classifier training [14–17], resulting in information compression and the introduction of bias. Our previous research proposed an end-to-end learning model based on convolutional neural network (CNN) architecture, named SPIFFED [18], to address the limitations of hand-crafted features. SPIFFED outperforms the state-of-the-art hand-crafted feature-based tool, EPIC [16], across all evaluation metrics for the PPI classification task, demonstrating the superiority of CNN in automated feature extraction. Despite its advancements, SPIFFED still faces challenges in mitigating noise-induced false positives and false negatives in co-elution profiles.

Here, we propose a method called Feature Representation Enhancement End-to-end Protein Interaction Inference (FREEPII), an end-to-end learning method encompassing autonomous feature extraction and feature representation enhancement for PPIs and protein complexes inference. FREEPII employs a CNN as its main architecture, so the construction of feature maps can be coherent with model training. Unlike SPIFFED, which focuses on the feature representation of protein pairs, FREEPII emphasizes learning the feature representation of individual proteins, reducing computational complexity from $2*N*(N\text{-}1)*M$ to $N*M$ (N: number of proteins, M: fractionation number of CF -MS data). To reduce the interference of noise existing in CF-MS data, FREEPII uses the second input, protein sequences, to extend the information available for calculating data similarity between proteins. In addition, it uses protein embedding to transfer the network-level information from labels into the final feature representations to rescale the strength of interactions present in CF-MS data. Furthermore, the feature representation of proteins can be automatically enhanced through protein embedding during supervised learning [19,20]. Our results show that FREEPII substantially outperforms EPIC and SPIFFED, the two well-established CF-MS analysis tools, in both PPI classification and clustering. We reveal the advantages of FREEPII in representation learning and classification judgment through visualization. Through cross prediction, we demonstrated that combining CF-MS data with different resolutions for model training can significantly improve the generality of CNN for PPI classification in different experiments. The source code of this study is freely available at: https://github.com/qqpigass/FREEPII.

Results

Analysis pipeline of FREEPII

Figure 1 shows the comprehensive analysis workflow of FREEPII. FREEPII takes CF-MS data and protein sequences as inputs. To convert the amino acids of the protein sequence into numeric representation, each protein sequence is flattened into one-dimensional FCGR (see Methods). Before feature extraction, FREEPII combines the protein sequence matrix and protein embedding matrix into a single matrix, which is then concatenated with the CF-MS data matrix. Subsequently, FREEPII conducts feature extraction from the concatenated matrix through a CNN layer, generating a matrix of protein feature representations. These learned representations can be used to form representations of protein pairs via the pairing indexes for downstream classification task and clustering analysis. Throughout backpropagation, the feature extraction process undergoes automatic optimization. Furthermore, network-level information present in the labels can be transferred to the protein feature representations through protein embedding.

FREEPII achieves the best performance in PPI classification

To evaluate the performance of FREEPII on PPI classification, we compared it with two models, EPIC and SPIFFED (Figure 2A). Across all metrics evaluated for both human and yeast datasets, FREEPII demonstrates superior performance compared to EPIC and SPIFFED. Specifically, on the testing set, FREEPII achieves higher average sensitivity, specificity, MCC, and AUC of ROC (that are 0.150/0.093, 0.103/0.039, 0.250/0.135 and 0.108/0.063 in the result of human/yeast, respectively) than those of SPIFFED.

FREEPII incorporates three key design elements: a novel feature extraction method based on CNN, the consideration of intrinsic protein properties such as protein sequences, and the integration of network-level information through protein embedding. To assess the effectiveness of FREEPII's components, we initially compare the performance of the CNN model with that of the RF in PPI classification using only CF-MS data as input. Figure 2B illustrates that the CNN model significantly outperforms RF in classifying human dataset and slightly outperforms it in yeast dataset. This demonstrates the CNN's superiority in leveraging all available information for classification. Moreover, incorporating protein sequences as additional input in our model leads to further improvements in its ability to identify positive PPIs (the increased sensitivity scores depicted in Figure 2B) in both datasets. Consequently, the performance of the CNN model with protein sequences as additional input surpasses that of the CNN model using merely CF-MS data as input, highlighting the utility of protein sequences in reducing false negative classification errors. Subsequently, we assess the impact of the protein embedding. As shown in Figure 2B and Figure S3, the CNN models with protein embedding not only enhance their abilities to identify positive PPIs, but also enhance their abilities to identify negative PPIs (the evaluation scores of both sensitivity and specificity are increased). Such results reflect the advantages of FREEPII in combining CF-MS data together with protein sequences for learning and guiding the network-level information from labels into the protein feature representations through protein embedding, thereby achieving the highest performance among all evaluation methods in PPI classification.

The discriminative power of FREEPII from both CF-MS data and protein sequences

To delve deeper into the roles of CF-MS data and protein sequences in FREEPII's prediction of PPIs, we evaluated their respective contributions for each PPI by estimating the average difference in intensity from the saliency map (see Methods). As shown in Figure 3A, FREEPII utilizes information from both CF-MS data and protein sequences nearly equally for predicting most PPIs, suggesting their equal importance in FREEPII's classification process. However, for a small subset of PPIs, FREEPII relies more heavily on CF-MS information than on protein sequences to determine their classes, with very few PPIs being primarily classified based on protein sequence information. As depicted in Figure 3B, when FREEPII learns more towards CF-MS information, it predominantly focuses on the first few fractions of CF-MS data. Such results may be indicative of proteins eluted early in the CF-MS data due to heightened volume in the interaction state. Conversely, when the model predominantly relies on protein sequence information to classify PPIs, it minimally utilizes CF-MS information. This observation suggests that certain protein interaction relationships are already discernible within the protein sequences themselves.

Protein embedding transfer network-level information in labels to protein feature representation

To validate the capacity of protein embedding in incorporating network-level information from labels into protein feature representation, we employed *t*-Distributed Stochastic Neighbor Embedding (t-SNE) to condense the feature representation and visualized it with protein complexes as labels (Figure 3C, Figure S4, Figure S5). To maintain clarity in interpretation, we only display the results of 9 human protein complexes and 11 yeast protein complexes. As shown in Figure 3C, the feature representations of proteins learned from the CNN model with only protein sequences as input (CNN-S) fail to reflect the correlation of protein complex labels. However,

with the incorporation of protein embedding (CNN-SE), the learned feature representations can be grouped based on protein complex labels, indicating that the successful transfer of network-level information from labels to the final feature representation through protein embedding. The protein feature representation learned by FREEPII combines the protein interaction information present in the CF-MS data and protein embedding, and its groups are better separated than the results of CNN-SE in both dimensions of t-SNE (Figure S4, Figure S5). For example, in human datasets H3 and H4, FREEPII clearly clusters members of the 28S ribosomal subunit (mitochondrial) and the 39S ribosomal subunit (mitochondrial), whereas CNN-SE results in more scattered representations with less distinct group boundaries. Similarly, for yeast datasets, the Mitochondrial large ribosomal subunit representations learned by CNN-SE are more dispersed and overlap significantly with those of other protein complexes, in contrast to the more well-defined clusters produced by FREEPII.

We also computed cosine distances between pairs of feature representations within and between protein complexes. As illustrated in Figure 3C, the feature representations learned from CNN-S exhibit minimal discrepancy between pairwise distances within protein complexes and those between different protein complexes. Conversely, in the results of CNN-SE and FREEPII, significant differences emerge in the distances between pairwise feature representations within versus between protein complexes. This again demonstrates that network-level information from labels is effectively integrated into the feature representations through protein embedding.

FREEPII achieves the best performance in cluster quality evaluation

Next, we evaluate the efficacy of FREEPII in generating quality clusters. Initially, we employ the composite score (see Methods) to assess the structural compositional similarity between the resulting clusters and the reference protein complex dataset from various perspectives. Given FREEPII's superior accuracy in PPI classification, it achieves the highest composite scores for both human and yeast datasets compared to EPIC, SPIFFED, and other models in the ablation study (Figure 4A, Figure S6).

Since the reference protein complex dataset encompasses a limited number of human-curated protein complexes, we further used the GOGO scoring metric [21], which does not rely on a reference dataset, to assess the functional similarity between proteins within the same cluster (see Methods). As shown in Figure 4B, Table S1 and Table S2, clusters generated by FREEPII consistently attain the highest GOGO scores across almost all human datasets, irrespective of the GO ontology considered. For yeast datasets, FREEPII outperforms the two tools and other models in all experiments within the BP ontology, and demonstrates comparable performance in other two ontologies.

Lastly, we assess our results using protein co-localization information (see Methods). Across most human and yeast datasets, clusters generated by FREEPII also exhibit the highest co-localization scores (Figure 4C and Table S3, Table S4). Together, these findings indicate that FREEPII has a higher propensity for grouping protein pairs with similar functions and close spatial proximity. It is worth noting that when using multi-inputs without protein embedding (FREEPII(-)), the performance metrics (the composite score, GOGO score and co-localization score) still surpass those models using solely on co-eluted data in the ablation studies (Figure S6, Table S2 and S4). This underscores the potential of leveraging protein sequences to enhance the quality of generated clusters.

Co-training greatly improves the prediction generality of FREEPII(-)

Given the varied resolutions of currently available CF-MS data across different species, it is of interest to see whether prediction performance can be improved by considering datasets with diverse numbers of fractionations and species. As the dimension of the embedding is fixed based on the input size and retains information about the specific label set after training, for comparison, here we use FREEPII without protein embedding (FREEPII(-)) to allow various numbers of proteins and different label sets for co-training. We conducted cross-prediction across CF-MS datasets with different resolutions and species, i.e. use each model trained on one or multiple datasets to predict on others.

Figure 5A presents the performance comparison of cross-prediction. The RF model (RF-C) outperformed CNN-C in almost all human and yeast predictions than CNN-C when trained with a single CF-MS dataset (H1 or Y1). When trained on yeast dataset Y1, The CNN-C performed better than RF-C in predicting other yeast data but performed poorly in predicting human data. Conversely, when trained on human dataset H1, CNN-C exhibited stable performance for both human and yeast datasets. Such results indicate that due to the simpler input, the CNN model trained on the yeast data (27 fractions) may not be able to effectively extract useful features from

the more complex human data (57-61 fractions); on the contrary, the CNN model trained using the human data is still able to cope with the feature processing of yeast data. Using protein sequences failed to improve the performance of FREEPII(-) in cross-prediction. These results indicate that when a CNN-based model is trained using a single CF-MS dataset, regardless of whether protein sequences are used, its performance is significantly influenced by the resolution of the data itself, whereas RF trained with artificially defined features is less affected.

However, as more and more CF-MS data with different resolutions were used for co-training, CNN-C gradually outperformed RF-C in predicting both humans and yeast datasets. Further, by considering protein sequences, FREEPII(-) achieved the best performance. Figure 5B shows that, with exposure to more data, the prediction abilities of CNN-based models could be substantially improved (The MCC of RF-C increases by up to 0.05, while the MCC of CNN-C and FREEPII(-) increases by up to 0.15 and 0.17, respectively), while the prediction ability of RF-based model only improve marginally. These results suggest that RF trained using artificially defined features has limited improvement in prediction regardless of the number of CF-MS datasets used for training. In contrast, CNN-based models co-trained with different CF-MS datasets can enhance their feature extraction process and achieve prediction accuracies that surpass the upper limit of models trained on single CF-MS data. Furthermore, considering protein sequences can further enhance the prediction performance of the co-trained CNN-based models.

Discussion

In this study, we propose a novel architecture to infer PPIs and protein complexes by simultaneously considering muti-inputs and protein embedding using a CNN-based feature extraction method. Across all classification performance metrics for human and yeast datasets, FREEPII outperforms two well-established tools, EPIC and SPIIFED (Figure 2A). This demonstrates that the proposed method significantly reduces false negative and false positive errors. Additionally, we show that FREEPII's discriminative power is learned from both the CF-MS data and protein sequences (Figure 3A, 3B), and effectively incorporates the network-level concept of protein complexes present in the labels into the protein feature representation (Figure 3C), resulting in more discriminative final representation. FREEPII's optimized approach to PPI classification and representation learning allows it to achieve the highest scores in all clustering performance evaluation indicators (Figure 4), showing its comprehensive performances. Overall, we demonstrate that FREEPII is a conceptually innovative and empirically supported method that significantly outperforms existing CF-MS analytical methods.

In Figure 2B, when only CF-MS data are considered, the classification performance of CNN surpasses that of RF for the human datasets and is only slightly better for the yeast datasets. Since the human CF-MS datasets all have a higher second dimension (higher number of fractionations) than the yeast CF-MS dataset, the information compression of the human CF-MS data will be more significant than yeast CF-MS data when extracting hand-crafted features. Consequently, the superior performance of CNN for the human datasets compared to RF reflects CNN's advantage in leveraging comprehensive information for classification and enhancing the automatic feature extraction process through training. Additionally, Figure 2B demonstrates that using protein sequences as additional input of FREEPII can significantly reduce the false negative errors, which is consistent with previous studies showing that multi-inputs can improve model performance especially in sensitivity [22,23]. In fact, FREEPII supports incorporating arbitrary sequence-based representations, such as gene expression matrices or protein structure embedding obtained from pre-trained model [24,25]. In the study of Singh and colleagues [26], they also proved that integrating network-level information improves PPIs predictive performance. However, their method requires additional operations on the adjacency matrix and does not improve as the model training. In contrast, in FREEPII, network-level information is automatically passed through embedding, which can be trained coherent with model learning. The embedding can also serve as corrections to the additional input. If any information is missing in the additional input, the embedding values could impute these missing values [27], thereby achieving data completion. Therefore, FREEPII is a complete end-to-end coherent architecture, encompassing data integrity, feature extraction and representation enhancement, for PPI classification and PPI clustering.

Although embedding is powerful to enhance input feature representations, there are some potential caveats. We found that the CNN model with only protein sequences as input and incorporating protein embedding yields unreasonably high positive PPIs when classifying all PPIs (including training PPIs, testing PPIs and experimental PPIs) (Figure S7), showing imbalanced prediction. This is because the embedding vectors of proteins are trained through backpropagation of the PPI classification loss, but the PPIs in the training set do not

contain all the proteins in the experiment, causing the embedding vectors of some proteins remain in their initial state after training. When the input value is close to the embedding, such as the FCGR of the protein sequence, it is severely affected by untrained embedding, interfering with the model's judgment of PPIs composed of proteins lacking in training PPIs set. FREEPII avoids this problem by concatenating CF-MS data and protein sequence FCGR rather than adding them, ensuring that the values of the CF-MS data are not interfered by untrained embedding vectors. Additionally, the numerical range of the CF-MS data is larger than that of embedding and FCGR, so its impact on model training and prediction is greater. As shown in Figure S7, when FREEPII is applied to predict all PPIs, it produces a proportion of positive PPIs comparable to that of the CNN with only CF-MS data as input (CNN-C), demonstrate that FREEPII is unaffected by untrained embedding.

We further demonstrate that when FREEPII is co-trained on multiple CF-MS datasets with various fractionation numbers, it offers more general prediction than when trained on a single CF-MS dataset alone. This principle is similar to training CNNs for image classification, whereas various image augmentations enhance the versatility of CNN predictions [28]. Our results show that co-training a CNN-based model using CF-MS datasets with different fractionation numbers improves PPI prediction accuracy. Furthermore, incorporating additional inputs, such as protein sequences, further enhances the prediction accuracy of the co-trained CNN, particularly in identifying positive PPIs. This is crucial as fewer positive PPIs are known compared to negative PPIs, making accurate identification of positive PPIs more valuable for further research.

Conclusions

In summary, we demonstrated that FREEPII can utilize information from both CF-MS data and protein sequences to achieve high accuracy in PPI classification and good cluster quality. By incorporating protein embedding, FREEPII transfers network-level information of protein complexes from labels and learns a discriminative protein feature representation with CNN, optimizing the overall training process and model performance. Co-training FREEPII on CF-MS datasets with different fractionation numbers significantly improves the generality of FREEPII for PPI classification across different experiments.

Methods

CF-MS dataset curation and data pre-processing

The human CF-MS datasets (PXD002892, PXD014820, and PXD015406) were downloaded from Zenodo (doi: 10.5281/zenodo.4106578), where all uploaded data were reanalysed by Skinnider and Foster using MaxQuant [14]. Among the various files corresponding to different protein quantification strategies provided by the authors, we selected those containing iBAQ intensity of chromatograms for further analysis. The yeast (*Saccharomyces cerevisiae*) CF-MS dataset (PXD031967) was curated by ourselves, with the detailed experimental processes described in our previous publication [29]. The number of proteins, the number of fractions, and the fractionation methods for all datasets are listed in Table 1. Protein overlap between experiments on the same species is shown in Figure S1. For handling missing values, we replaced them with zeros and then removed rows containing only zero values. Subsequently, we conducted normalization to ensure the sum of each CF-MS profile equalled 1. The number of genes in each CF-MS dataset is also listed in Table 1. Given that the number of fractions varies across different CF-MS datasets, each CF-MS dataset is padded to a fraction size equal to 200 before fed into the model.

Protein complex collection and protein pairs labelling

The human protein complex dataset was downloaded from the CORUM database [30], while the yeast protein complex dataset was extracted from the list provided by Swamy et al. [29]. In total, 3614 and 575 protein complexes were documented in human and yeast, respectively. We filtered out protein complexes consisting of fewer than three genes, resulting in 2277 human and 317 yeast protein complexes. The number of proteins present in the known protein complexes for each CF-MS dataset is shown in Table 1.

Protein pairs within the same protein complex are labelled as ‘positive PPIs’ and protein pairs that exist between different protein complexes are labelled as ‘negative PPIs’. However, positive PPIs are reclassified as negative if they lack any co-eluting characteristic (where signal multiplication for the same fraction is greater than 0.01).

Protein pairs in the CF-MS data are categorized as ‘experimental PPIs’ if they neither fall into the positive or negative PPI categories. Only positive PPIs and negative PPIs are used for model training and evaluation.

Protein sequence collection and numerical representation

Human and yeast protein sequences were retrieved from UniProt database [31] and subsequently converted into frequency matrix chaos game representation (FCGR) [32,33] using R package ‘kaos’. We set the resolution to 16 and scaling factor to 0.863271 to prevent the overlap of attractors [34,35]. The frequency matrix of each of N proteins was then reshaped into dimension 1*256*1 and concatenated in the first dimension to form a matrix with dimension N*256*1 as the input of the model.

Model architecture

FREEPII consists of an embedding layer, a CNN layer, and three fully connected layers, as shown in Figure S2. The embedding layer generates protein embeddings, which are directly added to the FCGR matrix. This combined matrix is then concatenated with the CF-MS matrix, forming the input for FREEPII. The input is subsequently transformed into a feature map via the CNN layer, with the original input being reintroduced into each filter channel of the feature map. Features corresponding to paired proteins in the feature map are extracted using the provided pairing indexes, followed by the subtraction of paired features. A similar process is applied to the input to get another matrix of feature differences. These two difference matrixes, derived from the input and feature map, are concatenated along the second dimension and the final dimension is flattened to form a two-dimensional matrix. This matrix is then passed through three linear layers to generate the final prediction scores for the protein pairs. Detailed parameter settings are available in the code: https://github.com/qqpigass/FREEPII.

Model training and evaluation on PPIs

During model training, subsets of positive and negative PPIs were used, maintaining a ratio of 1:1, and five-fold cross-validation was applied to obtain the average performance of the model. To classify predicted interactions as positive or negative, a hard threshold of 0.5 was set. Interactions with predicted scores less than or equal to 0.5 were defined as negative PPIs, while those above 0.5 were considered as positive PPIs. Four classification evaluation metrics were used, including Sensitivity, Specificity, Matthews Correlation Coefficient (MCC), and Area Under Curve for Receiver-Operator Characteristic (AUC of ROC), defined as follows:

$$Sensitivity = \frac{TP}{TP + FN}$$

$$Specificity = \frac{TN}{TN + FP}$$

$$MCC = \frac{TP \times TN - FP \times FN}{\sqrt{(TP + FP)(TP + FN)(TN + FP)(TN + FN)}}$$

$$AUC\ of\ ROC = 1 - Specificity\ at\ various\ threshold\ values$$

Comparison with other CF-MS analysis tools

We compare the performances of FREEPII in both PPI classification and clustering evaluation with two existing CF-MS analysis tools, EPIC and SPIFFED. For EPIC, we used its default feature extraction metrics, namely Mutual Information, Bayes Correlation, Euclidean Distance, Jaccard Score, Apex Score, to generate features of PPIs for model training. We perform a five-fold cross-validation under the conditions of data balance and a training-test ratio of 70:30 [18]. For a fair comparison, our clustering algorithm is used to generate clusters from the outputs of EPIC and SPIFFED.

Ablation study on model architecture

We conducted an ablation study to gain deeper insights into the impact of design components, including feature extraction, consideration of protein sequences, and network-level information. In this study, we employed a Random Forest (RF) model using CF-MS data as input (denoted as RF-C in this study) as the baseline model for PPI classification tasks. The depth of RF was set to 1000, and seven features-- including distance correlation,

weighted cross correlation (WCC), mutual information (MI), cosine similarity, Pearson and Spearman correlation, and Kendall rank correlation-- were computed as the features of paired CF-MS profiles. These feature combinations enabled the RF model to achieve the best classification performance among those studied by Skinnider and Foster [14]. The structures of CNN-based models for ablation study are shown in Figure S2.

Visualization of feature representations of proteins labelled by protein complexes

The feature map of CF-MS data (*N*M*F*, where *N* represents the number of proteins, *M* denotes the dimension along fractions, and *F* indicates the number of filters) was extracted, and the dimension of filters was flattened to form a new matrix with dimension *N*M'* (where *M'* represents the product of the dimension along fractions and the number of filters). Each row in this matrix represents a feature representation of a protein. Subsequently, we merged the protein feature representations with the names of the protein complexes to which they belong. To ensure visualization clarity, we selected 9 human protein complexes (28S ribosomal subunit, mitochondrial; 39S ribosomal subunit, mitochondrial; 40S ribosomal subunit, cytoplasmic; 60S ribosomal subunit, cytoplasmic; Nop56p-associated pre-rRNA complex; Nuclear pore complex; PA700 complex; Spliceosome, A complex; TRBP containing complex) and 11 yeast protein complexes (19S proteasome regulatory particle; 90S preribosome; Cytoplasmic ribosomal large subunit; Cytoplasmic ribosomal small subunit; F0/F1 ATP synthase (complex V); Kornberg's mediator (SRB) complex; Mitochondrial small ribosomal subunit; Mitochondrial large ribosomal subunit; Nuclear pore; Preribosome, large subunit precursor; RSC complex) for labelling. Duplicate proteins were filtered out before merging with the feature representation matrix. The labelled feature representation matrix was then dimensionally reduced to two dimensions via t-SNE for visualization.

Visualize feature hotspots for classifying each PPI by computing saliency maps

To assess the contributions of CF-MS data and protein sequences in FREEPII's prediction of PPIs, we employed the saliency map [36] to visualize the feature hotspots for each PPI classification. Based on the average intensity difference of the CF-MS data and protein sequences inputs, we categorized the classified PPIs into three groups: if the average intensity of the CF-MS region exceeds that of the protein sequence region by more than 0.1, the classification is labelled as CF-MS > SEQ; if it is less than -0.1, it is labelled as CF-MS < SEQ; otherwise, it is classified as CF-MS ~ SEQ. The final representation of the saliency map for each PPI category is formed by superimposing the values of the saliency map calculated for each PPI and normalizing these values to range between 0 and 1.

Generating clusters using predicted PPI scores

The pairing indexes (edges) and prediction scores (weights) are used to construct the adjacency matrix 'A'. Proteins (nodes) without any neighbours are removed. The adjacency matrix 'A' is then processed through the Markov cluster algorithm (MCL) [37,38] with the expansion and inflation parameters set to 2, iterating three times to obtain the matrix 'A_'. To consider the topological properties within the adjacency matrix, the topological overlap matrix (TOM) [39] 'I_' is calculated according to the following formula:

$$w_{ij} = \frac{l_{ij} + a_{ij}}{min\{k_i, k_j\} + 1 - a_{ij}},$$

where w_{ij} is the new weight of edge between node 'i' and node 'j', a_{ij} is the weight of edge between node 'i' and node 'j' on the 2.5th power of 'A', $l_{ij} = \sum_u a_{iu} a_{uj}$,, and $k_i = \sum_u a_{iu}$ is the node connectivity. Then matrix 'A_' and matrix 'I_' are then combined in proportions of weights 0.3 and 0.7, and weights 0.1 and 0.9, respectively, to form two importance matrices. Cosine distance matrices are calculated from these two matrices, followed by Ward hierarchical clustering. The dynamic cut-tree algorithm of the Python function 'cutreHybrid' is used to obtain clusters, with 'minClusterSize' set to 3 and 'deepSplit' set to 3 ('deepSplit' should be set to 1 or 2 for relatively small amounts of data).

The clusters obtained from the two matrices are then combined to form a set of clusters with overlapping members. To prevent the generation of unreasonably large clusters, we limit the size of the clusters to less than 100. If any cluster exceeds this limit, the above splitting steps are repeated ensure compliance. For highly overlapping clusters, an iterative merging step is performed. The merge threshold is set to 0.25, where the denominator is the product of the sizes of two clusters, and the numerator is the square of the amount of overlap between the two clusters.

Gene function annotations and semantic similarity measurement of GO-terms

We retrieved the semantics and relationships between GO terms from the GO Consortium released on November 4, 2022. To measure the semantic similarity between GO terms of proteins within the same complex, GOGO algorithm is used [21,40]. For a GO term '*t*', the semantic contribution weight is calculated according to the link type and the number of child nodes, considering the semantic contribution of ancestors in GO directed acyclic graph (DAG) to '*t*':

$$W_e = \frac{1}{(c + nc(t))} + d,$$

where '*nc(t)*' is the total number of child nodes for GO term '*t*'. The constant parameter '*c*' is set to 0.67 in GOGO to ensure $0 < W_e \leqslant 1$. The constant parameter '*d*' is assigned values of 0.4 and 0.3 for the '*is-a*' and '*part-of*' relationships, respectively. For each term in DAG_t, it has a semantic contribution to the target term '*t*', defined as the *S*-value:

$$\begin{cases} S_t(self) = 1 \\ S_t(other) = max\{W_e \times S_t(other) | other \in children(t)\} \end{cases}$$

The semantic value of GO term '*t*' is the summation of *S*-values in DAG_t:

$$SV(t) = \sum_{i \in t, ancestors(t)} S_t(i)$$

Given another GO term '*k*', the semantic similarity between two GO terms is defined as:

$$S_{GO}(t,k) = \frac{\sum_{i \in (ancestors(t) \cap ancestors(k))} S_t(i) + S_k(i)}{SV(t) + SV(k)}.$$

To calculate the semantic similarity between a gene G_1 with *m* GO terms $go_{11}, go_{12}, \ldots go_{1m}$ and a single GO term '*t*', the equation is as follows:

$$Sim(t, G_1) = \max_{1 \le i \le m} \left(S_{GO}(t, go_{1i})\right).$$

Given another gene G_2 with *n* GO terms $go_{21}, go_{22}, \ldots go_{2n}$, the functional similarity between G_1 and G_2 is:

$$Sim(G_1, G_2) = \frac{\sum_{1 \le i \le m} Sim(go_{1i}, G_2) + \sum_{1 \le j \le n} Sim(go_{2j}, G_1)}{m + n}.$$

For a protein complex, the average of the pairwise functional similarities between all genes comprising the complex is calculated.

Co-localization within protein complexes

To assess the similarity of protein localizations within a complex, we use the co-localization score as defined in the study [41]. The subcellular locations of proteins are download from UniProt database [31]. The co-localization score of a protein complex is calculated as follows:

$$Score_{co-localization} = \frac{max_i l_i}{|C|},$$

where l_i is the number of proteins of complex *C* assigned to the localization group '*i*' and |*C*| is the number of proteins in the complex *C* with localization assignments. This score provides the maximum fraction of proteins within the complex that share the same localization, divided by the total number of proteins in that complex with known localizations. This metric ensures that the higher the score, the more co-localized the proteins within a complex are, indicating better functional coherence.

Structure similarity between predicted clusters and reference protein complex dataset

To evaluate the structural compositional similarity between predicted clusters and a reference protein complex dataset, we use the composite score, as described in the literature [16,42]. The composite score is the sum of three components: Overlap, Accuracy, and Maximum Matching Ratio (MMR) [42]. Overlap is defined as the percentage of predicted clusters that have an overlap score larger than 0.25 with any reference complex. The overlap score is calculated as the square of the number of overlapping proteins between the predicted cluster and

the reference complex divided by the product of their sizes. Accuracy is the geometric mean of Sensitivity and PPV, where Sensitivity and PPV are calculated by the following formulas:

$$Sensitivty = \frac{\sum_{i=1}^{n} max_{j=1}^{m} t_{ij}}{\sum_{i=1}^{n} |b_i|},$$

$$PPV = \frac{\sum_{j=1}^{m} max_{i=1}^{n} T_{ij}}{\sum_{j=1}^{m} \sum_{i=1}^{n} T_{ij}}, and$$

$$Accuracy = \sqrt{Sensitivty \times PPV},$$

where'i' is the index of the protein complex from 1 to n and 'j' is the index of the predicted cluster from 1 to m. t_{ij} and T_{ij} is the number of overlapping proteins between $complex_i$ and $cluster_j$, and $|b_i|$ is the size of $complex_i$. The MMR builds on the maximal matching of the overlap score for each reference complex and all the predicted clusters and is calculated by dividing the sum of the matched overlap score by the number of reference complexes.

Abbreviations

AP-MS: affinity purification/mass spectrometry; AUC: area under the curve; BP: biological process; CC: cellular component; CF-MS: co-fractionation/mass spectrometry; CNN: convolutional neural network; DAG: directed acyclic graph; FCGR: frequency matrix chaos game representation; FN: false negative; FP: false positive; MCC: Matthews correlation coefficient; MCL: Markov cluster algorithm; MF: molecular function; MI: mutual information; MMR: maximum matching ratio; PPIs: protein–protein interactions; PPV: positive predictive value; RF: random forest; ROC: receiver operating characteristic; TN: true negative; TOM: topological overlap matrix; TP: true positive; t-SNE: t-distributed stochastic neighbor embedding; WCC: weighted cross correlation; Y2H: yeast-two-hybrid.

Acknowledgments

We gratefully acknowledge all data contributors and their submitting laboratories for generating the CF-MS data and sharing them on public resources [14,29].

Author Contributions

Y.H. worked on the conceptualization and design of the pipeline and participated in the implementation of models, analysis and visualization of results, and wrote the original draft. C.F. helped with concept development and structural modification of results as well as confirming the integrity of the analytical architecture. J.Y. and H.K. were involved in reviewing and editing of the manuscript. All authors read and approved the final manuscript.

Funding

This work was supported by Academia Sinica, Taiwan (AS-GC-110-L15) and the National Science and Technology Council, Taiwan (NSTC112-2221-E-001-021-MY3).

Availability of Source Code and Requirements

Project name: FREEPII
Project homepage: https://github.com/qqpigass/FREEPII

Operating system(s): Platform independent
Programming language: Python, Jupyter Notebook, R
License: GNU GPL.
RRID: Not created
WorkflowHub DOI: Not created

Data availability

The source code for this study is available on GitHub (https://github.com/qqpigass/FREEPII). The human CF-MS datasets (PXD002892, PXD014820, PXD015406) can be directly downloaded from Zenodo (doi: 10.5281/zenodo.4106578), while the yeast (*S. cerevisiae*) CF-MS datasets have been deposited to the ProteomeXchange Consortium *via* the PRIDE [43] partner repository with the dataset identifier PXD031967.

Competing Interests

The authors declare that they have no competing interests.

Ethics approval and consent to participate

Not applicable.

Consent for publication

Not applicable.

References

1. Bludau I, Aebersold R. Proteomic and interactomic insights into the molecular basis of cell functional diversity. Nat Rev Mol Cell Biol 2020;21(6):327-340. https://doi.org/10.1038/s41580-020-0231-2.

2. Larance M, Lamond AI. Multidimensional proteomics for cell biology. Nat Rev Mol Cell Biol 2015;16(5):269-80. https://doi.org/10.1038/nrm3970.

3. Cheng F, Zhao J, Wang Y, Lu W. Comprehensive characterization of protein–protein interactions perturbed by disease mutations. Nat Genet 2021;53(3):342-353. https://doi.org/10.1038/s41588-020-00774-y.

4. Lu H, Zhou Q, He J, Jiang Z, Peng C, Tong R, et al. Recent advances in the development of protein–protein interactions modulators: mechanisms and clinical trials. Signal Transduct Target Ther 2020;5(1):213. https://doi.org/10.1038/s41392-020-00315-3.

5. Huttlin EL, Bruckner RJ, Paulo JA, Cannon JR, Ting L, Baltier K, et al. Architecture of the human interactome defines protein communities and disease networks. Nature 2017;545(7655):505-509. https://doi.org/10.1038/nature22366.

6. Paiano A, Margiotta A, De Luca M, Bucci C. Yeast two-hybrid assay to identify interacting proteins. Curr Protoc Protein Sci 2019;95(1):e70. https://doi.org/10.1002/cpps.70.

7. Luck K, Kim DK, Lambourne L, Spirohn K, Begg BE, Bian W, et al. A reference map of the human binary protein interactome. Nature 2020;580(7803):402-408. https://doi.org/10.1038/s41586-020-2188-x.

8. Duarte CEM, Euclydes NC. Protein–protein interaction via two-hybrid assay in yeast. Methods Mol Biol 2024;2724:193-210. https://doi.org/10.1007/978-1-0716-3485-1_14.

9. Huttlin EL, Bruckner RJ, Navarrete-Perea J, Cannon JR, Baltier K, Gebreab F, et al. Dual proteome-scale networks reveal cell-specific remodeling of the human interactome. Cell 2021;184(11):3022-3040.e28. https://doi.org/10.1016/j.cell.2021.04.011.

10. Gnanasekaran P, Pappu HR. Affinity purification-mass spectroscopy (AP-MS) and co-immunoprecipitation (Co-IP) technique to study protein–protein interactions. Methods Mol Biol 2023;2690:81-85. https://doi.org/10.1007/978-1-0716-3327-4_7.

11. Salas D, Stacey RG, Akinlaja M, Foster LJ. Next-generation interactomics: considerations for the use of co-elution to measure protein interaction networks. Mol Cell Proteomics 2020;19(1):1-10. https://doi.org/10.1074/mcp.R119.001803.

12. McBride Z, Chen D, Lee Y, Aryal UK, Xie J, Szymanski DB. A label-free mass spectrometry method to predict endogenous protein complex composition. Mol Cell Proteomics 2019;18(8):1588-1606. https://doi.org/10.1074/mcp.RA119.001400.

13. Foster LJ, de Hoog CL, Zhang Y, Zhang Y, Xie X, Mootha VK, et al. A mammalian organelle map by protein correlation profiling. Cell 2006;125(1):187-199. https://doi.org/10.1016/j.cell.2006.03.022.

14. Skinnider MA, Foster LJ. Meta-analysis defines principles for the design and analysis of co-fractionation mass spectrometry experiments. Nat Methods 2021;18(7):806-815. https://doi.org/10.1038/s41592-021-01194-4.

15. Stacey RG, Skinnider MA, Scott NE, Foster LJ. A rapid and accurate approach for prediction of interactomes from co-elution data (PrInCE). BMC Bioinformatics 2017;18(1):457. https://doi.org/10.1186/s12859-017-1865-8.

16. Hu LZM, Goebels F, Tan JH, Wolf E, Kuzmanov U, Wan C, et al. EPIC: software toolkit for elution profile-based inference of protein complexes. Nat Methods 2019;16(8):737-742. https://doi.org/10.1038/s41592-019-0461-4.

17. Skinnider MA, Cai C, Stacey RG, Foster LJ. PrInCE: an R/Bioconductor package for protein-protein interaction network inference from co-fractionation mass spectrometry data. Bioinformatics 2021;37(17):2775-2777. https://doi.org/10.1093/bioinformatics/btab022.

18. Chen YH, Chao KH, Wong JY, Liu CF, Leu JY, Tsai HK. A feature extraction free approach for protein interactome inference from co-elution data. Brief Bioinform 2023;24(4):bbad229. https://doi.org/10.1093/bib/bbad229.

19. Yu M, Gormley MR, Dredze M. Combining word embeddings and feature embeddings for fine-grained relation extraction. Proceedings of the 2015 Conference of the North American Chapter of the Association for Computational Linguistics: Human Language Technologies 2015;1374–1379. Denver, Colorado. ACL. https://doi.org/10.3115/v1/N15-1155.

20. Kan S, Cen Y, He Z, Zhang Z, Zhang L, Wang Y. Supervised deep feature embedding with handcrafted feature. IEEE Trans Image Process 2019;28(12):5809-5823. https://doi.org/10.1109/TIP.2019.2901407.

21. Zhao C, Wang Z. GOGO: An improved algorithm to measure the semantic similarity between gene ontology terms. Sci Rep 2018;8(1):15107. https://doi.org/10.1038/s41598-018-33219-y.

22. El Adoui M, Drisis S, Benjelloun M. Multi-input deep learning architecture for predicting breast tumor response to chemotherapy using quantitative MR images. Int J Comput Assist Radiol Surg 2020;15(9):1491-1500. https://doi.org/10.1007/s11548-020-02209-9.

23. Tsietso D, Yahya A, Samikannu R, Tariq MU, Babar M, Qureshi B, et al. Multi-input deep learning approach for breast cancer screening using thermal infrared imaging and clinical data. IEEE Access 2023;11: 52101-52116. https://doi.org/10.1109/ACCESS.2023.3280422.

24. Jumper J, Evans R, Pritzel A, Green T, Figurnov M, Ronneberger O, et al. Highly accurate protein structure prediction with AlphaFold. Nature 2021;596(7873):583-589. https://doi.org/10.1038/s41586-021-03819-2.

25. Elnaggar A, Heinzinger M, Dallago C, Rehawi G, Wang Y, Jones L, et al. ProtTrans: toward understanding the language of life through self-supervised learning. IEEE Trans Pattern Anal Mach Intell 2022;44(10):7112-7127. https://doi.org/10.1109/TPAMI.2021.3095381.

26. Singh R, Devkota K, Sledzieski S, Berger B, Cowen L. Topsy-Turvy: integrating a global view into sequence-based PPI prediction. Bioinformatics 2022;38(Suppl 1):i264-i272. https://doi.org/10.1093/bioinformatics/btac258.

27. Ghorbani A, Zou JY. Embedding for informative missingness: deep learning with incomplete data. 56th Annual Allerton Conference on Communication, Control, and Computing (Allerton) 2018;437-445. Monticello, IL, USA. IEEE. https://doi.org/10.1109/ALLERTON.2018.8636008.

28. Mikołajczyk A, Grochowski M. Data augmentation for improving deep learning in image classification problem. International Interdisciplinary PhD Workshop (IIPhDW) 2018; 117-122. Świnouście, Poland. https://doi.org/10.1109/IIPHDW.2018.8388338.

29. Swamy KBS, Lee HY, Ladra C, Liu CFJ, Chao JC, Chen YY, et al. Proteotoxicity caused by perturbed protein complexes underlies hybrid incompatibility in yeast. Nat Commun 2022;13(1):4394. https://doi.org/10.1038/s41467-022-32107-4.

30. Tsitsiridis G, Steinkamp R, Giurgiu M, Brauner B, Fobo G, Frishman G, et al. CORUM: the comprehensive resource of mammalian protein complexes-2022. Nucleic Acids Res 2023;51(D1):D539-D545. https://doi.org/10.1093/nar/gkac1015.

31. UniProt Consortium. UniProt: the universal protein knowledgebase in 2023. Nucleic Acids Res 2023;51(D1):D523-D531. https://doi.org/10.1093/nar/gkac1052.

32. Chan EYS, Corless RM. Chaos game representation. SIAM Review 2023;65: 261-290. https://doi.org/10.48550/arXiv.2012.09638.

33. Almeida JS, Carriç JA, Ant´ A, Maretzek A, Noble PA, Fletcher M. Analysis of genomic sequences by Chaos Game Representation. Bioinformatics 2001;17(5):429-37. https://doi.org/10.1093/bioinformatics/17.5.429.

34. Lö Chel HF, Eger D, Sperlea T, Heider D. Deep learning on chaos game representation for proteins. Bioinformatics 2020;36(1):272-279. https://doi.org/10.1093/bioinformatics/btz493.

35. Löchel HF, Heider D. Chaos game representation and its applications in bioinformatics. Comput Struct Biotechnol J 2021;19:6263-6271. https://doi.org/10.1016/j.csbj.2021.11.008.

36. Kadir T, Brady M. Saliency, scale and image description. Int J Comput Vis 2001;45:83-105. https://doi.org/10.1023/A:1012460413855.

37. Dongen S Van. Graph clustering via a discrete uncoupling process. SIAM J Matrix Anal Appl 2008;30(1): 121-141. https://doi.org/10.1137/040608635.

38. Enright AJ, Dongen S Van, Ouzounis CA. An efficient algorithm for large-scale detection of protein families. Nucleic Acids Res 2002;30(7):1575-1584. https://doi.org/10.1093/nar/30.7.1575.

39. Yip AM, Horvath S. Gene network interconnectedness and the generalized topological overlap measure. BMC Bioinformatics 2007;8:22. https://doi.org/10.1186/1471-2105-8-22.

40. Wang JZ, Du Z, Payattakool R, Yu PS, Chen CF. A new method to measure the semantic similarity of GO terms. Bioinformatics 2007;23(10):1274-1281. https://doi.org/10.1093/bioinformatics/btm087.

41. Krumsiek J, Zimmer R, Friedel CC. Bootstrapping the Interactome: Unsupervised Identification of Protein Complexes in Yeast. J Comput Biol 2009;16(8):971-87. https://doi.org/10.1089/cmb.2009.0023.

42. Nepusz T, Yu H, Paccanaro A. Detecting overlapping protein complexes in protein-protein interaction networks. Nat Methods 2012;9(5):471-2. https://doi.org/10.1038/nmeth.1938.

43. Perez-Riverol Y, Bai J, Bandla C, García-Seisdedos D, Hewapathirana S, Kamatchinathan S, et al. The PRIDE database resources in 2022: a hub for mass spectrometry-based proteomics evidences. Nucleic Acids Res 2022;50(D1):D543-D552. https://doi.org/10.1093/nar/gkab1038.

Table 1. CF-MS data information. The human CF-MS datasets (PXD002892, PXD014820, and PXD015406) were downloaded from Zenodo (doi: 10.5281/zenodo.4106578), and the yeast CF-MS dataset (PXD031967) was curated by ourselves. Data description and pre-processing steps are detailed in Materials and Methods.

Species	Accession	Experimental name	Abbreviation	Number of fractionations	Number of proteins	Number of proteins in protein complexes
Human	PXD002892	SEC2_H	H1	55	4002	1720
Human	PXD002892	SEC3_H	H2	55	4563	1909
Human	PXD014820	Ctrl	H3	61	5268	1986
Human	PXD015406	Control	H4	61	6043	2225
Yeast	PXD031967	Hsp90_20200122_H	Y1	27	2397	1089
Yeast	PXD031967	Ctrl_20200416_H	Y2	27	2753	1218
Yeast	PXD031967	Ctrl_20191126_H	Y3	27	2026	933
Yeast	PXD031967	Hsp90_20201028_H	Y4	27	2952	1309

Figure 1. Analysis pipeline of FREEPII. The input of FREEPII consists of three parts, including: CF-MS data containing information of protein interactions in experiments (matrix A), protein sequence data (in FCGR form, see Methods) with conserved information between proteins (matrix B), and protein embeddings (matrix C) that encapsulate protein interaction information in protein complexes after training. To form the final input, matrix B is added to matrix C and then is concatenated with matrix A. FREEPII completes feature extraction and map creation through a convolutional layer, and generates protein feature representations by flattening the feature map along the filter dimension. The protein pair index is used as an additional input to extract and construct PPI feature representations, which are then fed into a fully connected layer. The output, comprising predicted PPI scores together with the protein pairing index, is subsequently used for clustering analysis to predict protein complexes. Notably, N represents the number of proteins, M denotes the number of fractionations, M' is the length of the flattened FCGR of the protein sequence and protein embedding, and F indicates the length of a protein's feature representation.

Figure 2. PPI classification performance. Four scoring metrics, including Sensitivity, Specificity, MCC and AUC of ROC, are used to evaluate the performance of A) existing CF-MS analysis tools and B) models with various structures and inputs in the ablation study on the PPI classification task. All results are based on predictions from the testing set. Specifically, RF-C represents the random forest model using only CF-MS features as input, CNN-C denotes CNN using solely CF-MS data as input without incorporating protein sequences, and FREEPII(-) indicates FREEPII without incorporating protein embeddings.

Figure 3. FREEPII exploits two inputs for classification and learns discriminative feature representations. A) Average difference in intensity between CF-MS and protein sequence input regions using the saliency map. This is generated by applying FREEPII to the yeast dataset Y2. It visualizes the intensity contrast between CF-MS and protein sequence input regions, derived from 10000 PPIs. B) The saliency maps depicted three categories of PPIs, CF-MS~seq, CF-MS<seq, and CF-MS>seq, based on the degree to which FREEPII considers co-elution profiles or protein sequences during prediction. The prediction results are derived from FREEPII's application to yeast dataset Y2. For clarity, the padding part of CF-MS data has been removed, and only the first 100 units of the protein sequence region are displayed to avoid compressing the CF-MS data. A grey line is manually inserted to separate the two inputs visually. C) Feature representations of proteins learned by models via t-SNE (using yeast dataset Y2). The t-SNE visualization for all human and yeast dataset are shown in Figure S4 and Figure S5, respectively. Different colors represent labels for different protein complexes. For clarity, only 11 yeast protein complexes are labelled. Cosine distances between pairs of protein feature representations within and between protein complexes are calculated, and the Kruskal-Wallis test is used to assess whether the difference in distance distribution between the two groups is significant. *ns*: $p > 0.05$, *: $p \leq 0.05$, **: $p \leq 0.01$, ***: $p \leq 0.001$, ****: $p \leq 0.0001$.

Figure 4. Cluster quality assessment. To assess the quality of clusters produced by each model's predicted PPIs, three scoring metrics including composite, GOGO, and co-localization scores are employed. (A) illustrates the evaluation through the composite score, which is the sum of overlap score (red), accuracy (blue), and MMR (green). (B) and (C) respectively depict the GOGO (BP ontology, see Methods) and co-localization scores, with the dashed lines represent the average across various models in each figure.

Figure 5. Cross prediction and co-training. A). Models were trained on either a single co-elution dataset (H1 or Y1) or co-trained using multiple training sets (H1+Y1 and H1+H3+Y1). The prediction performance is evaluated on their own testing set and on the testing sets of other co-elution datasets. The *x*-axis indicates the training datasets, while the panels represent the species names for the testing set. Each boxplot is the distribution of performance scores of the trained model on the testing sets across all experiments for that species. B) The growth curve displays the average performance score of each model across all testing sets, comparing the scheme of single-training (H1/Y1) to co-training (H1+Y1, H1+H3+Y1). To ensure balanced contributions of different training sets to model learning, we down-sampled the training pairing indices from various training sets to match the smallest training set's index.

Figure 1

Click here to access/download;Figure;fig1.jpg

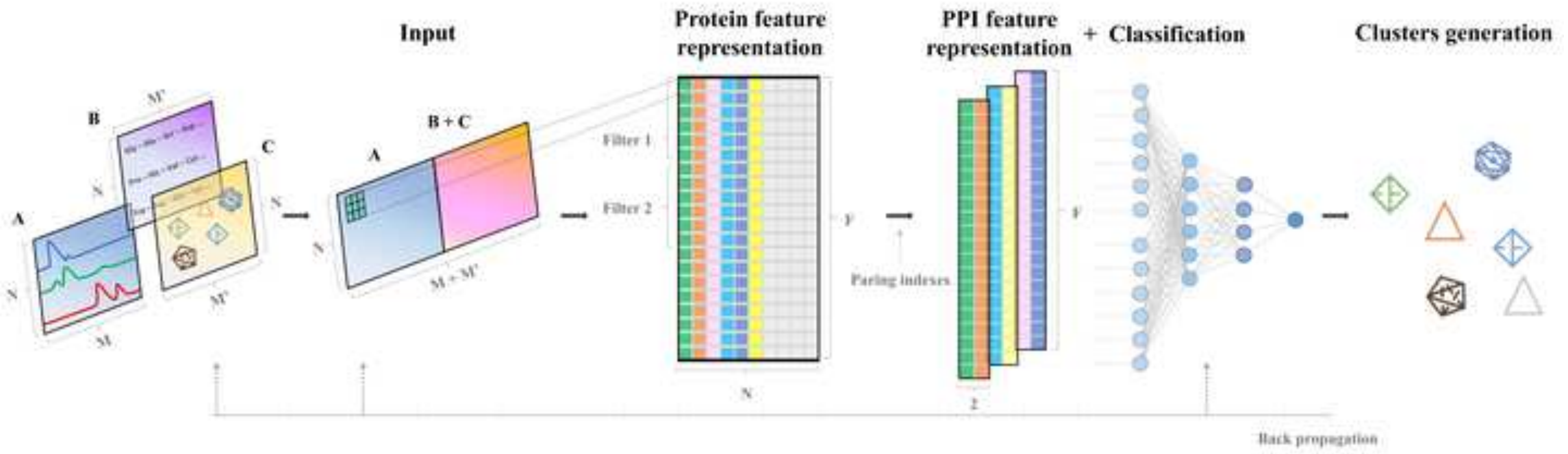

Figure 2 Click here to access/download;Figure;fig2.png

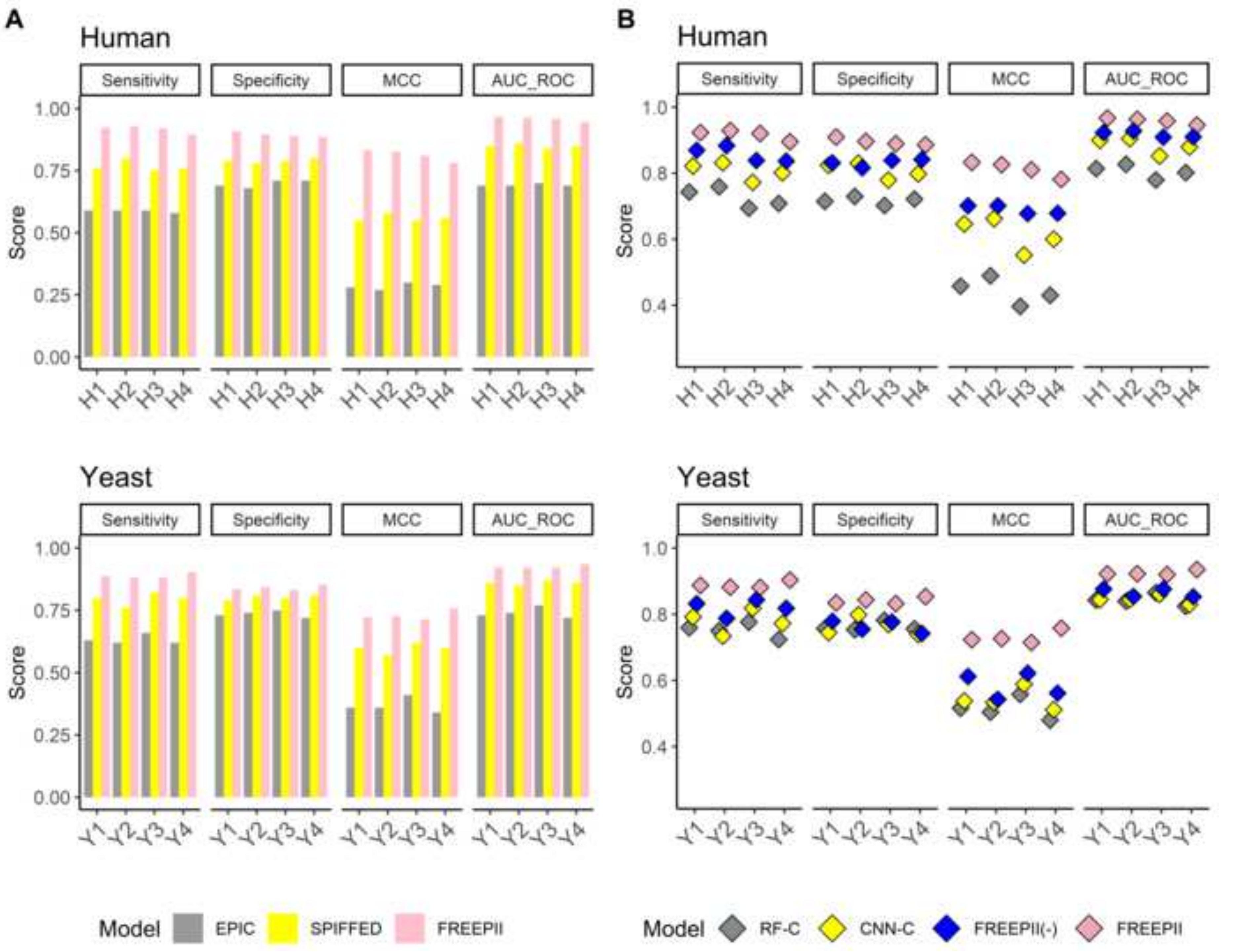

Figure 3 Click here to access/download;Figure;fig3.png

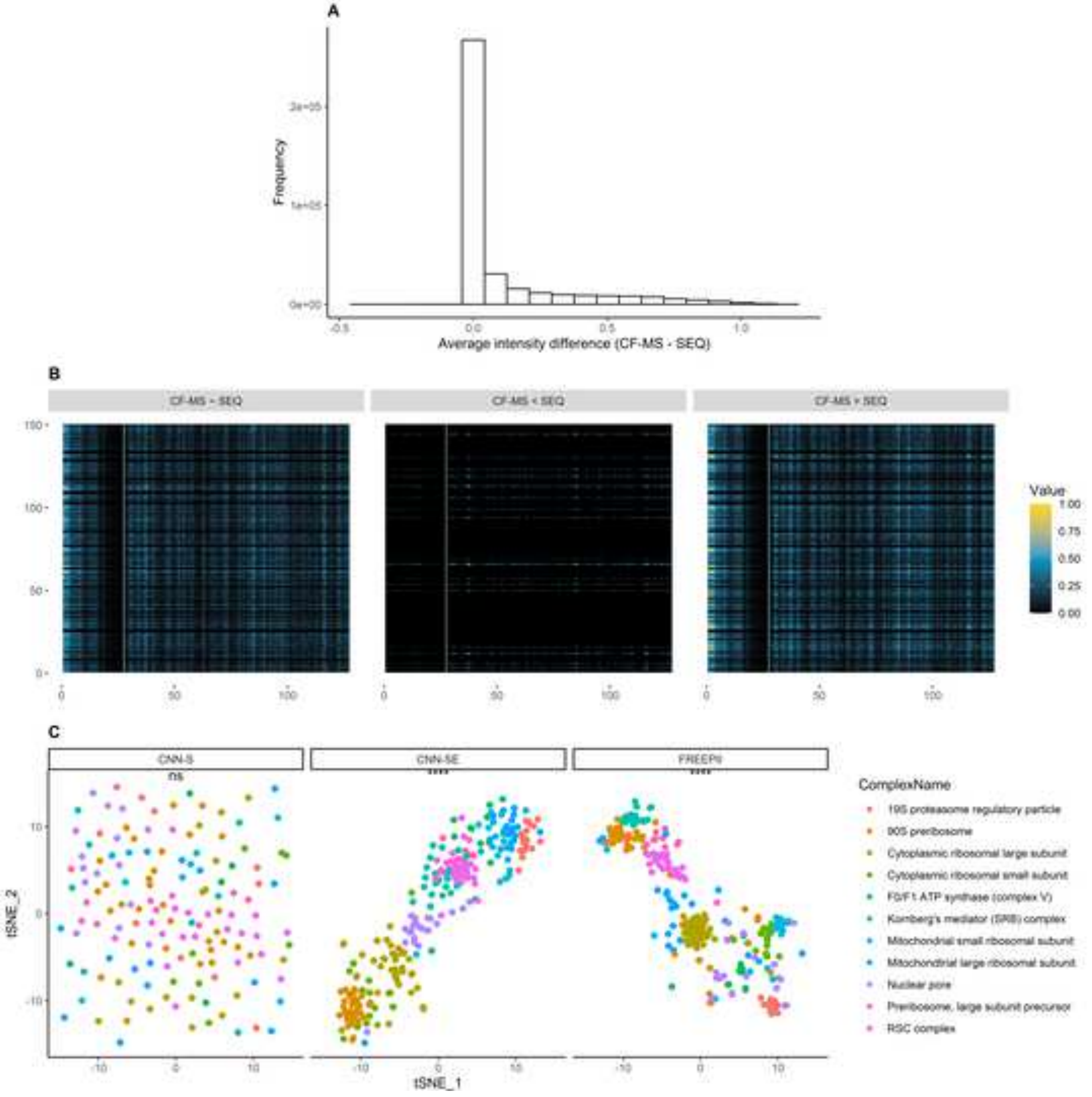

Figure 4

Click here to access/download;Figure;fig4.png

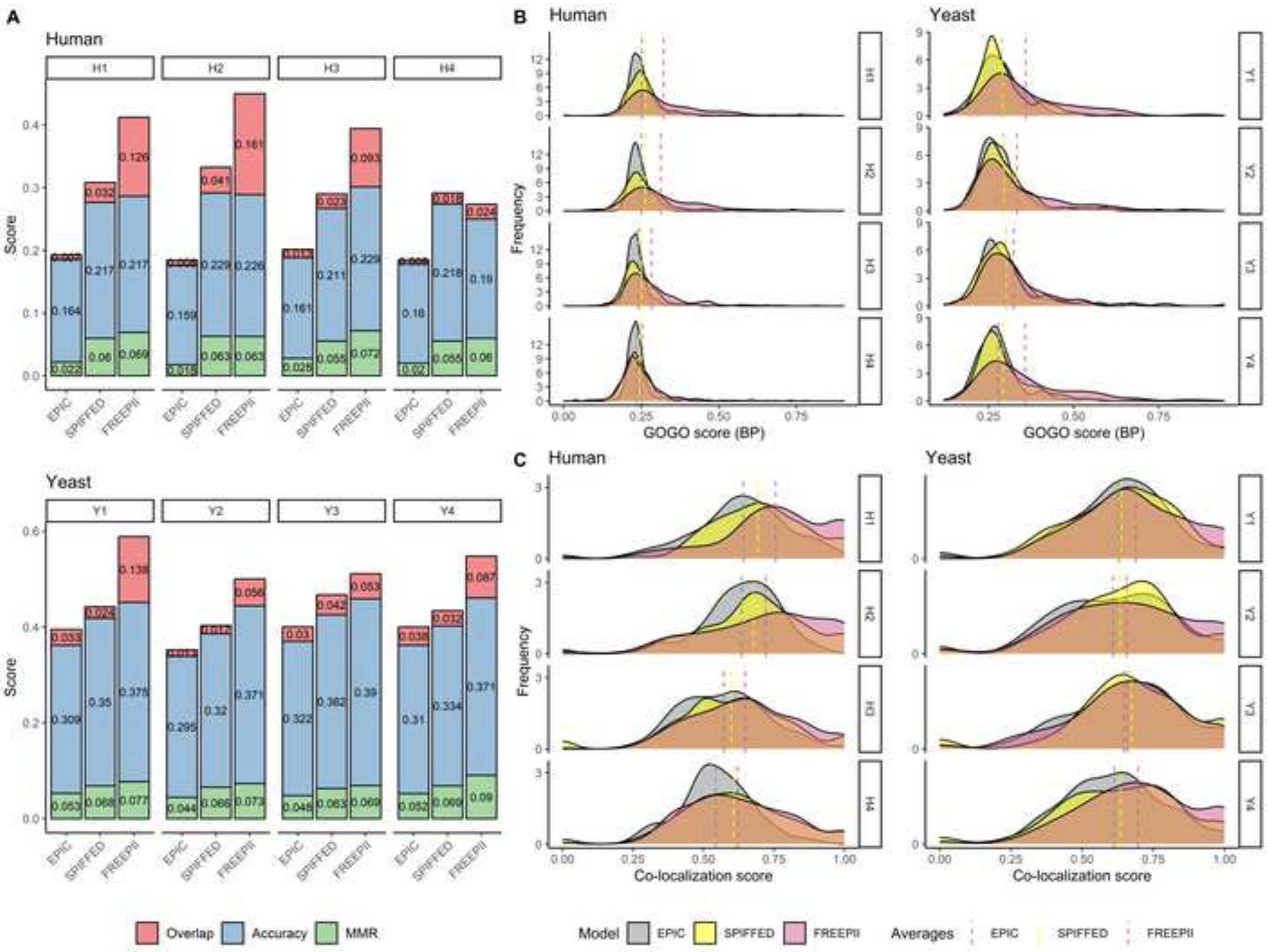

Figure 5

Click here to access/download;Figure;fig5.png

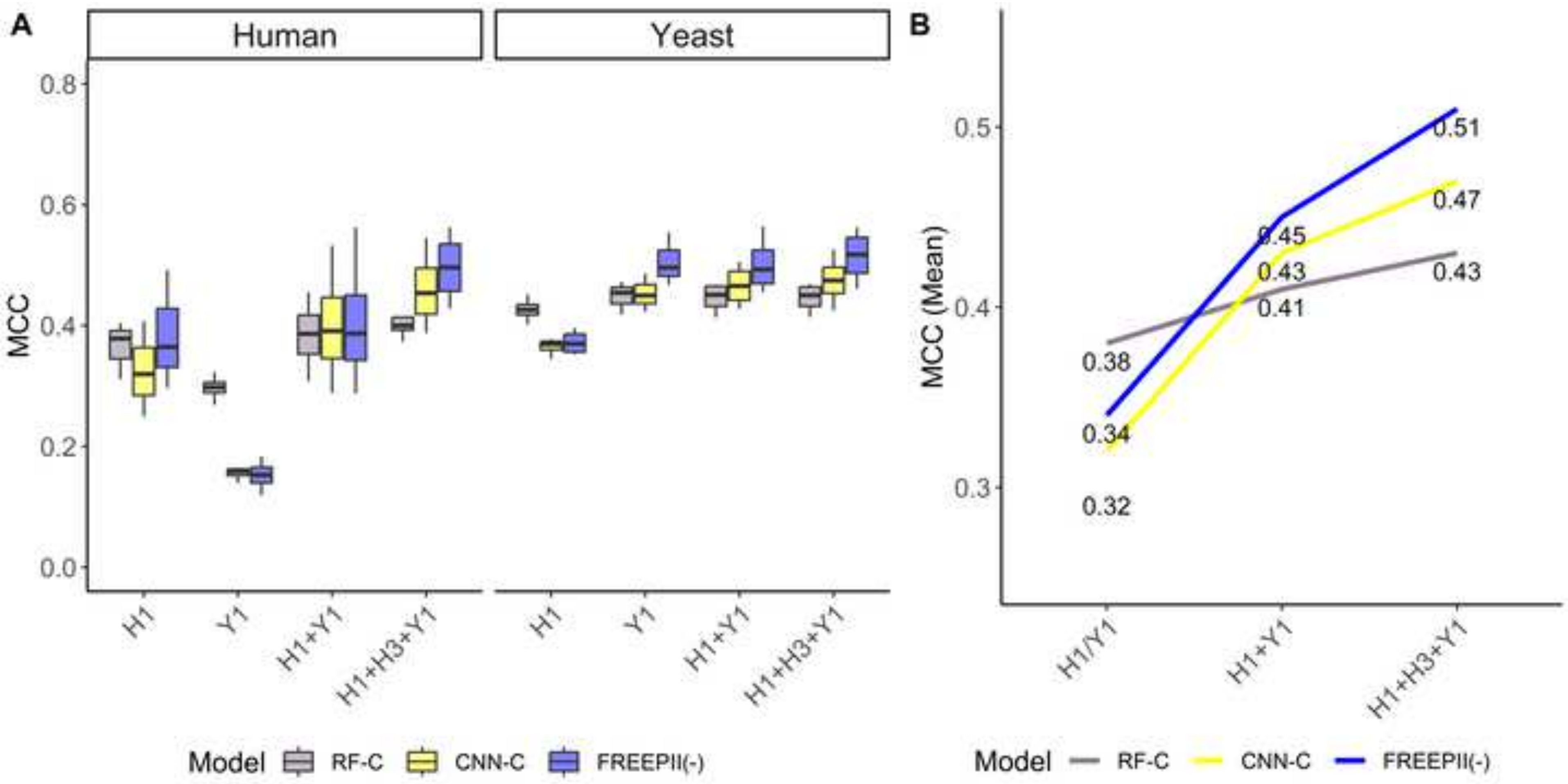

Click here to access/download
Supplementary Material
FREEPII_20241009_Supplementary Material.pdf

Graphical Abstract Click here to access/download;Graphical Abstract;Graphical abstract.jpg

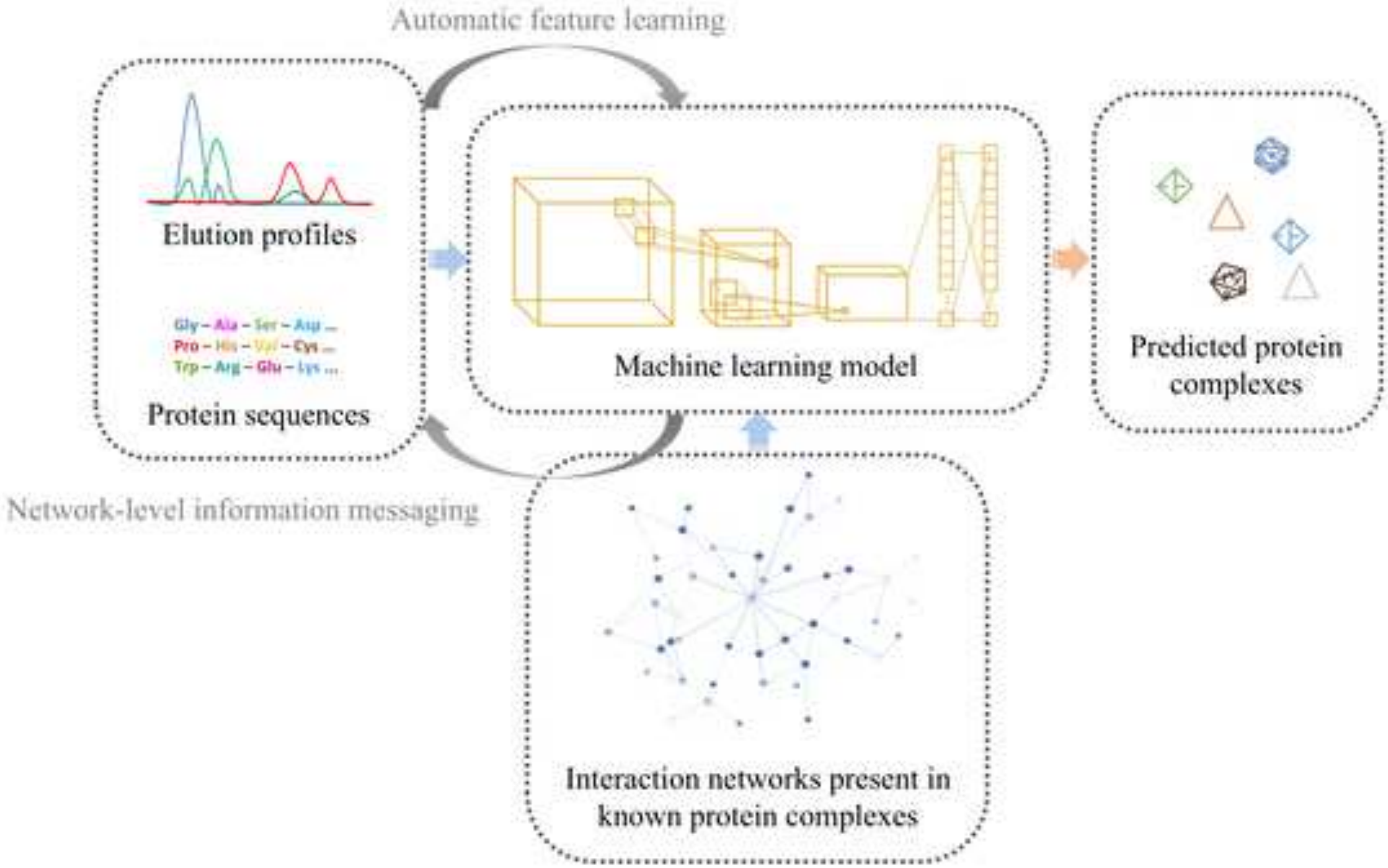

Supplement: giaf122_GIGA-D-25-00010_Original_Submission [file giaf122_giga-d-25-00010_original_submission.pdf]
