# Supplementary material for: Complete end-to-end learning from protein feature representation to protein interactome inference
Source: Gigascience. 2025 Nov 6;14:giaf122. doi: 10.1093/gigascience/giaf122 (PMC12598752; doi:10.1093/gigascience/giaf122)

GigaScience

Complete end-to-end learning from protein feature representation to protein interactome inference

--Manuscript Draft--

**Manuscript Number:**	GIGA-D-25-00010R3
**Full Title:**	Complete end-to-end learning from protein feature representation to protein interactome inference
**Article Type:**	Research
**Funding Information:**	National Science and Technology Council (NSTC112-2221-E-001-021-MY3) — PhD Huai-Kuang Tsai<br>Academia Sinica (AS-GC-110-L15) — PhD Jun-Yi Leu
**Abstract:**	Co-fractionation coupled with mass spectrometry (CF-MS) is a powerful strategy for mapping protein-protein interactions (PPIs) under near-physiological conditions. Despite recent progress, existing analysis pipelines remain constrained by reliance on handcrafted features, sensitivity to experimental noise, and an inherent focus on pairwise interactions, which limit their scalability and generalizability. To address these difficulties, we introduce FREEPII (Feature Representation Enhancement End-to-End Protein Interaction Inference), a unified deep learning framework that integrates CF-MS data with sequence-derived features to learn biologically meaningful protein-level representations for accurate and efficient inference of PPIs and protein complexes. FREEPII employs a convolutional neural network (CNN) architecture to learn protein-level representations directly from raw data, enabling feature sharing across interaction pairs and reducing computational complexity. To enhance robustness against CF-MS noise, protein sequences are introduced as auxiliary input to enrich the feature space with complementary biological cues. The supervised protein embeddings further encode network-level context derived from complex annotations, allowing the model to capture higher-order interactions and enhance the expressive power of protein representations. Extensive benchmarking demonstrates that FREEPII consistently outperforms state-of-the-art CF-MS analysis tools, capturing more biologically coherent and discriminative protein features. Cross-dataset evaluations further reveal that integrating multi-modal data from diverse experimental contexts substantially improves the generalization and sensitivity of data-driven models, offering a scalable, cross-species strategy for reliable protein interaction inference.
**Corresponding Author:**	Huai-Kuang Tsai<br>Institute of Information Science Academia Sinica<br>Taipei, 台北市 TAIWAN
**Corresponding Author Secondary Information:**	
**Corresponding Author's Institution:**	Institute of Information Science Academia Sinica
**Corresponding Author's Secondary Institution:**	
**First Author:**	Huai-Kuang Tsai
**First Author Secondary Information:**	
**Order of Authors:**	Huai-Kuang Tsai
	Yu-Hsin Chen
	Chien-Fu Liu
	Jun-Yi Leu
**Order of Authors Secondary Information:**	
**Response to Reviewers:**	We thank you for handling our manuscript entitled “Complete end-to-end learning from protein feature representation to protein interactome inference” (Manuscript ID: GIGA-D-25-00010R2). We deeply appreciate the opportunity to further improve our

Powered by Editorial Manager® and ProduXion Manager® from Aries Systems Corporation

manuscript in response to the reviewers' comments. In this revision, we provide a detailed point-by-point response outlining the revisions and clarifications made, along with additional edits to improve clarity and transparency. We would like to emphasize that all experimental design choices were carefully made to address the specific characteristics and challenges of CF-MS analysis. We sincerely hope the Editor and the reviewers find this revised version satisfactory, and please feel free to contact me if any further information is needed.

Reviewer #1:
Comment 1: " The authors given the performance of Tapioca in the response as follow "Tapioca's inability to be retrained on our datasets and its poor predictive performance (AUC-ROC of 0.652 and 0.611 on datasets H1 and Y1". Why the results heve not been added in the content? It is necessary to consider whether the issue stems from data compatibility problems rather than inherent limitations of the method itself. Also no details on hyperparameter tuning or implementation"

Response:
In our previous response, we reported Tapioca's performance (AUC-ROC of 0.652 and 0.611 on datasets H1 and Y1, respectively) using the pretrained model and default configuration provided by its authors. Since Tapioca does not support retraining, these results reflect its intended usage—trained on a large, diverse dataset for generalizability. Notably, our observations are consistent with those reported in Tapioca's Supplementary Table 1, suggesting that its limited performance in CF-MS-based PPI prediction arises from methodological constraints rather than data incompatibility.
Tapioca's internal architecture primarily relies on handcrafted features (e.g., regression and random forest), placing it in the same category as EPIC and PrInCE. In our prior work, we have thoroughly benchmarked deep-learning-based approaches against such handcrafted feature-based methods. Consistently, these models exhibit common limitations, including low sensitivity, predictive bias under imbalanced training data, and susceptibility to overfitting. This reinforces our conclusion that models relying solely on handcrafted features are generally inadequate for reliable PPI inference using CF-MS data.
Following the reviewers' suggestion, we have now included Tapioca's performance results in the Supplementary Materials (Table S5) for completeness and transparency, and we have added a brief discussion of these results in Discussion (Lines 226–229). While we initially chose not to highlight Tapioca in the main manuscript due to its lack of retraining capability and overlapping conclusions with other baseline tools such as EPIC, its supplementary inclusion ensures clarity and provides readers with direct access to the comparative results.

Comment 2: " How about the performance using protein embeddings generated by EMS2 or other large model."

Response:
We thank the reviewer for this insightful suggestion. Indeed, we have experimented with protein embeddings generated by large pretrained models, including ESM2 and ProtTrans. In several pilot experiments, these pretrained embeddings consistently achieved slightly superior model performance compared to flattened FCGR, suggesting that they capture richer biological information and offer representations more readily leveraged by downstream models.
However, generating embeddings from large pretrained models such as ProtTrans or ESM2 requires substantial computational resources—particularly for longer protein sequences—which becomes a limiting factor in large-scale applications. For this reason, we selected flattened FCGR as the primary sequence-based representation in this study, as it offers a favorable balance between informativeness and computational efficiency. FCGR is lightweight, length-invariant in output dimensionality, and scalable to thousands of proteins across multiple datasets.
Nonetheless, we fully recognize the value of pretrained embeddings. As described in Discussion (Lines 270-271), FREEPII is built with flexible, modality-aware input integration and is readily compatible with alternative sequence-based representations, including pretrained embeddings, structural descriptors, or expression-based features. While exploring pretrained embeddings lies beyond the scope of the present study, we encourage their application in future use of FREEPII, especially where sufficient

Powered by Editorial Manager® and ProduXion Manager® from Aries Systems Corporation

computational resources are available.

Comment 3: "Why the simple CNN is used? Some other networks should be compared to show the advantage of CNN."

Response:
We thank the reviewer for raising this important point regarding model selection. In addition to CNN, we explored alternative architectures in preliminary experiments, including graph convolutional networks (GCNs) and transformer-based models. These models achieved comparable performance in terms of classification metrics, with sensitivity and specificity differences generally within 0.1 and 0.2.
Our evaluation, however, considered not only on predictive accuracy but also on how well each model aligns with the structural characteristics of CF-MS data. We found that CNNs were particularly effective at capturing localized co-elution signals, which are essential for resolving partially overlapping or transient interactions. Supporting analysis, including visualization of elution profiles and quantitative comparisons (Euclidean distance, Pearson correlation), showed that CNN-based representations better preserved localized fluctuations, whereas transformer and GCN models tended to emphasize global similarity at the expense of these fine-grained patterns.
While CNNs did not always yield the single best numerical performance, we selected them because they most closely match the domain-specific signal properties of CF-MS data, provide better interpretability, reduce the risk of overfitting, and offer superior computational efficiency for large-scale applications. This rationale is further elaborated in Discussion (Lines 297-309). Looking forward, we plan to extend FREEPII with context-aware components such as attention mechanisms, where CNN layers serve as local feature extractors and transformer blocks model global dependencies. Such hybrid architectures could combine the strengths of local signal fidelity within broader contextual modeling.

Comment 4: " The element-wise addition of protein embeddings to FCGR (sequence) features (Page 12, Lines 378-379) ignores numerical scale disparities between modalities. CF-MS profiles (continuous) and FCGR matrices (discrete frequency counts) occupy vastly different ranges."

Response:
To clarify, in our implementation the element-wise addition is applied only between the protein embedding and the protein sequence FCGR representation. The resulting vector is then concatenated with the CF-MS elution profile, rather than added to it. This design preserves modality separation while enabling multimodal integration at a later stage of the network, a key architectural principle that we have emphasized throughout the manuscript (Results, Lines 95-96; Discussion, Lines 289-296). The purpose of element-wise addition between the embedding and FCGR is to enhance the expressiveness of sequence-derived frequency patterns and to compensate for potential sparsity or noise in the FCGR representation. Similar fusion strategies have been successfully adopted in prior studies (e.g. Ghorbani et al. 2018), and we have elaborated on this rationale in Discussion (Lines 277-279).
In addition, we have now made explicit in the manuscript (Results, Lines 95-96; Methods, Lines 380-381) that both CF-MS and FCGR inputs are normalized to the [0, 1] range to mitigate scale mismatches. After normalization, FCGR values are typically very small (often < 0.1), comparable to embedding values. By contrast, CF-MS features span a broader range (0.1~0.9), and therefore contribute stronger and more interpretable co-elution signals after concatenation. Under this configuration, protein embeddings serve as supplementary biological cues, complementing the dominant elution profile features. Empirically, we did not observe numerical instability or degraded performance with this approach, supporting its viability under the described conditions.
Ghorbani A, Zou JY. Embedding for informative missingness: deep learning with incomplete data. 56th Annual Allerton Conference on Communication, Control, and Computing (Allerton) 2018;437-445. Monticello, IL, USA. IEEE.

Comment 5: " CF-MS datasets are padded to a fixed size of 200 fractions (Page 12, Line 356) without justification. Padding may distort elution profiles (e.g., adding zeros

	to 27-fraction yeast data)."<br><br>Response:<br>We appreciate the reviewer's concern regarding potential distortion from padding. To clarify, our strategy pads zeros only at the end of each elution profile, thereby preserving the original shape and integrity of the original co-elution signal. The padded portion carries no information by design and functions solely to standardize input dimensions, which is similar to zero-padding in image processing, where the padding does not alter valid features but ensures dimensional consistency.<br>Importantly, in single-dataset training scenarios, padding does not influence model learning, as all samples within a dataset share the same elution profile length. The main purpose of this design is to support cross-dataset training and prediction, where a unified input length is required for joint modeling across datasets with varying resolution or fraction lengths. To further evaluate this choice, we compared zero-padding with linear interpolation to resize elution profiles to a common length. Both strategies yielded similar model performance, suggesting that model robustness depends more on the inherent resolution of the data than on the specific preprocessing method. Based on these observations, we selected padding as the simpler and less intrusive solution. We have now added a more detailed description of the padding method and its rationale in the revised manuscript (Methods, Lines 360-363), to ensure clarity and reproducibility.<br><br>Comment 6: " Negative PPIs are defined as proteins from different complexes (Page 12, Lines 365-367). This ignores that proteins can interact transiently across complexes."<br><br>We thank the reviewer for highlighting this important limitation. We agree that proteins from different annotated complexes may still engage in transient interactions, which are not fully captured under our current definition of negative PPIs. Unfortunately, due to the lack of comprehensive interaction datasets covering transient interactions, defining negatives as protein pairs from different complexes remains a common and practical approach in PPI prediction studies, particularly in the CF-MS domain. This strategy has also been adopted in prior works such as EPIC, PrInCE, and Tapioca.<br>To increase the reliability of positive labels, we followed the strategy used in EPIC and required that positive protein pairs exhibit co-elution evidence (i.e., shared peaks in their elution profiles) before being included as true interactions (Methods, Lines 373-374). For future improvements, we plan to refine the construction of negative sets by excluding protein pairs that, although annotated as belonging to different complexes, also exhibit strong co-elution signals or are co-localized in the same subcellular compartment. Such refinement may reduce label noise and better delineate uncertain interactions.
**Additional Information:**	
**Question**	**Response**
Are you submitting this manuscript to a special series or article collection?	No
**Experimental design and statistics**<br><br>Full details of the experimental design and statistical methods used should be given in the Methods section, as detailed in our Minimum Standards Reporting Checklist. Information essential to interpreting the data presented should be made available in the figure legends.<br><br>Have you included all the information	Yes

Powered by Editorial Manager® and ProduXion Manager® from Aries Systems Corporation

requested in your manuscript?	
**Resources**<br><br>A description of all resources used, including antibodies, cell lines, animals and software tools, with enough information to allow them to be uniquely identified, should be included in the Methods section. Authors are strongly encouraged to cite Research Resource Identifiers (RRIDs) for antibodies, model organisms and tools, where possible.<br><br>Have you included the information requested as detailed in our Minimum Standards Reporting Checklist?	Yes
**Availability of data and materials**<br><br>All datasets and code on which the conclusions of the paper rely must be either included in your submission or deposited in publicly available repositories (where available and ethically appropriate), referencing such data using a unique identifier in the references and in the "Availability of Data and Materials" section of your manuscript.<br><br>Have you have met the above requirement as detailed in our Minimum Standards Reporting Checklist?	Yes
GigaScience has policies and guidelines in place for the use of generative AI-writing tools such as ChatGPT. If you have used such writing tools to assist with writing the manuscript this must be declared and cited in the text. Authors should not list AI-writing tools and other AI-assisted technologies as an author or co-author and should acknowledge that they are fully responsible for text generated or refined by AI-writing tools.<p><br><br>A summary of use (particularly in the introduction or among methods) needs to be included at the end of the paper, and	No

the outputs should also be included as a supplementary file hosted in GigaDB or other open repositories. Please read our guidelines for more information. <p><br><br>By submitting to GigaScience, you are aware of the journal's AI-writing tools policy, and if you have declared use of such tools below, you have acknowledged this where appropriate in your manuscript and have made a summary of use and outputs available. <p><br><b>AI-assisted writing tools have been used in the preparation of this manuscript?	

Manuscript

Click here to access/download;Manuscript;FREEPII_0929_final.docx

Complete end-to-end learning from protein feature representation to protein interactome inference

Yu-Hsin Chen[1], Chien-Fu Liu[2], Jun-Yi Leu[2,*], and Huai-Kuang Tsai[1,*]

[1] Institute of Information Science, Academia Sinica, Taipei, 11529, Taiwan
[2] Institute of Molecular Biology, Academia Sinica, Taipei, 11529, Taiwan

* To whom correspondence should be addressed. Tel: +886 2 2788 3799 (Ext 1718); Fax: +886 2 2782 4814; Email: hktsai@iis.sinica.edu.tw

Correspondence may also be addressed to Jun-Yi Leu. Tel: +886 2 2789 9216; Fax: +886 2 2651 9574; Email: jleu@imb.sinica.edu.tw

ORCID iDs: Jun-Yi Leu [0000-0001-5445-2799]; Huai-Kuang Tsai [0000-0002-4200-8137];

Abstract

Co-fractionation coupled with mass spectrometry (CF-MS) is a powerful strategy for mapping protein-protein interactions (PPIs) under near-physiological conditions. Despite recent progress, existing analysis pipelines remain constrained by reliance on handcrafted features, sensitivity to experimental noise, and an inherent focus on pairwise interactions, which limit their scalability and generalizability. To address these difficulties, we introduce FREEPII (Feature Representation Enhancement End-to-End Protein Interaction Inference), a unified deep learning framework that integrates CF-MS data with sequence-derived features to learn biologically meaningful protein-level representations for accurate and efficient inference of PPIs and protein complexes. FREEPII employs a convolutional neural network (CNN) architecture to learn protein-level representations directly from raw data, enabling feature sharing across interaction pairs and reducing computational complexity. To enhance robustness against CF-MS noise, protein sequences are introduced as auxiliary input to enrich the feature space with complementary biological cues. The supervised protein embeddings further encode network-level context derived from complex annotations, allowing the model to capture higher-order interactions and enhance the expressive power of protein representations. Extensive benchmarking demonstrates that FREEPII consistently outperforms state-of-the-art CF-MS analysis tools, capturing more biologically coherent and discriminative protein features. Cross-dataset evaluations further reveal that integrating multi-modal data from diverse experimental contexts substantially improves the generalization and sensitivity of data-driven models, offering a scalable, cross-species strategy for reliable protein interaction inference.

Keywords: co-fractionation coupled with mass spectrometry analysis, protein interactome inference, convolutional neural network, end-to-end learning, representation learning

Background

Proteins play a central role in biological processes such as catalytic reactions, signal transduction, immune responses, and molecule transportation [1–3]. These biological activities are often executed or regulated through protein-protein interactions (PPIs), forming a complex network called the protein interactome. Deciphering the local structure of the protein interactome (protein complexes) as well as completing the global structure of the protein interactome is critical for understanding cellular functions and disease mechanisms [4–6]. Several techniques have been used to analyse transient and stable PPIs and protein complexes, including yeast-two-hybrid (Y2H) screens [7–9], affinity purification coupled to tandem mass spectrometry (AP-MS) [10,11], and protein co-fractionation coupled to mass spectrometry (CF-MS) [12–16].

To date, the results of Y2H and AP-MS analysis have been used to establish approximately 53,000 [8] and over 56,000 [6] protein-protein interactions in human cells, respectively. Nonetheless, both systems have their limitations. The Y2H screen assay can only detect binary interactions in one experiment and requires the target proteins to be expressed in yeast cells, which may affect PPIs due to inappropriate modifications or incorrect localizations of proteins. Additionally, Y2H screens cannot determine PPIs in a specific cellular state. Instead, AP-MS can obtain protein interactions under specific conditions and map multiple protein interactions in parallel. However, AP-MS often requires specific antibodies or genetic engineering, which may alter protein structure and interaction sites. Furthermore, AP-MS can only detect stable interactions and will miss unstable or transient interactions.

CF-MS offers a powerful, high-throughput strategy for mapping PPIs at scale. Unlike genetic or affinity-based methods, CF-MS operates without exogenous perturbations, enabling the interrogation of protein associations under near-physiological conditions. The core principle of CF-MS involves measuring co-elution profiles across chromatographic fractions and inferring PPIs through correlation-based scoring and downstream graph partitioning algorithms for de novo protein complex detection [12,15]. However, the sensitivity and specificity of these inferences are often constrained by the reliance on hand-crafted features [17–21], which compress the data and introduce biases. Moreover, CF-MS data are inherently noisy, further complicating accurate interaction assignment. To address these limitations, our previous work introduced SPIFFED, a CNN-based end-to-end model that bypasses hand-crafted features by directly learning informative features from CF-MS data [22]. SPIFFED outperformed PrInCE [18,20] and EPIC [19], two widely adopted and representative CF-MS analysis tools that rely on predefined similarity metrics and manually selected features, validating the advantage of deep learning in CF-MS analysis. Despite this progress, SPIFFED retains architectural limitations: it models interactions strictly at the protein-pair level, which hinders the learning of transferable protein-level features and limits the capture of higher-order interaction patterns. Additionally, its reliance solely on CF-MS co-elution data limits sensitivity when signals are weak or ambiguous.

Here, we introduce FREEPII (Feature Representation Enhancement End-to-End Protein Interaction Inference), a unified deep learning framework that integrates CF-MS data with sequence-derived features and learns discriminative protein-level representations for accurate and scalable protein interaction inference. FREEPII also adopts a CNN-based architecture that learns directly from raw elution profiles while constructing protein-level representations shared across interaction pairs. This design reduces computational complexity from $2*N*(N-1)*M$ to $N*M$ (where N is the number of proteins and M is the number of CF-MS fractions), enabling more consistent and transferable feature learning. To enhance sensitivity, particularly when CF-MS signals are weak or ambiguous, FREEPII incorporates protein sequence features as auxiliary inputs. In addition, the supervised protein embeddings capture higher-order interaction contexts from complex-level annotations, enabling the model to internalize network-level dependencies beyond pairwise interactions and enhance representation learning in a fully end-to-end manner [23,24]. Through comprehensive evaluation, FREEPII consistently outperforms established CF-MS analysis tools, including the widely recognized handcrafted feature-based tools EPIC and our previous deep learning-based baseline SPIFFED, demonstrating superior performance in both interaction prediction and complex-level organization. It highlights the effectiveness of FREEPII's design in supporting accurate inference across diverse biological contexts. The examination of the learned representations illustrates that FREEPII's architectural design facilitates the learning of more informative and discriminative protein representations. Cross-dataset experiments further demonstrate that integrating multimodal data across diverse experimental settings substantially improves generalization and sensitivity, validating the approach's robustness for scalable, cross-species protein interaction inference. The source code of this study is freely available at: https://github.com/qqpigass/FREEPII.

Results

Design and workflow of FREEPII for protein-level representation learning

Figure 1 outlines the analysis workflow of FREEPII, a deep learning framework that integrates CF-MS data and sequence features to construct informative protein representations for downstream protein interaction inference. Each protein sequence is first encoded into a one-dimensional flattened FCGR vector, which is stacked across proteins to form the FCGR matrix. In parallel, a trainable protein embedding matrix is initialized, and elution profiles from all proteins are assembled into a CF-MS matrix. To address potential scale mismatches, both CF-MS and FCGR inputs are individually normalized to the [0, 1] range. The FCGR and embedding matrices are then combined via element-wise addition and subsequently concatenated with the CF-MS matrix to form a unified multimodal input for representation learning.

This multimodal input is processed through a CNN, which automatically extracts features and generates protein-level representations. Protein pair representations are then constructed by retrieving and concatenating individual representations based on predefined pairing indices, eliminating the need for additional pairwise encoders. These features are subsequently used for downstream tasks such as interaction prediction and clustering. To facilitate training, residual connections [25,26] are incorporated at both the protein and protein-pair levels to improve convergence and model stability. Furthermore, during model optimization, supervised protein embeddings transfer network-level information from complex labels into the feature space, guiding the feature extraction process toward learning inter-protein dependencies.

FREEPII delivers robust and accurate protein interaction prediction

To evaluate FREEPII's predictive power, we benchmarked its PPI classification performance against two representative CF-MS analysis tools: EPIC and SPIFFED. EPIC relies on manually engineered similarity metrics, while SPIFFED employs a deep learning framework but focuses solely on pairwise signal variation. FREEPII consistently outperforms both methods across multiple metrics, including sensitivity, specificity, MCC, and ROC AUC, on human and yeast datasets (Figure 2A). Specifically, FREEPII achieved average improvements of 0.150/0.093 in sensitivity, 0.103/0.039 in specificity, 0.250/0.135 in MCC, and 0.108/0.063 in AUC over SPIFFED for human/yeast datasets, respectively.

This performance advantage stems from FREEPII's three architectural innovations: (1) learning task-relevant features directly from data; (2) incorporating orthogonal biological information to enrich feature space; and (3) refining protein representations by incorporating network-level context through protein embeddings. Ablation experiments (Figure 2B, Figure S3) confirmed the additive value of each component. The CNN trained solely on CF-MS already surpassed traditional RF-based model. Adding sequence input further improved sensitivity by reducing false negatives arising from ambiguous or weak CF-MS signals, while protein embeddings enhanced both sensitivity and specificity, highlighting their role in strengthening representation quality. These results illustrate the effectiveness of FREEPII's modular and biologically informed architecture in resolving complex interaction patterns that remain inaccessible to existing CF-MS tools.

The discriminative power of FREEPII from both CF-MS data and protein sequences

To better understand how FREEPII combines CF-MS data and protein sequence information when predicting PPIs, we used saliency maps to examine the contribution of each input to individual predictions (see Methods). As shown in Figure 3A, FREEPII draws on both CF-MS data and sequence-based features with comparable frequency across most PPIs, suggesting that both inputs play essential and often complementary roles in the classification process. For a subset of interactions, however, FREEPII places greater emphasis on CF-MS data, while relatively few PPIs are predicted primarily using sequence information. Notably, when CF-MS input dominates, the model tends to focus on earlier elution fractions (Figure 3B), a region that often reflects interaction states, as interacting proteins tend to form larger assemblies that elute earlier. In contrast, when predictions are sequence-driven, CF-MS input contributes minimally, suggesting that interaction-relevant cues are directly embedded in the primary sequence in some cases.

This flexible use of multimodal information suggests that FREEPII adjusts its prediction strategy based on the nature of the input, rather than applying a uniform rule. While CF-MS data serve as the primary source of predictive signal, sequence-derived features provide complementary cues that help refine the model's interpretation when co-elution patterns alone are ambiguous. The ability to adaptively incorporate auxiliary sequence information helps FREEPII align its input focus with the available biological context.

FREEPII captures higher-order interaction patterns via multimodal and embedding-guided learning

To assess whether the learned protein representations capture higher-order interaction structure, we conducted both qualitative and quantitative analyses across different model configurations. Specifically, we applied *t*-distributed Stochastic Neighbor Embedding (t-SNE) to project representations into two dimensions for visual comparison, and computed pairwise cosine distances to assess structural consistency in the representation space (Figure 3C, Figure S4, S5). Protein complex annotations were used as labels in both analyses. For visual clarity, a representative subset of 9 human and 11 yeast protein complexes was selected for display.

As shown in Figure 3C, the sequence-only model (CNN-S) yielded representations that lacked alignment with complex annotations, indicating limited ability to capture complex-level organization. Adding protein embeddings (CNN-SE) markedly improved this organization, resulting in tighter intra-complex groupings and better concordance with complex labels. FREEPII further refined the feature space by integrating CF-MS data alongside sequence and embedding inputs. As shown in Figure S4 and S5, its representations showed enhanced compactness and clearer separation between complexes compared to CNN-SE. For example, in human datasets H3 and H4, FREEPII distinctly separated the 28S and 39S mitochondrial ribosomal subunits, which remained partially overlapping in CNN-SE. Similar improvements were seen in yeast, particularly for the mitochondrial large ribosomal subunit. These observations were supported by cosine distance analysis, with CNN-S showing little difference between intra- and inter-complex distances, while both CNN-SE and FREEPII exhibited significantly lower intra-complex distances. These results demonstrate that protein embeddings effectively guide the learning of sequence-based representations toward biologically meaningful groupings, and that CF-MS data further enriches these representations by capturing dynamic interaction patterns.

FREEPII clusters proteins with superior structural, functional, and spatial coherence

Building on its strong performance in PPI prediction and its ability to learn structured representations, we next evaluated FREEPII's effectiveness in identifying biologically meaningful protein clusters, in comparison with EPIC and SPIFFED. We first applied the composite score to assess the structural consistency between predicted clusters and reference protein complex annotations. FREEPII achieved the highest composite scores across both human and yeast datasets (Figure 4A), indicating superior structural resolution. To move beyond curated references, we next assessed the functional coherence of predicted clusters using the GOGO score [27], which quantifies semantic similarity in gene ontology without relying on predefined complex boundaries. FREEPII again outperformed competing models across nearly all human datasets and showed the strongest results in the yeast BP ontology (Figure 4B, Table S1), demonstrating its ability to group proteins based on shared biological roles. We further evaluated spatial coherence by computing subcellular co-localization scores for each predicted cluster. FREEPII consistently achieved the highest scores in both species (Figure 4C, Table S2), suggesting that its clustering aligns with known subcellular localization patterns. Notably, even when the protein embedding component was removed, the multimodal version of FREEPII (FREEPII(-)) still outperformed all single-modality models in clustering accuracy (Figure S6, Tables S3 and S4), highlighting the benefit of integrating heterogeneous biological information. Collectively, these findings underscore FREEPII's capacity to unify fine-grained interaction predictions with biologically coherent clustering, outperforming current CF-MS tools across multiple organizational levels.

FREEPII successfully identifies known and novel biologically relevant protein complexes

To further illustrate the practical utility of FREEPII, we present two types of case studies that demonstrate its ability to identify both well-characterized and novel protein complexes with strong biological relevance.

In the first case, FREEPII successfully reconstructed canonical complexes documented in the curated gold standard, such as the human Mediator complex and the yeast cytoplasmic ribosomal large subunit (Table 2). These predicted clusters consistently achieved high Jaccard index scores across biological replicates, markedly surpassing those generated by SPIFFED and EPIC, which often exhibited lower overlap and greater variability. Importantly, the superior overlap observed in FREEPII's predictions is not an artifact of cluster size, but a result of its enhanced capacity to resolve biologically meaningful organization within complex CF-MS datasets. In the second case, we investigated novel clusters predicted by FREEPII that are absent from the gold standard but supported by independent experimental evidence. For example, in the Y1 dataset, FREEPII identified a cluster comprising RNQ1, PBP1, PBP4, LSM12, MAK11, SLK19, and GDE1. Although this cluster has no counterpart in the reference annotations, interactions among PBP1, PBP4, and LSM12 have been previously reported [28], supporting the biological plausibility of this prediction. Similarly, in the Y4 dataset, FREEPII predicted a cluster including FAF1, IBD2, LOC1, MRM1, and RCM1, among which the interaction between LOC1 and RCM1

was recently validated by an independent study [29]. These case studies highlight FREEPII's effectiveness in recovering both established and previously unannotated protein complexes, reinforcing its value for real-world bioanalysis.

Experimental and biological heterogeneity contributes to improved generalization performance

Given the varied resolutions of currently available CF-MS data across experiments and species, it is of interest to examine whether incorporating datasets with different numbers of fractionations and species origins can improve prediction performance. To address this, we evaluated the generalization ability of FREEPII through a series of cross-prediction experiments using combinations of human and yeast datasets. Since the supervised protein embedding layer in FREEPII is tailored to fixed label structures and input dimensions, we employed a variant, FREEPII(-), that omits the embedding component to enable flexible training and testing across datasets. Specifically, we trained models on one or multiple CF-MS datasets and assessed their performance on others with distinct species and resolution characteristics.

As shown in Figure 5A, when models were trained on a single dataset (H1 or Y1), the RF-based model (RF-C) generally outperformed the CNN-based models in cross-species scenarios. This reflects a known limitation of data-driven models trained on narrow, domain-specific inputs, and underscores the relative robustness of predefined, formula-based features, which are less affected by resolution discrepancies. However, when additional datasets were incorporated during training (e.g., H1+Y1 or H1+H3+Y1), the performance of CNN-based models improved substantially. Notably, FREEPII(-) achieved the highest MCC across all test sets when trained on the most diverse dataset combination, clearly surpassing CNN-C and significantly outperforming RF-C (Figure 5A). This trend is further supported by Figure 5B, where the MCC of RF-C increased marginally by 0.05, while CNN-C and FREEPII(-) showed greater improvements of 0.15 and 0.17, respectively. These findings highlight the advantages of FREEPII(-), which combines the flexibility of data-driven feature learning with the added value of orthogonal biological information. While RF models are inherently limited by static, hand-crafted features that restrict adaptability, FREEPII(-) effectively learns transferable and biological meaningful representations. This makes it particularly well-suited for scalable, cross-species protein interaction inference when trained on datasets with diverse biological origins and experimental resolutions.

Discussion

In this study, we present FREEPII, a deep learning framework for protein-protein interaction and protein complex inference that integrates CF-MS data, protein sequences, and supervised protein embeddings to learn informative protein-level representations through an end-to-end CNN-based architecture. Across all classification metrics on both human and yeast datasets, FREEPII consistently outperformed EPIC and SPIFFED—two state-of-the-art CF-MS analysis tools—demonstrating superior sensitivity, specificity, and overall reliability in PPI prediction (Figure 2A). We also evaluated FREEPII against Tapioca [21], a recently developed tool designed for CF-MS-based PPI prediction. On our datasets, Tapioca showed limited predictive performance, broadly consistent with other handcrafted feature–based baselines such as EPIC. Given its lack of retraining capability, we report these results in the Supplementary Materials (Table S5) for completeness. Our results reveal that each input modality contributes distinct and complementary strengths to the model. CF-MS data provide dynamic signals that form the backbone of interaction inference. Sequence-based features offer orthogonal biological context, helping resolve ambiguous or weak co-elution signals. Supervised protein embeddings encode higher-order structural information from protein complex labels, guiding the feature extraction process toward capturing inter-protein dependencies (Figure 3). This multimodal strategy enables the learning of structured, discriminative protein representations that not only support superior pairwise interaction inference but also lead to biologically coherent clustering with top performance across structural, functional, and spatial benchmarks, showing its comprehensive performance (Figure 4). Overall, we demonstrate that FREEPII is a conceptually innovative and empirically supported method that significantly outperforms existing CF-MS analytical methods.

Although both FREEPII and SPIFFED adopt CNN-based architectures, FREEPII incorporates several architectural enhancements to improve computational efficiency, training stability, and representational capacity. First, FREEPII encodes features at the individual protein level rather than at the protein-pair level, allowing feature sharing across protein interactions, reducing the number of convolutional parameters by nearly half compared to SPIFFED. Second, FREEPII employs residual connections, inspired by ResNet [25] and Transformer [26], to improve gradient flow, stabilize training, and accelerate convergence. Third, FREEPII

incorporates a supervised embedding layer to guide the modelling of inter-protein dependencies. Given that FREEPII's architectural design yields clear performance improvements, we further examined whether it also confers concrete benefits in computational efficiency relative to SPIFFED. To this end, we constructed two matched CNN variants, referred to as the FREEPII-like and SPIFFED-like models, and benchmarked them under controlled conditions using CF-MS data as the sole input, consistent with the single-modality framework employed by SPIFFED (Table S6). The primary differences between these variants lie in whether convolution operates on individual proteins or protein pairs, and whether residual connections are included during training. Both models were trained under identical hardware settings (1.5 TB RAM CPU environment), and memory usage was recorded at peak training load. For time complexity, we compared the time required to reach a predefined classification accuracy threshold (0.8 for the H1 dataset), marking the transition from early training to convergence. As shown in Table S7, Table S8 and Figure S7, the FREEPII-like model consistently exhibited lower memory consumption and faster convergence across all experimental configurations. Notably, these advantages became more pronounced with increasing model complexity (e.g., 128 and 256 filters). Taken together, these results underscore the practical efficiency of FREEPII's design, showing that its streamlined architecture not only improves learning dynamics but also reduces computational cost.

In Figure 2B, we observed that CNN models outperform RF classifiers more substantially on human datasets than on yeast. This likely stems from differences in data resolution, as human CF-MS datasets contain more fractionation channels and thus yield more complex and information-rich elution profiles. Such higher-resolution data often challenge hand-crafted feature extraction used in RF classifiers, which compresses signals and can result in the loss of critical information. In contrast, CNNs learn directly from the raw input space, enabling them to retain informative patterns and maintain robust performance as data complexity increases. We also found that incorporating sequence-derived features as an additional input further improves model sensitivity, particularly by reducing false negatives when co-elution signals are weak or ambiguous. This observation aligns with previous work showing that multi-modal integration enhances model sensitivity [30,31]. FREEPII readily accommodates diverse sequence-based modalities, including gene expression profiles and structural embeddings derived from pre-trained models [32,33], underscoring its flexibility and extensibility. When combined with supervised protein embeddings, FREEPII achieves marked improvements in both sensitivity and specificity, demonstrating the value of incorporating higher-order interaction information into the representation learning process. Prior work by Singh et al. [34] also emphasized the importance of network-level information in improving PPI prediction, but their method relied on explicit manipulation of the adjacency matrix, limiting training coherence. In contrast, FREEPII integrates this information in a seamless, end-to-end manner. Beyond enhancing biological relevance, the protein embeddings also function as an internal correction mechanism: when sequence-derived features are missing, it can help impute the input [35], thereby improving model robustness and data integrity.

In FREEPII, protein embeddings are integrated with sequence-derived features through element-wise addition. This strategy enables the embeddings to reinforce biologically meaningful sequence patterns without increasing input dimensionality, allowing a more expressive feature space at no additional computational cost. While protein embeddings can improve representation learning, they may also introduce challenges. We observed that a CNN model trained solely on protein sequence inputs with protein embeddings tends to overpredict positive PPIs when evaluated on the full interaction set (including training, testing, and experimental PPIs) (Figure S8). This occurs because embedding vectors are optimized only for proteins included in the training set, resulting in uninitialized or poorly trained embeddings for unseen proteins. When inputs such as FCGR exhibit values numerically close to untrained embeddings, their additive combination during integration may introduce spurious signals, thereby compromising classification accuracy. FREEPII addresses this issue through a careful architectural choice: instead of summing CF-MS and sequence-derived features, it concatenates them to preserve the integrity and independent contribution of each modality. This design ensures that the more dynamically informative and numerically dominant CF-MS input remains the primary driver of the model's predictions, while enhanced-sequence features contribute complementary signals. As a result, the influence of untrained embeddings is substantially diminished. As shown in Figure S8, this approach produces a prediction distribution that closely aligns with the CF-MS-only baseline, supporting the robustness and reliability of FREEPII's multi-input integration strategy.

To manage the increased complexity introduced by multi-modal inputs and the added parameter burden from protein embeddings (Table S9, Table S10), especially under limited training data, FREEPII incorporates several regularization strategies to improve model stability and generalizability. These include dropout, weight decay, and residual connections, which collectively help constrain the solution space and mitigate overfitting. As illustrated by the learning curves of FREEPII (Figure S9), the testing loss consistently decreases and stabilizes throughout training, with no noticeable divergence from the training loss—indicating effective regularization

and minimal overfitting. Furthermore, FREEPII attains a classification accuracy of approximately 0.9 across nearly all test datasets (Figure 2), reflecting its strong generalization performance. While a slightly larger gap between training and testing loss is observed for the yeast dataset relative to the human data, this is likely attributable to the yeast dataset's smaller size and simpler interaction space. Nonetheless, the results consistently indicate that FREEPII maintains robust generalization. The effectiveness of the regularization strategies also supports the potential for extending FREEPII to a deeper architecture in future work without sacrificing generalization.

FREEPII's top performance in multi-level clustering benchmarks suggests its ability to recover protein groupings that align with known biological organization (Figure 4A). Given that protein sequences are the key input of FREEPII, we further evaluate the structural confidence of the predicted clusters using AlphaFold 3 [36,37]. We use the ipTM score to assess the prediction confidence, as it reflects the accuracy of the predicted relative positions of subunits within a complex. To benchmark performance, we compared the average ipTM scores of FREEPII-predicted clusters against those of randomly generated clusters. As shown in Figure S10, the average iPTM score of the FREEPII-predicted clusters was significantly higher than the average of the random distribution. These results suggest that, even in the absence of other structural information such as bond angles, modifications, sequence variants, FREEPII can group sequence-related proteins into clusters that are more likely to form energetically stable structures rather than randomly composed interactions. In future work, we plan to incorporate attention mechanisms (which have been widely adopted in models such as Transformer [26] and AlphaFold [32,36,37]) to further enhance the learning of protein representations by considering their own context and differences from all other proteins. This approach may improve the model's ability to capture complex interaction patterns beyond the capabilities of CNNs.

Finally, our results demonstrate that combining CF-MS datasets with varying fractionation resolutions and species origins improves the generalization and sensitivity of CNN-based models, as exposure to biologically and experimentally diverse inputs promotes the learning of transferable representations and enhances predictive robustness across domains—analogous to how data augmentation improves model adaptability in vision tasks [38]. Incorporating complementary sequence-derived features further strengthens the model's ability to recover positive PPIs, which is particularly valuable for expanding experimentally testable interaction space in downstream applications. These findings demonstrate the effectiveness of FREEPII's end-to-end framework in integrating multi-modal biological data to support generalizable protein interaction discovery across diverse experimental contexts.

Conclusions

In summary, FREEPII establishes a unified computational framework that jointly leverages CF-MS data and sequence-derived features to learn discriminative protein-level representations within a coherent architectural design, enabling accurate PPI classification and biologically consistent protein complex inference. Its architecture learns from heterogeneous biological modalities while preserving the distinct contributions of each input source. The supervised protein embeddings are used to incorporate higher-order interaction context derived from complex-level annotations, extending the model's representational capacity. Architectural design choices, including residual connections and modality-aware input handling, contribute to training stability and to reliable predictions for novel interactions, respectively. The strong generalization and sensitivity observed across diverse experimental settings highlight the effectiveness of FREEPII's integrated multimodal design and data-driven feature learning for scalable and cross-species protein interaction inference. Moreover, the flexibility to incorporate diverse biological inputs positions FREEPII as a robust and extensible framework for large-scale protein interaction and complex discovery.

Methods

CF-MS dataset curation and data pre-processing

The human CF-MS datasets (PXD002892, PXD014820, and PXD015406) were downloaded from Zenodo (doi: 10.5281/zenodo.4106578), where all uploaded data were reanalysed by Skinnider and Foster using MaxQuant [17]. Among the various files corresponding to different protein quantification strategies provided by the authors, we selected those containing iBAQ intensity of chromatograms for further analysis. The yeast

(*Saccharomyces cerevisiae*) CF-MS dataset (PXD031967) was curated by ourselves, with the detailed experimental processes described in our previous publication [39]. The number of proteins, the number of fractions, and the fractionation methods for all datasets are listed in Table 1. Protein overlap between experiments on the same species is shown in Figure S1. For handling missing values, we replaced them with zeros and then removed rows containing only zero values. Subsequently, we conducted normalization to ensure the sum of each CF-MS profile equalled 1. The number of genes in each CF-MS dataset is also listed in Table 1. To standardize input dimensions across datasets with varying fraction numbers, all elution profiles were adjusted to a fixed length of 200 fractions. Zero-padding was applied only to the end of each profile, thereby preserving the original co-elution signal. This procedure enabled cross-dataset training and prediction while maintaining the biological interpretability of elution patterns.

Protein complex collection and protein pairs labelling

The human protein complex dataset was downloaded from the CORUM database [40], while the yeast protein complex dataset was downloaded from Costanzo, M. et al. [41], which was manually inspected for physical protein-protein interactions and modified to remove genetic interactions and redundant protein complexes. In total, 3614 and 575 protein complexes were documented in human and yeast, respectively. We filtered out protein complexes consisting of fewer than three genes, resulting in 2277 human and 317 yeast protein complexes. The number of proteins present in the known protein complexes for each CF-MS dataset is shown in Table 1.

Protein pairs within the same protein complex are labelled as 'positive PPIs' and protein pairs that exist between different protein complexes are labelled as 'negative PPIs'. However, positive PPIs are reclassified as negative if they lack any co-eluting characteristic (where signal multiplication for the same fraction is greater than 0.01). Protein pairs in the CF-MS data are categorized as 'experimental PPIs' if they neither fall into the positive or negative PPI categories. Only positive PPIs and negative PPIs are used for model training and evaluation.

Protein sequence collection and numerical representation

Human and yeast protein sequences were retrieved from UniProt database [42] and subsequently converted into frequency matrix chaos game representation (FCGR) [43,44] using R package 'kaos'. We set the resolution to 16 and scaling factor to 0.863271 to prevent the overlap of attractors [45,46]. Each FCGR frequency matrix was normalized so that all values ranged between 0 and 1. The frequency matrix of each of *N* proteins was then reshaped into dimension 1*256*1 and concatenated in the first dimension to form a matrix with dimension *N**256*1 as the input of the model.

Model architecture

FREEPII consists of an embedding layer, a CNN layer, and three fully connected layers, as shown in Figure S2. The embedding layer generates protein embeddings, which are directly added to the FCGR matrix. This combined matrix is then concatenated with the CF-MS matrix, forming the input for FREEPII. The input is subsequently transformed into a feature map via the CNN layer, with the original input being reintroduced into each filter channel of the feature map. Features corresponding to paired proteins in the feature map are extracted using the provided pairing indexes, followed by the subtraction of paired features. A similar process is applied to the input to get another matrix of feature differences. These two difference matrixes, derived from the input and feature map, are concatenated along the second dimension and the final dimension is flattened to form a two-dimensional matrix. This matrix is then passed through three linear layers to generate the final prediction scores for the protein pairs. Detailed parameter settings are available in the code: https://github.com/qqpigass/FREEPII.

Model training and evaluation on PPIs

During model training, subsets of positive and negative PPIs were used, maintaining a ratio of 1:1, and five-fold cross-validation was applied to obtain the average performance of the model. To classify predicted interactions as positive or negative, a hard threshold of 0.5 was set. Interactions with predicted scores less than or equal to 0.5 were defined as negative PPIs, while those above 0.5 were considered as positive PPIs. Four classification evaluation metrics were used, including Sensitivity, Specificity, Matthews Correlation Coefficient (MCC), and Area Under Curve for Receiver-Operator Characteristic (AUC of ROC), defined as follows:

$$Sensitivity = \frac{TP}{TP + FN}$$

$$Specificity = \frac{TN}{TN + FP}$$

$$MCC = \frac{TP \times TN - FP \times FN}{\sqrt{(TP + FP)(TP + FN)(TN + FP)(TN + FN)}}$$

$$AUC\ of\ ROC = 1 - Specificity\ at\ various\ threshold\ values$$

Comparison with other CF-MS analysis tools

We compare the performances of FREEPII in both PPI classification and clustering evaluation with two existing CF-MS analysis tools, EPIC and SPIFFED. For EPIC, we used its default feature extraction metrics, namely Mutual Information, Bayes Correlation, Euclidean Distance, Jaccard Score, Apex Score, to generate features of PPIs for model training. We perform a five-fold cross-validation under the conditions of data balance and a training-test ratio of 70:30 [22]. For a fair comparison, our clustering algorithm is used to generate clusters from the outputs of EPIC and SPIFFED.

Ablation study on model architecture

We conducted an ablation study to gain deeper insights into the impact of design components, including feature extraction, consideration of protein sequences, and network-level information. In this study, we employed a Random Forest (RF) model using CF-MS data as input (denoted as RF-C in this study) as the baseline model for PPI classification tasks. The depth of RF was set to 1000, and seven features-- including distance correlation, weighted cross correlation (WCC), mutual information (MI), cosine similarity, Pearson and Spearman correlation, and Kendall rank correlation-- were computed as the features of paired CF-MS profiles. These feature combinations enabled the RF model to achieve the best classification performance among those studied by Skinnider and Foster [17]. The structures of CNN-based models for ablation study are shown in Figure S2.

Visualization of feature representations of proteins labelled by protein complexes

The feature map of CF-MS data ($N*M*F$, where N represents the number of proteins, M denotes the dimension along fractions, and F indicates the number of filters) was extracted, and the dimension of filters was flattened to form a new matrix with dimension $N*M'$ (where M' represents the product of the dimension along fractions and the number of filters). Each row in this matrix represents a feature representation of a protein. Subsequently, we merged the protein feature representations with the names of the protein complexes to which they belong. To ensure visualization clarity, we selected 9 human protein complexes (28S ribosomal subunit, mitochondrial; 39S ribosomal subunit, mitochondrial; 40S ribosomal subunit, cytoplasmic; 60S ribosomal subunit, cytoplasmic; Nop56p-associated pre-rRNA complex; Nuclear pore complex; PA700 complex; Spliceosome, A complex; TRBP containing complex) and 11 yeast protein complexes (19S proteasome regulatory particle; 90S preribosome; Cytoplasmic ribosomal large subunit; Cytoplasmic ribosomal small subunit; F0/F1 ATP synthase (complex V); Kornberg's mediator (SRB) complex; Mitochondrial small ribosomal subunit; Mitochondrial large ribosomal subunit; Nuclear pore; Preribosome, large subunit precursor; RSC complex) for labelling. Duplicate proteins were filtered out before merging with the feature representation matrix. The labelled feature representation matrix was then dimensionally reduced to two dimensions via t-SNE for visualization.

Visualize feature hotspots for classifying each PPI by computing saliency maps

To assess the contributions of CF-MS data and protein sequences in FREEPII's prediction of PPIs, we employed the saliency map [47] to visualize the feature hotspots for each PPI classification. Based on the average intensity difference of the CF-MS data and protein sequences inputs, we categorized the classified PPIs into three groups: if the average intensity of the CF-MS region exceeds that of the protein sequence region by more than 0.1, the classification is labelled as CF-MS > SEQ; if it is less than -0.1, it is labelled as CF-MS < SEQ; otherwise, it is classified as CF-MS ~ SEQ. The final representation of the saliency map for each PPI category is formed by superimposing the values of the saliency map calculated for each PPI and normalizing these values to range between 0 and 1.

Generating clusters using predicted PPI scores

The pairing indexes (edges) and prediction scores (weights) are used to construct the adjacency matrix ‘A’. Proteins (nodes) without any neighbours are removed. The adjacency matrix ‘A’ is then processed through the

Markov cluster algorithm (MCL) [48,49] with the expansion and inflation parameters set to 2, iterating three times to obtain the matrix 'A_'. To consider the topological properties within the adjacency matrix, the topological overlap matrix (TOM) [50] 'I_' is calculated according to the following formula:

$$w_{ij} = \frac{l_{ij} + a_{ij}}{min\{k_i, k_j\} + 1 - a_{ij}},$$

where w_{ij} is the new weight of edge between node 'i' and node 'j', a_{ij} is the weight of edge between node 'i' and node 'j' on the 2.5th power of 'A', $l_{ij} = \sum_u a_{iu}a_{uj}$,, and $k_i = \sum_u a_{iu}$ is the node connectivity. Then matrix 'A_' and matrix 'I_' are then combined in proportions of weights 0.3 and 0.7, and weights 0.1 and 0.9, respectively, to form two importance matrices. Cosine distance matrices are calculated from these two matrices, followed by Ward hierarchical clustering. The dynamic cut-tree algorithm of the Python function 'cutreeHybrid' is used to obtain clusters, with 'minClusterSize' set to 3 and 'deepSplit' set to 3 ('deepSplit' should be set to 1 or 2 for relatively small amounts of data).

The clusters obtained from the two matrices are then combined to form a set of clusters with overlapping members. To prevent the generation of unreasonably large clusters, we limit the size of the clusters to less than 100. If any cluster exceeds this limit, the above splitting steps are repeated ensure compliance. For highly overlapping clusters, an iterative merging step is performed. The merge threshold is set to 0.25, where the denominator is the product of the sizes of two clusters, and the numerator is the square of the amount of overlap between the two clusters.

Gene function annotations and semantic similarity measurement of GO-terms

We retrieved the semantics and relationships between GO terms from the GO Consortium released on November 4, 2022. To measure the semantic similarity between GO terms of proteins within the same complex, GOGO algorithm is used [27,51]. For a GO term 't', the semantic contribution weight is calculated according to the link type and the number of child nodes, considering the semantic contribution of ancestors in GO directed acyclic graph (DAG) to 't':

$$W_e = \frac{1}{\left(c + nc(t)\right)} + d,$$

where '$nc(t)$' is the total number of child nodes for GO term 't'. The constant parameter 'c' is set to 0.67 in GOGO to ensure $0 < W_e \leqslant 1$. The constant parameter 'd' is assigned values of 0.4 and 0.3 for the '*is-a*' and '*part-of*' relationships, respectively. For each term in DAG_t, it has a semantic contribution to the target term 't', defined as the *S*-value:

$$\begin{cases} S_t(self) = 1 \\ S_t(other) = max\{W_e \times S_t(other) | other \in children(t)\} \end{cases}$$

The semantic value of GO term 't' is the summation of *S*-values in DAG_t:

$$SV(t) = \sum\nolimits_{i \in t, ancestors(t)} S_t(i)$$

Given another GO term 'k', the semantic similarity between two GO terms is defined as:

$$S_{GO}(t,k) = \frac{\sum_{i \in (ancestors(t) \cap ancestors(k))} S_t(i) + S_k(i)}{SV(t) + SV(k)}.$$

To calculate the semantic similarity between a gene G_1 with m GO terms $go_{11}, go_{12}, \ldots go_{1m}$ and a single GO term 't', the equation is as follows:

$$Sim(t, G_1) = \max_{1 \le i \le m}\left(S_{GO}(t, go_{1i})\right).$$

Given another gene G_2 with n GO terms $go_{21}, go_{22}, \ldots go_{2n}$, the functional similarity between G_1 and G_2 is:

$$Sim(G_1, G_2) = \frac{\sum_{1 \le i \le m} Sim(go_{1i}, G_2) + \sum_{1 \le j \le n} Sim\left(go_{2j}, G_1\right)}{m + n}.$$

For a protein complex, the average of the pairwise functional similarities between all genes comprising the complex is calculated.

Co-localization within protein complexes

To assess the similarity of protein localizations within a complex, we use the co-localization score as defined in the study [52]. The subcellular locations of proteins are download from UniProt database [42]. The co-localization score of a protein complex is calculated as follows:

$$Score_{co-localization} = \frac{max_i l_i}{|C|},$$

where l_i is the number of proteins of complex *C* assigned to the localization group 'i' and |*C*| is the number of proteins in the complex *C* with localization assignments. This score provides the maximum fraction of proteins within the complex that share the same localization, divided by the total number of proteins in that complex with known localizations. This metric ensures that the higher the score, the more co-localized the proteins within a complex are, indicating better functional coherence.

Structure similarity between predicted clusters and reference protein complex dataset

To evaluate the structural compositional similarity between predicted clusters and a reference protein complex dataset, we use the composite score, as described in the literature [19,53]. The composite score is the sum of three components: Overlap, Accuracy, and Maximum Matching Ratio (MMR) [53]. Overlap is defined as the percentage of predicted clusters that have an overlap score larger than 0.25 with any reference complex. The overlap score is calculated as the square of the number of overlapping proteins between the predicted cluster and the reference complex divided by the product of their sizes. Accuracy is the geometric mean of Sensitivity and PPV, where Sensitivity and PPV are calculated by the following formulas:

$$Sensitivty = \frac{\sum_{i=1}^{n} max_{j=1}^{m} t_{ij}}{\sum_{i=1}^{n} |b_i|},$$

$$PPV = \frac{\sum_{j=1}^{m} max_{i=1}^{n} T_{ij}}{\sum_{j=1}^{m} \sum_{i=1}^{n} T_{ij}}, and$$

$$Accuracy = \sqrt{Sensitivty \times PPV},$$

where'*i*' is the index of the protein complex from 1 to *n* and '*j*' is the index of the predicted cluster from 1 to *m*. t_{ij} and T_{ij} is the number of overlapping proteins between $complex_i$ and $cluster_j$, and $|b_i|$ is the size of $complex_i$. The MMR builds on the maximal matching of the overlap score for each reference complex and all the predicted clusters and is calculated by dividing the sum of the matched overlap score by the number of reference complexes.

Evaluate the structure confidence of predicted clusters by AlphaFold-Multimer

To evaluate the feasibility of predicted clusters from the perspective of protein sequences, we used the AlphaFold Server (AlphaFold3, AF3) [37] to predict the structure of selected clusters. Due to the high computational cost, we limited the evaluation to protein clusters predicted by FREEPII on the Y1 dataset, specifically those with sizes ranging from 3 and 7 proteins. We use the ipTM score to assess the prediction confidence, as it reflects the accuracy of the predicted relative positions of the subunits with a complex. The idea of our comparison is to use the average scores of randomly generated clusters as a baseline to assess whether the average scores of clusters predicted by FREEPII are significantly larger than that of randomly generated clusters. Since AF3' server limits outputs to 30 predictions per account per day, we precomputed a pool of random clusters to support statical analysis. To account for ipTM score distribution differences by cluster sizes, we first generated 20 random clusters for each size (from 3 to 7) as background samples. We then create synthetic set of random clusters with the same size distribution as the FREEPII-predicted clusters and calculated the average of ipTM score for each set. This procedure was repeated 100,000 times via a bootstrap strategy to establish a background distribution. A one-sample T test was used to assess whether the value is significantly different from the mean of the null distribution.

Abbreviations

AP-MS: affinity purification/mass spectrometry; AUC: area under the curve; BP: biological process; CC: cellular component; CF-MS: co-fractionation/mass spectrometry; CNN: convolutional neural network; DAG: directed acyclic graph; FCGR: frequency matrix chaos game representation; FN: false negative; FP: false positive; MCC: Matthews correlation coefficient; MCL: Markov cluster algorithm; MF: molecular function; MI: mutual information; MMR: maximum matching ratio; PPIs: protein–protein interactions; PPV: positive predictive value; RF: random forest; ROC: receiver operating characteristic; TN: true negative; TOM: topological overlap matrix; TP: true positive; t-SNE: t-distributed stochastic neighbor embedding; WCC: weighted cross correlation; Y2H: yeast-two-hybrid.

Acknowledgments

We gratefully acknowledge all data contributors and their submitting laboratories for generating the CF-MS data and sharing them on public resources [17,39].

Author Contributions

Y.H. worked on the conceptualization and design of the pipeline and participated in the implementation of models, analysis and visualization of results, and wrote the original draft. C.F. helped with concept development and structural modification of results as well as confirming the integrity of the analytical architecture. J.Y. and H.K. were involved in reviewing and editing of the manuscript. All authors read and approved the final manuscript.

Funding

This work was supported by Academia Sinica (AS-IA-110-L01 to J.Y. Leu) and the National Science and Technology Council (NSTC 112-2221-E-001-021-MY3 to H.K. Tsai; NSTC 113-2326-B-001-002) to J.Y. Leu), Taiwan.

Availability of Source Code and Requirements

Project name: FREEPII
Project homepage: https://github.com/qqpigass/FREEPII
Operating system(s): Platform independent
Programming language: Python
License: MIT
RRID: SCR_026316
biotoolsID: freepii
WorkflowHub DOI [54]: https://doi.org/10.48546/WORKFLOWHUB.WORKFLOW.1844.1

Data availability

The human CF-MS datasets (PXD002892, PXD014820, PXD015406) can be directly downloaded from Zenodo [55], while the yeast (*S. cerevisiae*) CF-MS datasets have been deposited to the ProteomeXchange Consortium *via* the PRIDE [56] partner repository with the dataset identifier PXD031967. Other data further supporting this work are openly available in the *GigaScience* repository, GigaDB [57].

Competing Interests

The authors declare that they have no competing interests.

Ethics approval and consent to participate

Not applicable.

Consent for publication

Not applicable.

References

1. Bludau I, Aebersold R. Proteomic and interactomic insights into the molecular basis of cell functional diversity. Nat Rev Mol Cell Biol 2020;21(6):327-340. https://doi.org/10.1038/s41580-020-0231-2.

2. Larance M, Lamond AI. Multidimensional proteomics for cell biology. Nat Rev Mol Cell Biol 2015;16(5):269-280. https://doi.org/10.1038/nrm3970.

3. Shi C, Liu F, Su X, Yang Z, Wang Y, Xie S, et al. Comprehensive discovery and functional characterization of the noncanonical proteome. Cell Res 2025;35(3):186-204. https://doi.org/10.1038/s41422-024-01059-3.

4. Cheng F, Zhao J, Wang Y, Lu W. Comprehensive characterization of protein–protein interactions perturbed by disease mutations. Nat Genet 2021;53(3):342-353. https://doi.org/10.1038/s41588-020-00774-y.

5. Lu H, Zhou Q, He J, Jiang Z, Peng C, Tong R, et al. Recent advances in the development of protein–protein interactions modulators: mechanisms and clinical trials. Signal Transduct Target Ther 2020;5(1):213. https://doi.org/10.1038/s41392-020-00315-3.

6. Huttlin EL, Bruckner RJ, Paulo JA, Cannon JR, Ting L, Baltier K, et al. Architecture of the human interactome defines protein communities and disease networks. Nature 2017;545(7655):505-509. https://doi.org/10.1038/nature22366.

7. Paiano A, Margiotta A, De Luca M, Bucci C. Yeast two-hybrid assay to identify interacting proteins. Curr Protoc Protein Sci 2019;95(1):e70. https://doi.org/10.1002/cpps.70.

8. Luck K, Kim DK, Lambourne L, Spirohn K, Begg BE, Bian W, et al. A reference map of the human binary protein interactome. Nature 2020;580(7803):402-408. https://doi.org/10.1038/s41586-020-2188-x.

9. Duarte CEM, Euclydes NC. Protein–protein interaction via two-hybrid assay in yeast. Methods Mol Biol 2024;2724:193-210. https://doi.org/10.1007/978-1-0716-3485-1_14.

10. Huttlin EL, Bruckner RJ, Navarrete-Perea J, Cannon JR, Baltier K, Gebreab F, et al. Dual proteome-scale networks reveal cell-specific remodeling of the human interactome. Cell 2021;184(11):3022-3040.e28. https://doi.org/10.1016/j.cell.2021.04.011.

11. Gnanasekaran P, Pappu HR. Affinity purification-mass spectroscopy (AP-MS) and co-immunoprecipitation (Co-IP) technique to study protein–protein interactions. Methods Mol Biol 2023;2690:81-85. https://doi.org/10.1007/978-1-0716-3327-4_7.

12. Salas D, Stacey RG, Akinlaja M, Foster LJ. Next-generation interactomics: considerations for the use of co-elution to measure protein interaction networks. Mol Cell Proteomics 2020;19(1):1-10. https://doi.org/10.1074/mcp.R119.001803.

13. McBride Z, Chen D, Lee Y, Aryal UK, Xie J, Szymanski DB. A label-free mass spectrometry method to predict endogenous protein complex composition. Mol Cell Proteomics 2019;18(8):1588-1606. https://doi.org/10.1074/mcp.RA119.001400.

14. Foster LJ, de Hoog CL, Zhang Y, Zhang Y, Xie X, Mootha VK, et al. A mammalian organelle map by protein correlation profiling. Cell 2006;125(1):187-199. https://doi.org/10.1016/j.cell.2006.03.022.

15. Locard-Paulet M, Doncheva NT, Morris JH, Jensen LJ. Functional Analysis of MS-Based Proteomics Data: From Protein Groups to Networks. Mol Cell Proteomics 2024; 23(12):100871. https://doi.org/10.1016/j.mcpro.2024.100871.

16. Guo T, Steen JA, Mann M. Mass-spectrometry-based proteomics: from single cells to clinical applications. Nature 2025;638(8052):901-911. https://doi.org/10.1038/s41586-025-08584-0.

17. Skinnider MA, Foster LJ. Meta-analysis defines principles for the design and analysis of co-fractionation mass spectrometry experiments. Nat Methods 2021;18(7):806-815. https://doi.org/10.1038/s41592-021-01194-4.

18. Stacey RG, Skinnider MA, Scott NE, Foster LJ. A rapid and accurate approach for prediction of interactomes from co-elution data (PrInCE). BMC Bioinformatics 2017;18(1):457. https://doi.org/10.1186/s12859-017-1865-8.

19. Hu LZM, Goebels F, Tan JH, Wolf E, Kuzmanov U, Wan C, et al. EPIC: software toolkit for elution profile-based inference of protein complexes. Nat Methods 2019;16(8):737-742. https://doi.org/10.1038/s41592-019-0461-4.

20. Skinnider MA, Cai C, Stacey RG, Foster LJ. PrInCE: an R/Bioconductor package for protein-protein interaction network inference from co-fractionation mass spectrometry data. Bioinformatics 2021;37(17):2775-2777. https://doi.org/10.1093/bioinformatics/btab022.

21. Reed TJ, Tyl MD, Tadych A, Troyanskaya OG, Cristea IM. Tapioca: a platform for predicting de novo protein-protein interactions in dynamic contexts. Nat Methods 2024; 21(3):488-500. https://doi.org/10.1038/s41592-024-02179-9.

22. Chen YH, Chao KH, Wong JY, Liu CF, Leu JY, Tsai HK. A feature extraction free approach for protein interactome inference from co-elution data. Brief Bioinform 2023;24(4):bbad229. https://doi.org/10.1093/bib/bbad229.

23. Yu M, Gormley MR, Dredze M. Combining word embeddings and feature embeddings for fine-grained relation extraction. In Proceedings of the 2015 Conference of the North American Chapter of the Association for Computational Linguistics: Human Language Technologies 2015;1374-1379. Denver, Colorado. ACL. https://doi.org/10.3115/v1/N15-1155.

24. Kan S, Cen Y, He Z, Zhang Z, Zhang L, Wang Y. Supervised deep feature embedding with handcrafted feature. IEEE Trans Image Process 2019;28(12):5809-5823. https://doi.org/10.1109/TIP.2019.2901407.

25. He K, Zhang X, Ren S, Sun J. Deep residual learning for image recognition. In Proceedings of the 2016 IEEE Conference on Computer Vision and Pattern Recognition (CVPR) 2016; pp. 770-778. Las Vegas, NV, USA. https://doi.org/10.1109/CVPR.2016.90.

26. Vaswani A, Brain G, Shazeer N, Parmar N, Uszkoreit J, Jones L, et al. Attention is all you need. In Advances in Neural Information Processing Systems 2017; 6000-6010. Long Beach, CA, USA. https://doi.org/10.48550/arXiv.1706.03762.

27. Zhao C, Wang Z. GOGO: An improved algorithm to measure the semantic similarity between gene ontology terms. Sci Rep 2018;8(1):15107. https://doi.org/10.1038/s41598-018-33219-y.

28. Swisher KD, Parker R. Localization to, and effects of Pbp1, Pbp4, Lsm12, Dhh1, and Pab1 on stress granules in Saccharomyces cerevisiae. PLoS One 2010;5(4):e10006. https://doi.org/10.1371/journal.pone.0010006.

29. Michaelis AC, Brunner AD, Zwiebel M, Meier F, Strauss MT, Bludau I, et al. The social and structural architecture of the yeast protein interactome. Nature 2023;624(7990):192-200. https://doi.org/10.1038/s41586-023-06739-5.

30. El Adoui M, Drisis S, Benjelloun M. Multi-input deep learning architecture for predicting breast tumor response to chemotherapy using quantitative MR images. Int J Comput Assist Radiol Surg 2020;15(9):1491-1500. https://doi.org/10.1007/s11548-020-02209-9.

31. Tsietso D, Yahya A, Samikannu R, Tariq MU, Babar M, Qureshi B, et al. Multi-input deep learning approach for breast cancer screening using thermal infrared imaging and clinical data. IEEE Access 2023;11: 52101-52116. https://doi.org/10.1109/ACCESS.2023.3280422.

32. Jumper J, Evans R, Pritzel A, Green T, Figurnov M, Ronneberger O, et al. Highly accurate protein structure prediction with AlphaFold. Nature 2021;596(7873):583-589. https://doi.org/10.1038/s41586-021-03819-2.

33. Elnaggar A, Heinzinger M, Dallago C, Rehawi G, Wang Y, Jones L, et al. ProtTrans: toward understanding the language of life through self-supervised learning. IEEE Trans Pattern Anal Mach Intell 2022;44(10):7112-7127. https://doi.org/10.1109/TPAMI.2021.3095381.

34. Singh R, Devkota K, Sledzieski S, Berger B, Cowen L. Topsy-Turvy: integrating a global view into sequence-based PPI prediction. Bioinformatics 2022;38(Suppl 1):i264-i272. https://doi.org/10.1093/bioinformatics/btac258.

35. Ghorbani A, Zou JY. Embedding for informative missingness: deep learning with incomplete data. 56th Annual Allerton Conference on Communication, Control, and Computing (Allerton) 2018;437-445. Monticello, IL, USA. IEEE. https://doi.org/10.1109/ALLERTON.2018.8636008.

36. Homma F, Huang J, van der Hoorn RAL. AlphaFold-Multimer predicts cross-kingdom interactions at the plant-pathogen interface. Nat Commun 2023;14(1):6040. https://doi.org/10.1038/s41467-023-41721-9.

37. Abramson J, Adler J, Dunger J, Evans R, Green T, Pritzel A, et al. Accurate structure prediction of biomolecular interactions with AlphaFold 3. Nature 2024;630(8016):493-500. https://doi.org/10.1038/s41586-024-07487-w.

38. Mikołajczyk A, Grochowski M. Data augmentation for improving deep learning in image classification problem. International Interdisciplinary PhD Workshop (IIPhDW) 2018; 117-122. Świnoujście, Poland. https://doi.org/10.1109/IIPHDW.2018.8388338.

39. Swamy KBS, Lee HY, Ladra C, Liu CFJ, Chao JC, Chen YY, et al. Proteotoxicity caused by perturbed protein complexes underlies hybrid incompatibility in yeast. Nat Commun 2022;13(1):4394. https://doi.org/10.1038/s41467-022-32107-4.

40. Tsitsiridis G, Steinkamp R, Giurgiu M, Brauner B, Fobo G, Frishman G, et al. CORUM: the comprehensive resource of mammalian protein complexes-2022. Nucleic Acids Res 2023;51(D1):D539-D545. https://doi.org/10.1093/nar/gkac1015.

41. Costanzo M, VanderSluis B, Koch EN, Baryshnikova A, Pons C, Tan G, et al. A global genetic interaction network maps a wiring diagram of cellular function. Science 2016;353(6306):aaf1420. https://doi.org/10.1126/science.aaf1420.

42. UniProt Consortium. UniProt: the universal protein knowledgebase in 2023. Nucleic Acids Res 2023;51(D1):D523-D531. https://doi.org/10.1093/nar/gkac1052.

43. Chan EYS, Corless RM. Chaos game representation. SIAM Review 2023;65: 261-290. https://doi.org/10.48550/arXiv.2012.09638.

44. Almeida JS, Carriç JA, Ant´ A, Maretzek A, Noble PA, Fletcher M. Analysis of genomic sequences by Chaos Game Representation. Bioinformatics 2001;17(5):429-37. https://doi.org/10.1093/bioinformatics/17.5.429.

45. Lö Chel HF, Eger D, Sperlea T, Heider D. Deep learning on chaos game representation for proteins. Bioinformatics 2020;36(1):272-279. https://doi.org/10.1093/bioinformatics/btz493.

46. Löchel HF, Heider D. Chaos game representation and its applications in bioinformatics. Comput Struct Biotechnol J 2021;19:6263-6271. https://doi.org/10.1016/j.csbj.2021.11.008.

47. Kadir T, Brady M. Saliency, scale and image description. Int J Comput Vis 2001;45:83-105. https://doi.org/10.1023/A:1012460413855.

48. Dongen S Van. Graph clustering via a discrete uncoupling process. SIAM J Matrix Anal Appl 2008;30(1): 121-141. https://doi.org/10.1137/040608635.

49. Enright AJ, Dongen S Van, Ouzounis CA. An efficient algorithm for large-scale detection of protein families. Nucleic Acids Res 2002;30(7):1575-1584. https://doi.org/10.1093/nar/30.7.1575.

50. Yip AM, Horvath S. Gene network interconnectedness and the generalized topological overlap measure. BMC Bioinformatics 2007;8:22. https://doi.org/10.1186/1471-2105-8-22.

51. Wang JZ, Du Z, Payattakool R, Yu PS, Chen CF. A new method to measure the semantic similarity of GO terms. Bioinformatics 2007;23(10):1274-1281. https://doi.org/10.1093/bioinformatics/btm087.

52. Krumsiek J, Zimmer R, Friedel CC. Bootstrapping the Interactome: Unsupervised Identification of Protein Complexes in Yeast. J Comput Biol 2009;16(8):971-87. https://doi.org/10.1089/cmb.2009.0023.

53. Nepusz T, Yu H, Paccanaro A. Detecting overlapping protein complexes in protein-protein interaction networks. Nat Methods 2012;9(5):471-2. https://doi.org/10.1038/nmeth.1938.

54. Chen Y. FREEPII. WorkflowHub. 2025. https://doi.org/10.48546/WORKFLOWHUB.WORKFLOW.1844.1

55. Skinnider M. Systematic reanalysis of co-fractionation mass spectrometry data: processed chromatograms [Data set]. Zenodo. 2020. https://doi.org/10.5281/zenodo.4106578

56. Perez-Riverol Y, Bai J, Bandla C, García-Seisdedos D, Hewapathirana S, Kamatchinathan S, et al. The PRIDE database resources in 2022: a hub for mass spectrometry-based proteomics evidences. Nucleic Acids Res 2022;50(D1):D543-D552. https://doi.org/10.1093/nar/gkab1038.

57. Chen Y, Liu C, Leu J, Tsai H. Supporting data for "Complete end-to-end learning from protein feature representation to protein interactome inference" GigaScience Database. 2025. https://doi.org/10.5524/102759

Table 1. CF-MS data information. The human CF-MS datasets (PXD002892, PXD014820, and PXD015406) were downloaded from Zenodo (doi: 10.5281/zenodo.4106578), and the yeast CF-MS dataset (PXD031967) was curated by ourselves. Data description and pre-processing steps are detailed in Materials and Methods.

Species	Accession	Experimental name	Abbreviation	Number of fractionations	Number of proteins	Number of proteins in protein complexes
Human	PXD002892	SEC2_H	H1	55	4002	1720
Human	PXD002892	SEC3_H	H2	55	4563	1909
Human	PXD014820	Ctrl	H3	61	5268	1986
Human	PXD015406	Control	H4	61	6043	2225
Yeast	PXD031967	Hsp90_20200122_H	Y1	27	2397	1089
Yeast	PXD031967	Ctrl_20200416_H	Y2	27	2753	1218
Yeast	PXD031967	Ctrl_20191126_H	Y3	27	2026	933
Yeast	PXD031967	Hsp90_20201028_H	Y4	27	2952	1309

Table 2. Two protein clusters inferred by FREEPII show high overlap with known protein complexes in different biological replicates.

Complex	Data	Model	% of overlap in complex	% of overlap in cluster	Jaccard similarity
Mediator	H1	FREEPII	**62.50**	**71.43**	**0.500**
		SPIFFED	12.50	22.22	0.087
		EPIC	12.50	5.00	0.037
	H2	FREEPII	**82.61**	**76.00**	**0.655**
		SPIFFED	13.04	13.64	0.071
		EPIC	13.04	4.48	0.034
Cytoplasmic ribosomal large subunit	Y1	FREEPII	**100.00**	**80.00**	**0.800**
		SPIFFED	62.50	36.76	0.301
		EPIC	70.00	54.90	0.444
	Y2	FREEPII	**100.00**	75.93	**0.759**
		SPIFFED	41.46	**80.95**	0.378
		EPIC	53.66	70.97	0.440

Figure 1. Analysis pipeline of FREEPII. The input of FREEPII consists of three parts, including: CF-MS data containing information of protein interactions in experiments (matrix A), protein sequence data (in FCGR form, see Methods) with conserved information between proteins (matrix B), and protein embeddings (matrix C) that encapsulate protein interaction information in protein complexes after training. To form the final input, matrix B is added to matrix C and then is concatenated with matrix A. FREEPII completes feature extraction and map creation through a convolutional layer, and generates protein feature representations by flattening the feature map along the filter dimension. The protein pair index is used as an additional input to extract and construct PPI feature representations. The residual connection strategy is used when constructing feature representations of proteins and PPIs, the later are then fed into a fully connected layer. The output, comprising predicted PPI scores together with the protein pairing index, is subsequently used for clustering analysis to predict protein complexes. Notably, N represents the number of proteins, M denotes the number of fractionations, M' is the length of the flattened FCGR of the protein sequence and protein embedding, and F indicates the length of a protein's feature representation.

Figure 2. PPI classification performance. Four scoring metrics, including Sensitivity, Specificity, MCC and AUC of ROC, are used to evaluate the performance of A) existing CF-MS analysis tools and B) models with various structures and inputs in the ablation study on the PPI classification task. All results are based on predictions from the testing set. Specifically, RF-C represents the random forest model using only CF-MS features as input, CNN-C denotes CNN using solely CF-MS data as input without incorporating protein sequences, and FREEPII(-) indicates FREEPII without incorporating protein embeddings.

Figure 3. FREEPII exploits two inputs for classification and learns discriminative feature representations. A) Average difference in intensity between CF-MS and protein sequence input regions using the saliency map. This is generated by applying FREEPII to the yeast dataset Y2. It visualizes the intensity contrast between CF-MS and protein sequence input regions, derived from 10000 PPIs. B) The saliency maps depicted three categories of PPIs, CF-MS~seq, CF-MS<seq, and CF-MS>seq, based on the degree to which FREEPII considers co-elution profiles or protein sequences during prediction. The prediction results are derived from FREEPII's application to yeast dataset Y2. For clarity, the padding part of CF-MS data has been removed, and only the first 100 units of the protein sequence region are displayed to avoid compressing the CF-MS data. A grey line is manually inserted to separate the two inputs visually. C) Feature representations of proteins learned by models via t-SNE (using yeast dataset Y2). The t-SNE visualization for all human and yeast dataset are shown in Figure S4 and Figure S5, respectively. Different colors represent labels for different protein complexes. For clarity, only 11 yeast protein complexes are labelled. Cosine distances between pairs of protein feature representations within and between protein complexes are calculated, and the Kruskal-Wallis test is used to assess whether the difference in distance distribution between the two groups is significant. *ns*: $p > 0.05$, *: $p \leq 0.05$, **: $p \leq 0.01$, ***: $p \leq 0.001$, ****: $p \leq 0.0001$.

Figure 4. Cluster quality assessment. To assess the quality of clusters produced by each model's predicted PPIs, three scoring metrics including composite, GOGO, and co-localization scores are employed. (A) illustrates the evaluation through the composite score, which is the sum of overlap score (red), accuracy (blue), and MMR (green). (B) and (C) depict GOGO (BP ontology, see Methods) and colocalization scores, respectively. The Wilcoxon rank-sum test was used to assess whether the distribution of GOGO scores or colocalization scores between the two groups was significantly different. *: $p \leq 0.05$, **: $p \leq 0.01$, ***: $p \leq 0.001$, ****: $p \leq 0.0001$.

Figure 5. Cross prediction and co-training. A). Models were trained on either a single co-elution dataset (H1 or Y1) or co-trained using multiple training sets (H1+Y1 and H1+H3+Y1). The prediction performance is evaluated on their own testing set and on the testing sets of other co-elution datasets. The *x*-axis indicates the training datasets, while the panels represent the species names for the testing set. Each boxplot is the distribution of performance scores of the trained model on the testing sets across all experiments for that species. The Wilcoxon rank-sum test was used to assess whether the MCC scores between the two groups was significantly different. *: $p \leq 0.05$, **: $p \leq 0.01$, ***: $p \leq 0.001$, ****: $p \leq 0.0001$. B) The growth curve displays the average performance score of each model across all testing sets, comparing the scheme of single-training (H1/Y1) to co-training (H1+Y1, H1+H3+Y1). To ensure balanced contributions of different training sets to model learning, we down-sampled the training pairing indices from various training sets to match the smallest training set's index.

Figure 1

Click here to access/download;Figure;fig1.jpg

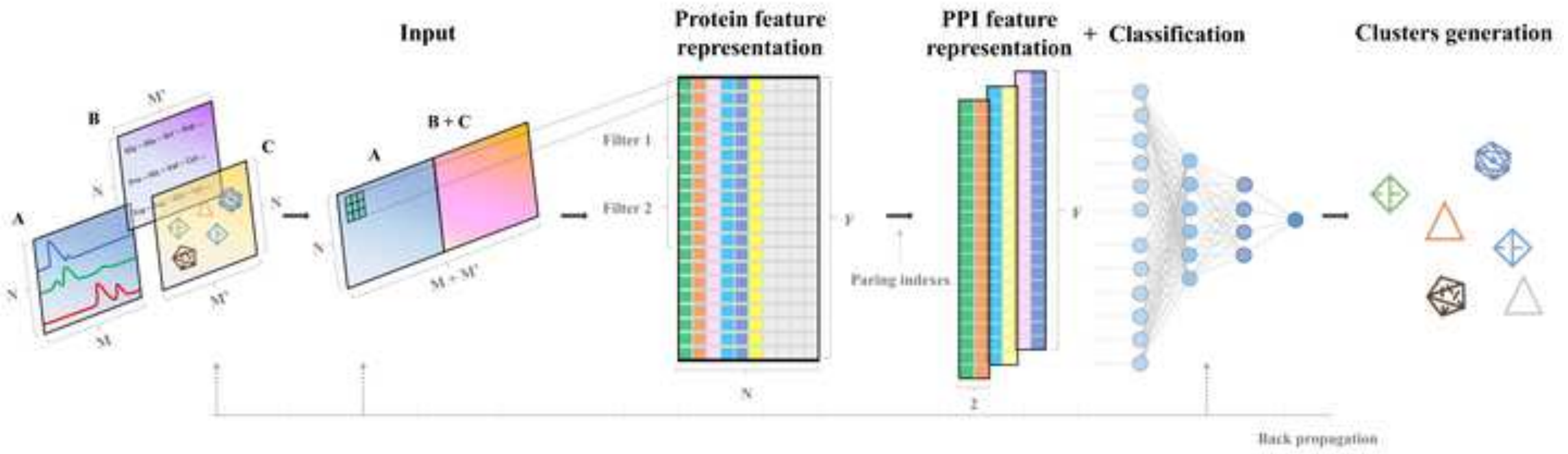

Figure 2

Click here to access/download;Figure;fig2.png

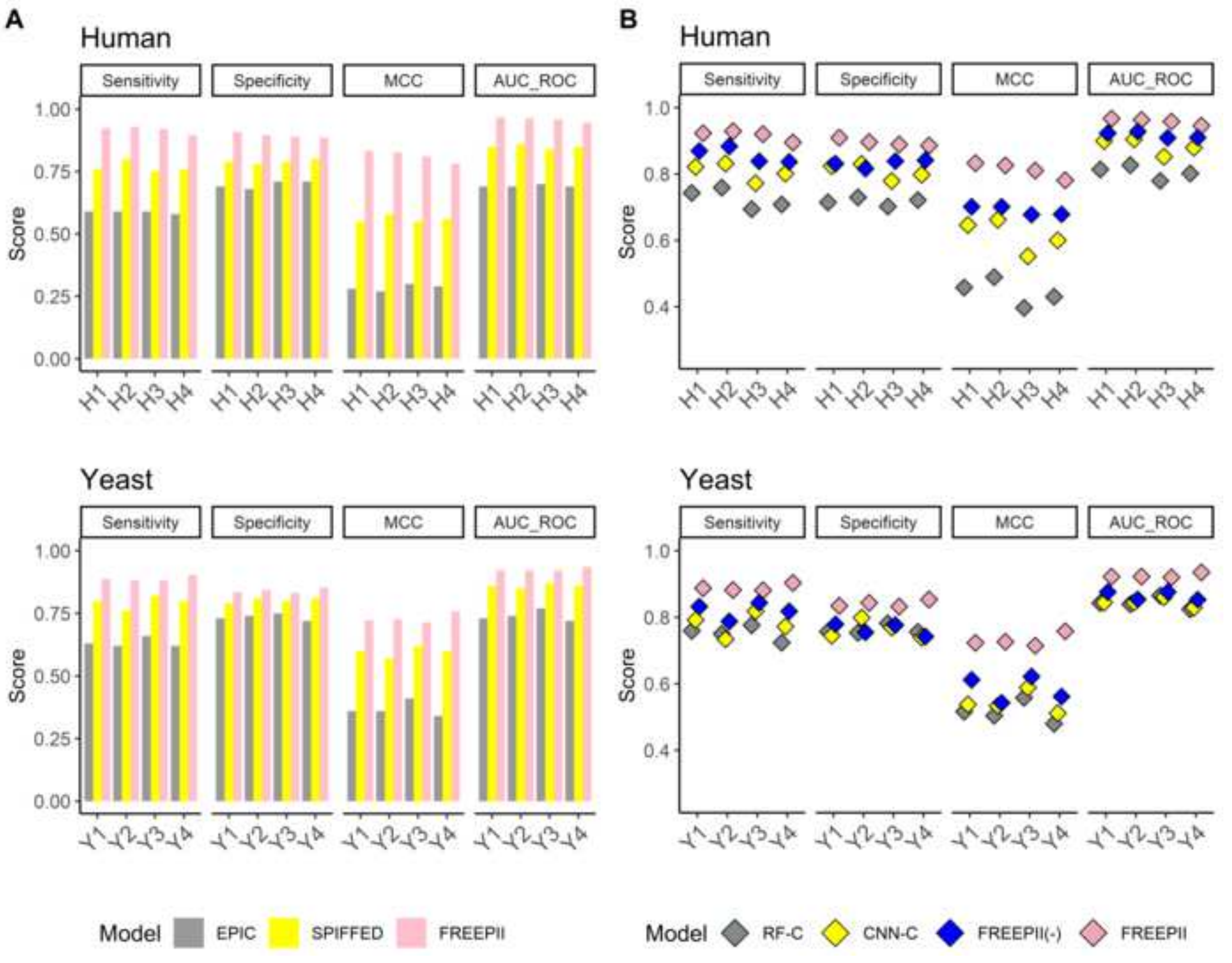

Figure 3

Click here to access/download;Figure;fig3.png

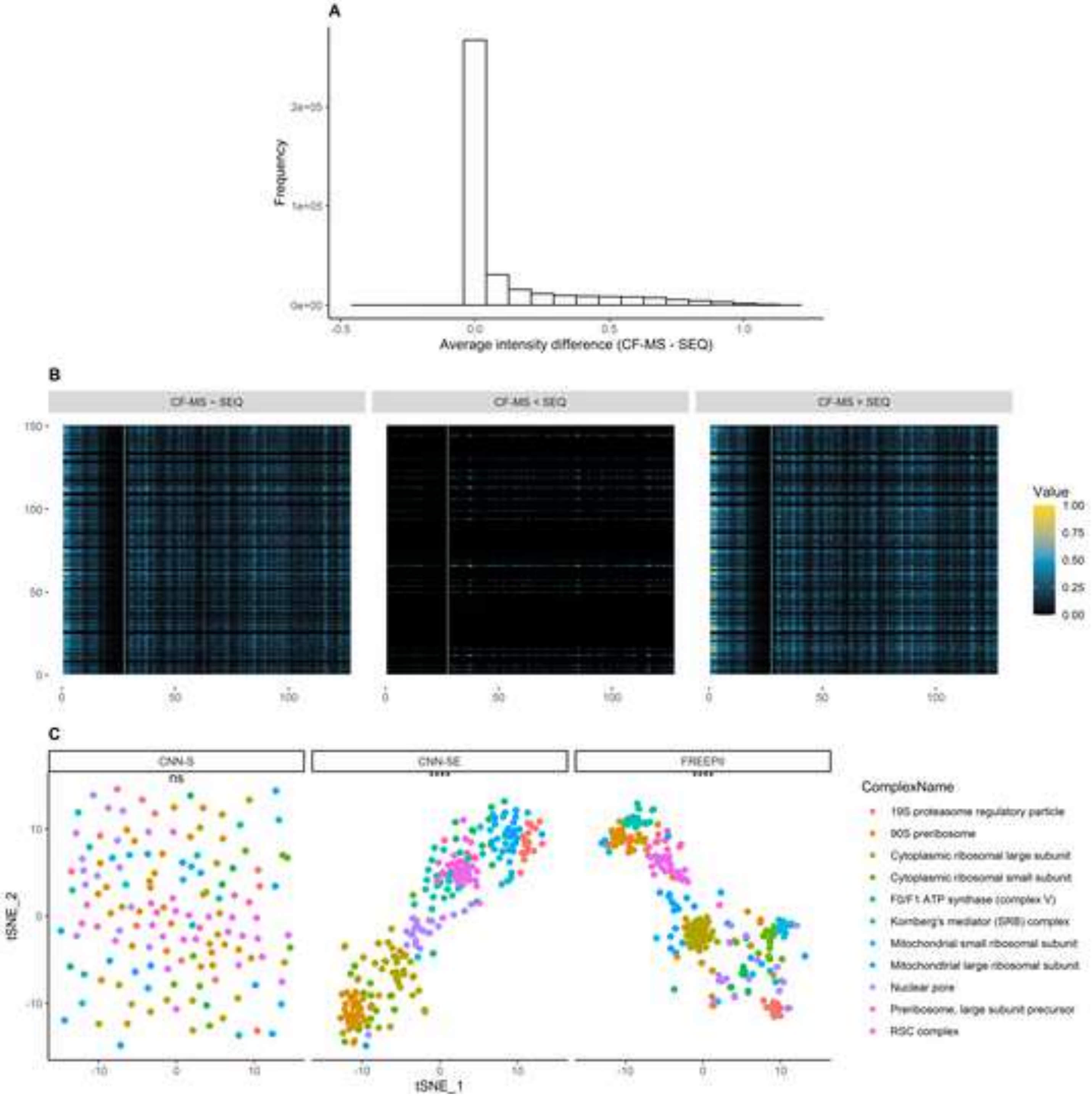

Figure 4

Click here to access/download;Figure;Figure4_new_models_stat2.png

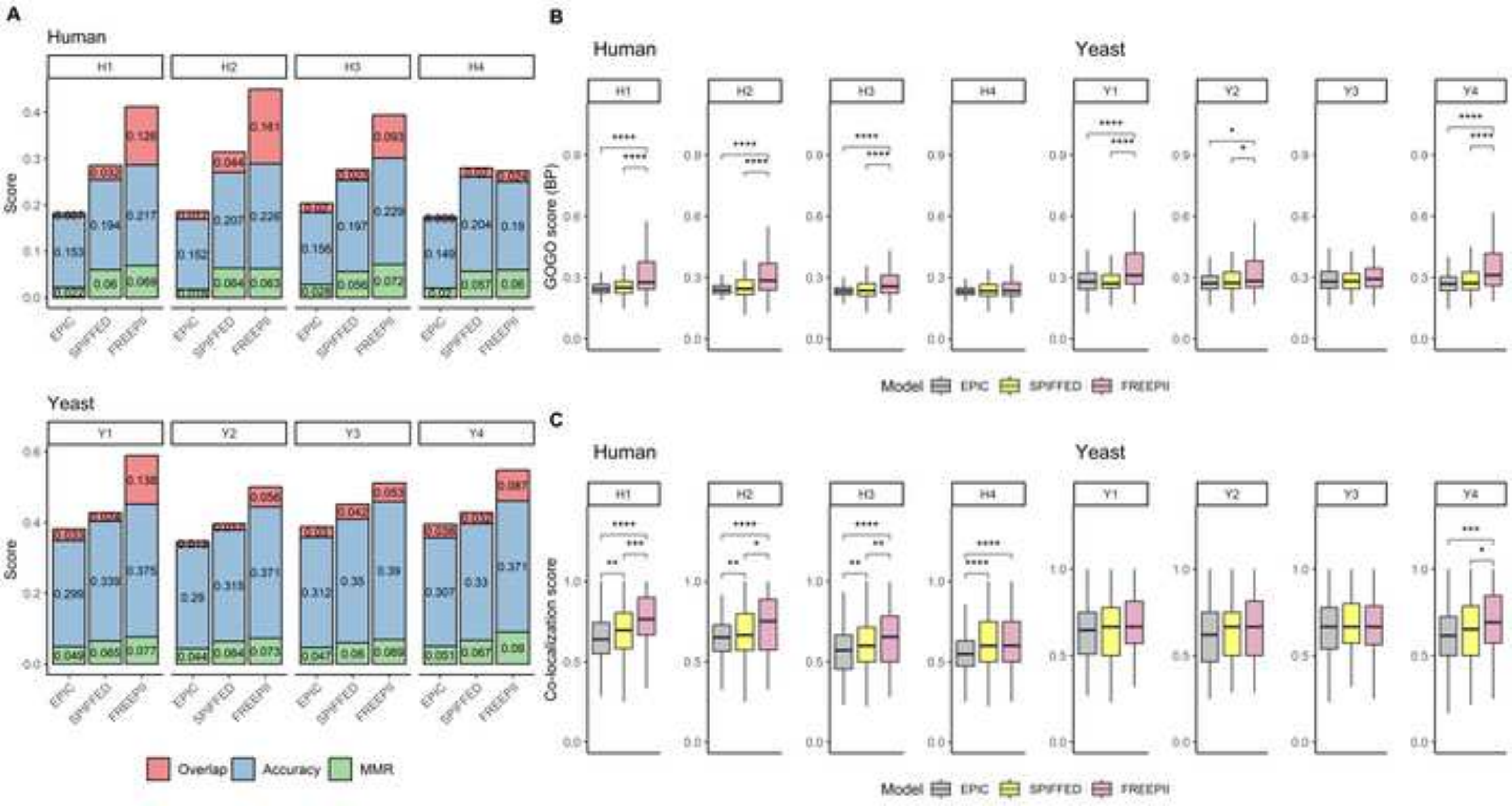

Figure 5

Click here to access/download;Figure;Figure5_new_stat.png

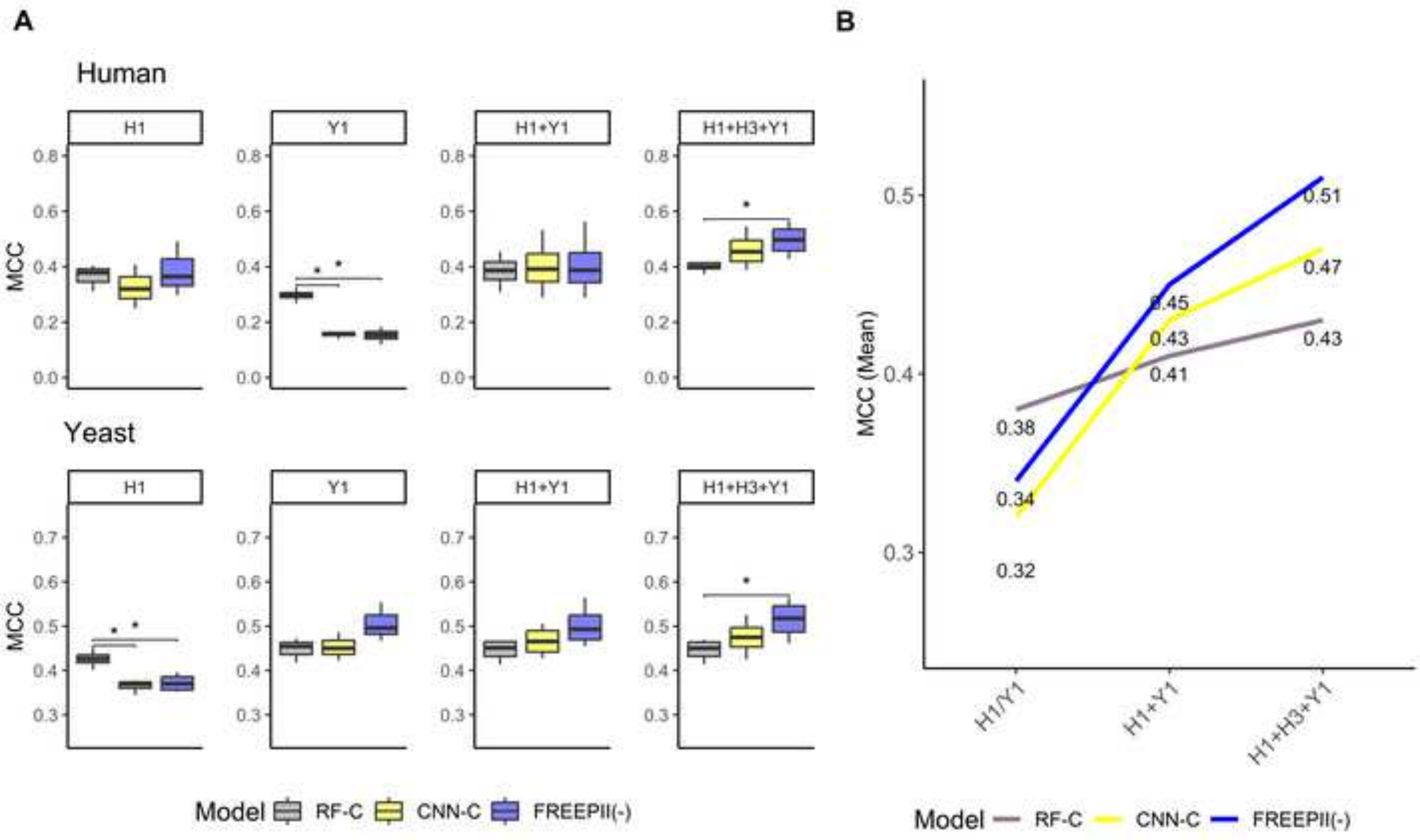

Click here to access/download
Supplementary Material
FREEPII_Supplementary Material_0903.pdf

Graphical Abstract

Click here to access/download;Graphical Abstract;Graphical abstract.jpg

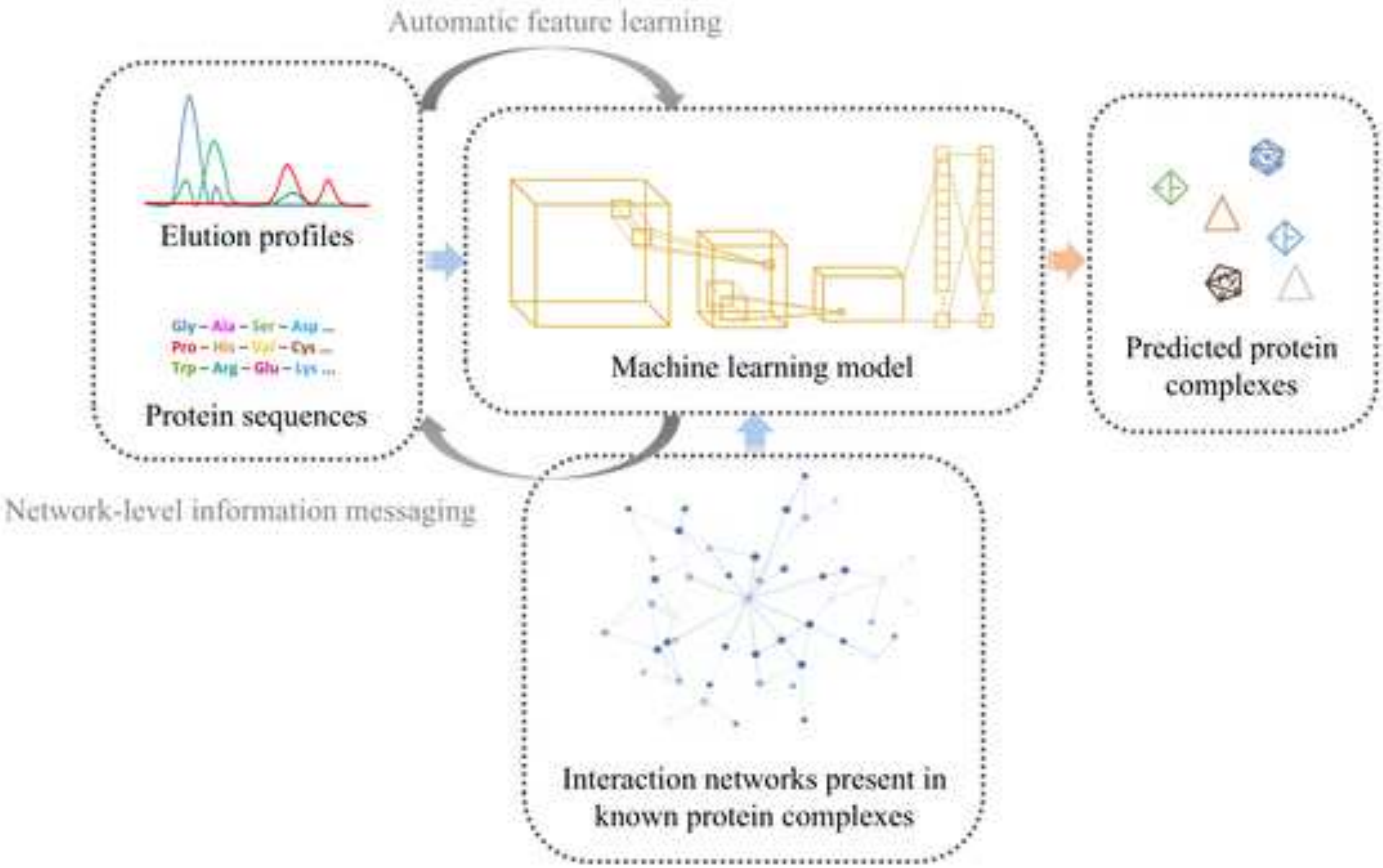

Supplement: giaf122_GIGA-D-25-00010_Revision_3 [file giaf122_giga-d-25-00010_revision_3.pdf]
