# Supplementary material for: Complete end-to-end learning from protein feature representation to protein interactome inference
Source: Gigascience. 2025 Nov 6;14:giaf122. doi: 10.1093/gigascience/giaf122 (PMC12598752; doi:10.1093/gigascience/giaf122)
Supplement: giaf122_Authors_Response_To_Reviewer_Comments_Revision_1 [file giaf122_authors_response_to_reviewer_comments_revision_1.pdf]

Author's Response To Reviewer Comments

Dear Editor,

Thank you for your continued consideration of our manuscript, "Complete end-to-end learning from protein feature representation to protein interactome inference" (GIGA-D-25-00010R1). We greatly appreciate Reviewer #2’s positive evaluation of our revised manuscript. In response to Reviewer #1’s feedback, we have carefully revised the manuscript to directly address the remaining concerns. The manuscript has undergone substantial refinement to clearly emphasize the scientific contributions, methodological advantages, and practical significance of our proposed approach (highlighted in blue). Below, we provide a detailed, point-by-point response to each of Reviewer #1’s comments.

Reviewer #1:
The manuscript serves as a standard for experimental design and writing. However, it has serious limitations, i.e., the lack of novel methodological ideas and the absence of new findings. Despite the authors' efforts to revise the manuscript, its overall quality—especially the experimental section—remains unimproved.

Response:
We appreciate Reviewer #1’s acknowledgment of our manuscript’s experimental design and writing quality. However, we respectfully note that Reviewer #1 did not previously identify specific issues concerning the experimental section, making it difficult to understand the current criticism. Moreover, we would like to emphasize that FREEPII successfully identified several novel protein complexes that were not detected by other state-of-the-art methods, as previously detailed in our response to Reviewer #2’s comment #2. Therefore, we believe it is not accurate to state that our method lacks new findings or scientific contributions.

Comment 1: More methods should be included for comparison. The current analysis only compares the proposed approach with EPIC (2019) and SPIFFED (2023, the authors' own work). To strengthen the validity of the results, comparisons with other state-of-the-art or baseline methods would be beneficial.

Response:
We would like to clarify that in our previous publication describing SPIFFED, we comprehensively compared it against EPIC and PrInCE, two of the most widely recognized handcrafted feature-based tools for CF-MS analysis. In that study, SPIFFED clearly demonstrated superior performance over these tools across all evaluation metrics for protein-protein interaction (PPI) classification. In the current manuscript, we expanded our comparative analysis by including EPIC and SPIFFED, as these represent the current benchmarks for traditional feature-based and state-of-the-art deep learning-based methods, respectively. Additionally, we evaluated recent methods such as Tapioca. However, due to Tapioca’s inability to be retrained on our datasets and its poor predictive performance (AUC-ROC of 0.652 and 0.611 on datasets H1 and Y1, respectively, versus 0.97 and 0.92 for FREEPII), we discussed its limitations qualitatively in our previous response and chose not to include it quantitatively in the main manuscript. Given these considerations, it is unclear to us which additional methods Reviewer #1 specifically expects us to include. Nonetheless, in this revision, we have further clarified and strengthened our rationale for selecting EPIC and SPIFFED as the primary benchmarks in Introduction (lines 60–64 and 78-82), Results (lines 107–109), and Discussion sections (lines 223–224).

Comment 2: The framework lacks novelty or broader appeal. While it demonstrates improved performance over the authors' previous version, the overall design does not appear sufficiently innovative or engaging for a general audience. Enhancing the conceptual contribution or highlighting unique aspects of the framework could make it more compelling.

Response:
To address this, we have now explicitly highlighted several key conceptual and methodological innovations that distinguish FREEPII from existing approaches:

• Unlike conventional approaches relying on pairwise interactions, FREEPII shifts the representation to the protein-level, significantly reducing computational complexity and broadening its practical applicability.
• FREEPII uniquely integrates protein sequence–derived features as auxiliary input, enriching the feature representation and providing complementary evidence, especially when CF-MS signals alone are insufficient or ambiguous.
• FREEPII employs embedding layers to implicitly encode higher-order interactions, substantially enhancing the representational power and robustness of the learned protein features.
• The architecture incorporates residual connections to enhance training stability and utilizes modality-aware input handling to ensure reliable prediction of novel protein interactions.

These innovations collectively represent substantial advancements over existing single-modality and pairwise-interaction-based models. We have clearly articulated these contributions in multiple sections throughout the revised manuscript, including Abstract (lines 15–26), Introduction (lines 68–78, 82–84), Results (lines 91–105, 114–122, 134–138, 156–158), Discussion (lines 220–222, 225–233, 235–255, 260–274, 284–304), and Conclusions (lines 330–341).

To further underscore the practical relevance and broader biological appeal of our method, we have provided two detailed case studies (Results, lines 178–194). These examples demonstrate that FREEPII not only outperforms existing tools such as SPIFFED and EPIC in recovering known protein complexes but also successfully identifies novel protein complexes whose interactions are experimentally supported by recent literature.

We believe these revisions comprehensively address Reviewer #1’s concerns and clearly convey the conceptual novelty, methodological rigor, and practical significance of FREEPII, particularly for robust and scalable CF-MS applications.

Editorial Manager®

Cookie Preference Center

We use cookies which are necessary to make our site work. We may also use additional cookies to analyze, improve and personalize our content and your digital experience. For more information, see our Cookie Policy.

You may choose not to allow some types of cookies. However, blocking some types may impact your experience of our site and the services we are able to offer. See the different category headings below to find out more or change your settings.

Allow all

Manage Consent Preferences

Strictly Necessary Cookies

Always active

These cookies are necessary for the website to function and cannot be switched off in our systems. They are usually only set in response to actions made by you which amount to a request for services, such as setting your privacy preferences, logging in or filling in forms. You can set your browser to block or alert you about these cookies, but some parts of the site will not then work.

Functional Cookies

These cookies enable the website to provide enhanced functionality and personalisation. They may be set by us or by third party providers whose services we have added to our pages. If you do not allow these cookies then some or all of these services may not function properly.

Performance Cookies

These cookies allow us to count visits and traffic sources so we can measure and improve the performance of our site. They help us to know which pages are the most and least popular and see how visitors move around the site.

Targeting Cookies

These cookies may be set through our site by our advertising partners. They may be used by those companies to build a profile of your interests and show you relevant adverts on other sites. If you do not allow these cookies, you will experience less targeted advertising.

Cookie List

Clear

Apply Cancel

Consent Leg.Interest

Confirm my choices

Powered by onetrust
