# Supplementary material for: Complete end-to-end learning from protein feature representation to protein interactome inference
Source: Gigascience. 2025 Nov 6;14:giaf122. doi: 10.1093/gigascience/giaf122 (PMC12598752; doi:10.1093/gigascience/giaf122)
Supplement: giaf122_Authors_Response_To_Reviewer_Comments_Revision_2 [file giaf122_authors_response_to_reviewer_comments_revision_2.pdf]

Author's Response To Reviewer Comments

We thank you for handling our manuscript entitled “Complete end-to-end learning from protein feature representation to protein interactome inference” (Manuscript ID: GIGA-D-25-00010R2). We deeply appreciate the opportunity to further improve our manuscript in response to the reviewers’ comments. In this revision, we provide a detailed point-by-point response outlining the revisions and clarifications made, along with additional edits to improve clarity and transparency. We would like to emphasize that all experimental design choices were carefully made to address the specific characteristics and challenges of CF-MS analysis. We sincerely hope the Editor and the reviewers find this revised version satisfactory, and please feel free to contact me if any further information is needed.

Reviewer #1:
Comment 1: " The authors given the performance of Tapioca in the response as follow "Tapioca's inability to be retrained on our datasets and its poor predictive performance (AUC-ROC of 0.652 and 0.611 on datasets H1 and Y1". Why the results heve not been added in the content? It is necessary to consider whether the issue stems from data compatibility problems rather than inherent limitations of the method itself. Also no details on hyperparameter tuning or implementation"

Response:
In our previous response, we reported Tapioca’s performance (AUC-ROC of 0.652 and 0.611 on datasets H1 and Y1, respectively) using the pretrained model and default configuration provided by its authors. Since Tapioca does not support retraining, these results reflect its intended usage—trained on a large, diverse dataset for generalizability. Notably, our observations are consistent with those reported in Tapioca’s Supplementary Table 1, suggesting that its limited performance in CF-MS-based PPI prediction arises from methodological constraints rather than data incompatibility.
Tapioca’s internal architecture primarily relies on handcrafted features (e.g., regression and random forest), placing it in the same category as EPIC and PrInCE. In our prior work, we have thoroughly benchmarked deep-learning-based approaches against such handcrafted feature-based methods. Consistently, these models exhibit common limitations, including low sensitivity, predictive bias under imbalanced training data, and susceptibility to overfitting. This reinforces our conclusion that models relying solely on handcrafted features are generally inadequate for reliable PPI inference using CF-MS data.
Following the reviewers’ suggestion, we have now included Tapioca’s performance results in the Supplementary Materials (Table S5) for completeness and transparency, and we have added a brief discussion of these results in Discussion (Lines 226–229). While we initially chose not to highlight Tapioca in the main manuscript due to its lack of retraining capability and overlapping conclusions with other baseline tools such as EPIC, its supplementary inclusion ensures clarity and provides readers with direct access to the comparative results.

Comment 2: " How about the performance using protein embeddings generated by EMS2 or other large model."

Response:
We thank the reviewer for this insightful suggestion. Indeed, we have experimented with protein embeddings generated by large pretrained models, including ESM2 and ProtTrans. In several pilot experiments, these pretrained embeddings consistently achieved slightly superior model performance compared to flattened FCGR, suggesting that they capture richer biological information and offer representations more readily leveraged by downstream models.
However, generating embeddings from large pretrained models such as ProtTrans or ESM2 requires substantial computational resources—particularly for longer protein sequences—which becomes a limiting factor in large-scale applications. For this reason, we selected flattened FCGR as the primary sequence-based representation in this study, as it offers a favorable balance between informativeness and computational efficiency. FCGR is lightweight, length-invariant in output dimensionality, and scalable to thousands of proteins across multiple datasets.
Nonetheless, we fully recognize the value of pretrained embeddings. As described in Discussion (Lines 270-271), FREEPII is built with flexible, modality-aware input integration and is readily compatible with

alternative sequence-based representations, including pretrained embeddings, structural descriptors, or expression-based features. While exploring pretrained embeddings lies beyond the scope of the present study, we encourage their application in future use of FREEPII, especially where sufficient computational resources are available.

Comment 3: "Why the simple CNN is used? Some other networks should be compared to show the advantage of CNN."

Response:
We thank the reviewer for raising this important point regarding model selection. In addition to CNN, we explored alternative architectures in preliminary experiments, including graph convolutional networks (GCNs) and transformer-based models. These models achieved comparable performance in terms of classification metrics, with sensitivity and specificity differences generally within 0.1 and 0.2.
Our evaluation, however, considered not only on predictive accuracy but also on how well each model aligns with the structural characteristics of CF-MS data. We found that CNNs were particularly effective at capturing localized co-elution signals, which are essential for resolving partially overlapping or transient interactions. Supporting analysis, including visualization of elution profiles and quantitative comparisons (Euclidean distance, Pearson correlation), showed that CNN-based representations better preserved localized fluctuations, whereas transformer and GCN models tended to emphasize global similarity at the expense of these fine-grained patterns.
While CNNs did not always yield the single best numerical performance, we selected them because they most closely match the domain-specific signal properties of CF-MS data, provide better interpretability, reduce the risk of overfitting, and offer superior computational efficiency for large-scale applications. This rationale is further elaborated in Discussion (Lines 297-309). Looking forward, we plan to extend FREEPII with context-aware components such as attention mechanisms, where CNN layers serve as local feature extractors and transformer blocks model global dependencies. Such hybrid architectures could combine the strengths of local signal fidelity within broader contextual modeling.

Comment 4: " The element-wise addition of protein embeddings to FCGR (sequence) features (Page 12, Lines 378-379) ignores numerical scale disparities between modalities. CF-MS profiles (continuous) and FCGR matrices (discrete frequency counts) occupy vastly different ranges."

Response:
To clarify, in our implementation the element-wise addition is applied only between the protein embedding and the protein sequence FCGR representation. The resulting vector is then concatenated with the CF-MS elution profile, rather than added to it. This design preserves modality separation while enabling multimodal integration at a later stage of the network, a key architectural principle that we have emphasized throughout the manuscript (Results, Lines 95-96; Discussion, Lines 289-296). The purpose of element-wise addition between the embedding and FCGR is to enhance the expressiveness of sequence-derived frequency patterns and to compensate for potential sparsity or noise in the FCGR representation. Similar fusion strategies have been successfully adopted in prior studies (e.g. Ghorbani et al. 2018), and we have elaborated on this rationale in Discussion (Lines 277-279).
In addition, we have now made explicit in the manuscript (Results, Lines 95-96; Methods, Lines 380-381) that both CF-MS and FCGR inputs are normalized to the [0, 1] range to mitigate scale mismatches. After normalization, FCGR values are typically very small (often < 0.1), comparable to embedding values. By contrast, CF-MS features span a broader range (0.1~0.9), and therefore contribute stronger and more interpretable co-elution signals after concatenation. Under this configuration, protein embeddings serve as supplementary biological cues, complementing the dominant elution profile features. Empirically, we did not observe numerical instability or degraded performance with this approach, supporting its viability under the described conditions.
Ghorbani A, Zou JY. Embedding for informative missingness: deep learning with incomplete data. 56th Annual Allerton Conference on Communication, Control, and Computing (Allerton) 2018;437-445. Monticello, IL, USA. IEEE.

Comment 5: " CF-MS datasets are padded to a fixed size of 200 fractions (Page 12, Line 356) without

justification. Padding may distort elution profiles (e.g., adding zeros to 27-fraction yeast data)."

Response:
We appreciate the reviewer's concern regarding potential distortion from padding. To clarify, our strategy pads zeros only at the end of each elution profile, thereby preserving the original shape and integrity of the original co-elution signal. The padded portion carries no information by design and functions solely to standardize input dimensions, which is similar to zero-padding in image processing, where the padding does not alter valid features but ensures dimensional consistency.
Importantly, in single-dataset training scenarios, padding does not influence model learning, as all samples within a dataset share the same elution profile length. The main purpose of this design is to support cross-dataset training and prediction, where a unified input length is required for joint modeling across datasets with varying resolution or fraction lengths. To further evaluate this choice, we compared zero-padding with linear interpolation to resize elution profiles to a common length. Both strategies yielded similar model performance, suggesting that model robustness depends more on the inherent resolution of the data than on the specific preprocessing method. Based on these observations, we selected padding as the simpler and less intrusive solution. We have now added a more detailed description of the padding method and its rationale in the revised manuscript (Methods, Lines 360-363), to ensure clarity and reproducibility.

Comment 6: " Negative PPIs are defined as proteins from different complexes (Page 12, Lines 365-367). This ignores that proteins can interact transiently across complexes."

We thank the reviewer for highlighting this important limitation. We agree that proteins from different annotated complexes may still engage in transient interactions, which are not fully captured under our current definition of negative PPIs. Unfortunately, due to the lack of comprehensive interaction datasets covering transient interactions, defining negatives as protein pairs from different complexes remains a common and practical approach in PPI prediction studies, particularly in the CF-MS domain. This strategy has also been adopted in prior works such as EPIC, PrInCE, and Tapioca.
To increase the reliability of positive labels, we followed the strategy used in EPIC and required that positive protein pairs exhibit co-elution evidence (i.e., shared peaks in their elution profiles) before being included as true interactions (Methods, Lines 373-374). For future improvements, we plan to refine the construction of negative sets by excluding protein pairs that, although annotated as belonging to different complexes, also exhibit strong co-elution signals or are co-localized in the same subcellular compartment. Such refinement may reduce label noise and better delineate uncertain interactions.

Cookie Preference Center

We use cookies which are necessary to make our site work. We may also use additional cookies to analyze, improve and personalize our content and your digital experience. For more information, see our Cookie Policy.

You may choose not to allow some types of cookies. However, blocking some types may

impact your experience of our site and the services we are able to offer. See the different category headings below to find out more or change your settings.

Allow all

Manage Consent Preferences

Strictly Necessary Cookies

Always active

These cookies are necessary for the website to function and cannot be switched off in our systems. They are usually only set in response to actions made by you which amount to a request for services, such as setting your privacy preferences, logging in or filling in forms. You can set your browser to block or alert you about these cookies, but some parts of the site will not then work.

Functional Cookies

These cookies enable the website to provide enhanced functionality and personalisation. They may be set by us or by third party providers whose services we have added to our pages. If you do not allow these cookies then some or all of these services may not function properly.

Performance Cookies

These cookies allow us to count visits and traffic sources so we can measure and improve the performance of our site. They help us to know which pages are the most and least popular and see how visitors move around the site.

Targeting Cookies

These cookies may be set through our site by our advertising partners. They may be used by those companies to build a profile of your interests and show you relevant adverts on other sites. If you do not allow these cookies, you will experience less targeted advertising.

Cookie List

Clear

Apply Cancel

Consent Leg.Interest

Confirm my choices

Powered by onetrust
