# Supplementary material for: Complete end-to-end learning from protein feature representation to protein interactome inference
Source: Gigascience. 2025 Nov 6;14:giaf122. doi: 10.1093/gigascience/giaf122 (PMC12598752; doi:10.1093/gigascience/giaf122)
Supplement: giaf122_Reviewer_2_Report_Original_Submission — Andrew Emili -- 2/11/2025 [file giaf122_reviewer_2_report_original_submission.pdf]

Reviewer Report

Title: Complete end-to-end learning from protein feature representation to protein interactome inference

Version: Original Submission **Date:** 2/11/2025

Reviewer name: Andrew Emili

Reviewer Comments to Author:

Review of GigaScience manuscript GIGA-D-25-00010
This paper introduces FREEPII, a convolutional neural network (CNN)-based method for predicting protein-protein interactions (PPIs) and protein complexes using co-fractionation mass spectrometry data and protein sequences. The authors demonstrate that FREEPII outperforms SPIFFED and EPIC, which are existing computational methods in this domain.
While the study presents an interesting approach, several major concerns need to be addressed:
1. Potential Overfitting: The architecture of FREEPII includes an embedding layer, a CNN layer, and three fully connected layers, which suggests a large number of parameters. Given that the training data consists solely of curated protein complexes, overfitting is a significant concern, a common issue with deep learning models. The authors should explicitly report the number of parameters in FREEPII and the number of training data points used for each task. Additionally, they should clarify what measures were taken to mitigate overfitting.
2. Lack of Biological Case Studies: The benchmark tasks presented are abstract and high-level. To enhance credibility, the authors should provide two to three concrete biological examples where FREEPII successfully identifies novel protein complexes that other methods fail to detect. This would strengthen confidence in the method's real-world applicability.
3. Training Data Concerns:
o The curation of training data is critical for machine learning models. The authors rely on protein complexes from CORUM and reference 29, but reference 29 is itself a predicted dataset from the authors' lab and should not be considered a gold standard. The authors should consider cross-checking their results with CYC2008, a manually curated yeast complex dataset.
o The reported number of human protein complexes (3,614) in CORUM seems unusually high. The authors should clarify whether they included only experimentally validated complexes from low-throughput studies, rather than computationally inferred ones.
4. Comparison with AlphaFold-Multimer: Since protein sequences are a key input to FREEPII, a direct comparison with AlphaFold-Multimer would provide valuable insight into its predictive power. The authors should consider including such an evaluation.
5. Statistical Significance Reporting: The p-values are missing in key figures (e.g., Figures 4b and 5a). Statistical significance should be explicitly reported to support claims of superior performance.
6. Model Architecture Simplicity: Compared to recent deep learning models, FREEPII's architecture appears relatively simple. The authors could discuss potential improvements using transformer-based

architectures, similar to those in AlphaFold, to enhance performance.

Methods

Are the methods appropriate to the aims of the study, are they well described, and are necessary controls included? Choose an item.

Conclusions

Are the conclusions adequately supported by the data shown? Choose an item.

Reporting Standards

Does the manuscript adhere to the journal's guidelines on minimum standards of reporting? Choose an item.

Choose an item.

Statistics

Are you able to assess all statistics in the manuscript, including the appropriateness of statistical tests used? Choose an item.

Quality of Written English

Please indicate the quality of language in the manuscript: Choose an item.

Declaration of Competing Interests

Please complete a declaration of competing interests, considering the following questions:

- Have you in the past five years received reimbursements, fees, funding, or salary from an organisation that may in any way gain or lose financially from the publication of this manuscript, either now or in the future?
- Do you hold any stocks or shares in an organisation that may in any way gain or lose financially from the publication of this manuscript, either now or in the future?
- Do you hold or are you currently applying for any patents relating to the content of the manuscript?
- Have you received reimbursements, fees, funding, or salary from an organization that holds or has applied for patents relating to the content of the manuscript?
- Do you have any other financial competing interests?
- Do you have any non-financial competing interests in relation to this paper?

If you can answer no to all of the above, write 'I declare that I have no competing interests' below. If your reply is yes to any, please give details below.

I declare that I have no competing interests.

I agree to the open peer review policy of the journal. I understand that my name will be included on my report to the authors and, if the manuscript is accepted for publication, my named report including any attachments I upload will be posted on the website along with the authors' responses. I agree for my report to be made available under an Open Access Creative Commons CC-BY license (http://creativecommons.org/licenses/by/4.0/). I understand that any comments which I do not wish to be included in my named report can be included as confidential comments to the editors, which will not be published.

Choose an item.

To further support our reviewers, we have joined with Publons, where you can gain additional credit to further highlight your hard work (see: https://publons.com/journal/530/gigascience). On publication of this paper, your review will be automatically added to Publons, you can then choose whether or not to claim your Publons credit. I understand this statement.

Yes Choose an item.
